# SS-RNN: A Strengthened Skip Algorithm for Data Classification Based on Recurrent Neural Networks

**DOI:** 10.3389/fgene.2021.746181

**Published:** 2021-10-13

**Authors:** Wenjie Cao, Ya-Zhou Shi, Huahai Qiu, Bengong Zhang

**Affiliations:** ^1^ Research Center of Nonlinear Science, School of Mathematical and Physical Sciences, Wuhan Textile University, Wuhan, China; ^2^ School of Computer Science and Artificial Intelligence, Wuhan Textile University, Wuhan, China

**Keywords:** RNN, LSTM, SS-RNN, data classification, deep learning

## Abstract

Recurrent neural networks are widely used in time series prediction and classification. However, they have problems such as insufficient memory ability and difficulty in gradient back propagation. To solve these problems, this paper proposes a new algorithm called SS-RNN, which directly uses multiple historical information to predict the current time information. It can enhance the long-term memory ability. At the same time, for the time direction, it can improve the correlation of states at different moments. To include the historical information, we design two different processing methods for the SS-RNN in continuous and discontinuous ways, respectively. For each method, there are two ways for historical information addition: 1) direct addition and 2) adding weight weighting and function mapping to activation function. It provides six pathways so as to fully and deeply explore the effect and influence of historical information on the RNNs. By comparing the average accuracy of real datasets with long short-term memory, Bi-LSTM, gated recurrent units, and MCNN and calculating the main indexes (Accuracy, Precision, Recall, and F1-score), it can be observed that our method can improve the average accuracy and optimize the structure of the recurrent neural network and effectively solve the problems of exploding and vanishing gradients.

## Introduction

Data classification is one of the most important tasks for different applications, such as text categorization, tone recognition, image classification, microarray gene expression, and protein structure prediction (Choi et al., 2017; Johnson and Zhang, 2017; Malhotra et al., 2017; Aggarwal et al., 2018; Fang et al., 2018; Mikołajczyk and Grochowski, 2018; Kerkeni et al., 2019; Saritas and Yasar, 2019; Yildirim et al., 2019; Chandrasekar et al., 2020). Many types of information (e.g., language, music, and gene) can be represented as sequential data that often contains related information separated by many time steps, and these long-term dependencies are difficult to model as we must retain information from the whole sequence with greater complexity of the model (Trinh et al., 2018; Liu et al., 2019; Shewalkar, 2019; Yu et al., 2019; Zhao et al., 2020).

With the rapid development of artificial intelligence and machine learning, the recurrent neural network (RNN) models have been gaining interest as a statistical tool for dealing with the complexities of sequential data (Chung et al., 2015; Keren and Schuller, 2016; Sadeghian et al., 2019; Yang et al., 2019). In RNNs, the recurrent layers or hidden layers consist of recurrent cells, and whose states are affected by both past states and current input with feedback connections (Yu et al., 2019). However, the errors signal back-propagated through time often suffer from exponential growth or decay, a dilemma commonly referred to as exploding or vanishing gradient. To alleviate this issue, the variants of RNNs with gating mechanisms, such as long short-term memory (LSTM) networks and gated recurrent units (GRU), have been proposed. LSTMs have been shown to learn many difficult sequential tasks effectively, including speech recognition, machine translation, trajectory prediction, and correlation analysis (Elman, 1990; Jordan, 1990; Hochreiter and Schmidhuber, 1997; Schuster and Paliwal, 1997; Cho et al., 2014; Alahi et al., 2016; Zhou et al., 2016; Su et al., 2017; Gupta et al., 2018; Hasan et al., 2018; Li and Cao, 2018; Salman et al., 2018; Vemula et al., 2018; Xu et al., 2018; Yang et al., 2019). In LSTMs, the information from the past can be stored within a hidden state that is combined with the latest input at each time step, allowing long-term dependencies to be captured. In spite of this, LSTMs are unable to capture the history information far from the current time step, given that the hidden state tends to focus on the more recent past, a finding proven by Zhao et al. (2020) along with a statistical perspective.

To address this problem, several improved RNNs have been proposed (Arpit et al., 2018; ElSaid et al., 2018; Abbasvandi and Nasrabadi, 2019; Ororbia et al., 2019). For example, Gui et al. (2019) introduced a novel reinforcement learning-based method to model the dependency relationship between words by computing the recurrent transition functions based on the skip connections. Inspired by the attention mechanism, Ostmeyer and Cowell (2019) developed a new kind of RNN model by calculating a recurrent weighted average (RWA) over every past processing step (not just the preceding step) to capture long-term dependencies, which performs far better than an LSTM on several challenging tasks. Based on the RWA, Maginnis and Richemond (2017) further presented a recurrent discounted attention (RDA) model by allowing it to discount the attention applied to previous time steps in order to carry out tasks requiring equal weighting over all information seen or tasks in which new information is more important than old. Later, DiPietro et al. (2017) introduced a mixed history RNN (MIST RNN) model, a NARX (nonlinear auto-regressive with extra inputs) RNN architecture that allows direct connections from the very distant past, and showed that MIST RNNs can improve performance substantially over LSTM on tasks requiring very long-term dependencies. In addition, Zhao et al. (2020) proposed the long memory filter that can be viewed as a soft attention mechanism, and proved that long-term memory can be acquired by using long memory filter. Very recently, Ma et al. (2021) proposed an end-to-end time series classification architecture called Echo Memory-Augmented Network (EMAN), and which uses a learnable sparse attention mechanism to capture important historical information and incorporate it into the feature representation of the current time step. However, how to well balance the accuracy and efficiency by adding past time information is still difficult to solve.

In this work, we propose a new algorithm called Strengthened Skip RNN (SS-RNN) to enhance the long-term memory ability by using multiple historical information to predict the next time information. To explore the effective method for the addition of historical information, we design six models for SS-RNN to include the past information into the current moment in continuous and discontinuous ways, respectively. For each way, the additional historical information can be directly added or added by weight weighting and function mapping. To test the SS-RNN with different models, five groups of datasets (Arrhythmia dataset, Epilepsy dataset 1, Epilepsy dataset 2, Breast cancer dataset, and Diabetes dataset) were used, and we also calculated these indexes to show the classification efficiency of our model: accuracy, precision, recall, and F1-score. From the results in *Results*, it is observed that Model A with *skip* = 3 has the greatest influence on the network. The important thing is that our SS-RNN method can effectively solve the problems of exploding gradient and vanishing gradient (Gers et al., 2000; Song et al., 2018; Tao et al., 2019; Das et al., 2020; Mayet et al., 2020).

## Theoretical Model Analysis and Data Collection

### SS-RNN Model Analysis

As for RNNs, the classical LSTM cell is proposed to deal with the problem of “long-term dependencies” by introducing a “gate” into the cell to improve the remembering capacity of the standard recurrent cell.

$$\{\begin{array}{c} f_{t}=\sigma \left(W_{fh}h_{t-1}+W_{fx}x_{t}+b_{f}\right)\\ i_{t}=\sigma \left(W_{ih}h_{t-1}+W_{ix}x_{t}+b_{i}\right)\\ {\tilde{c}}_{t}=\tanh\left(W_{\tilde{c}h}h_{t-1}+W_{\tilde{c}x}x_{t}+b_{\tilde{c}}\right)\\ c_{t}=f_{t}\cdot c_{t-1}+i_{t}\cdot {\tilde{c}}_{t}\\ o_{t}=\sigma \left(W_{oh}h_{t-1}+W_{ox}x_{t}+b_{o}\right)\\ h_{t}=o_{t}\cdot \tanh\left(c_{t}\right) \end{array}$$ (1)

where $W_{fh}$ , $W_{fx}$ , $W_{ih}$ , $W_{ix}$ , $W_{\tilde{c}h}$ , $W_{\tilde{c}x}$ , $W_{oh}$ , and $W_{ox}$ are weight matrices and $b_{f}$ , $b_{i}$ , $b_{\tilde{c}}$ , $\text{and }b_{o}$ are biases of LSTM to be learned during training. The above variables can parameterize the transformations of the input gate $i_{t}$ , forget gate $f_{t}$ , and output gate $o_{t},$ respectively. σ in Eq. 1 is the sigmoid function and ⋅ stands for element-wise multiplication. $c_{t}$ denotes the cell state of LSTM. $x_{t}$ includes the inputs of LSTM cell unit, and $h_{t}$ is the hidden layer (Wang et al., 2016; Kong et al., 2017; Yu et al., 2019). One can find the mathematical models of the RNN and GRU in the Supplementary Material.

**FIGURE 1:**
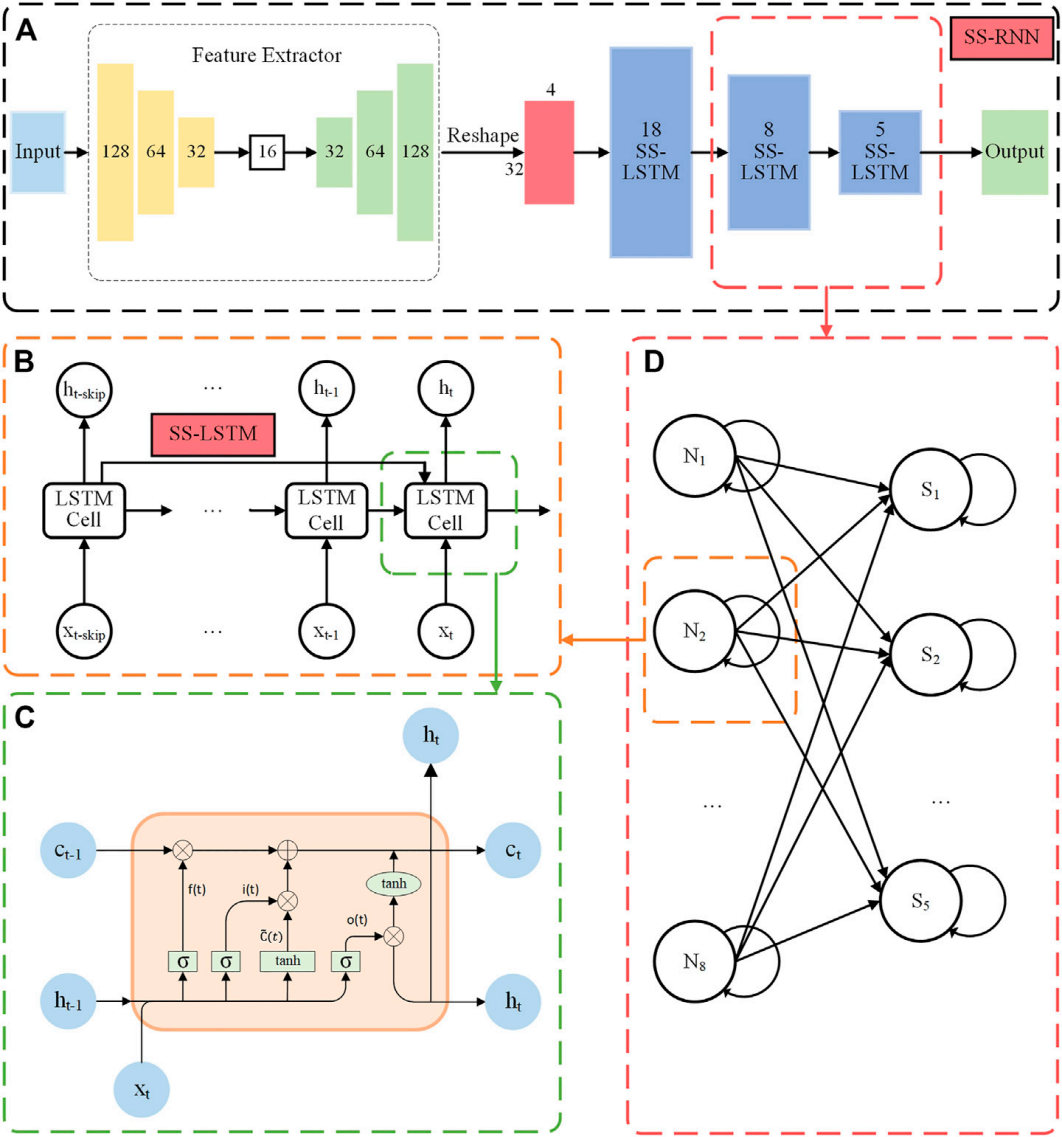
**(A)** The architecture of the SS-RNN model for data classification. **(B)** The structure of a neuron in the second SS-LSTM layer with the information of moment *t-skip* used to strengthen the long memory at the moment *t*. **(C)** The internal schematic diagram of an LSTM cell. **(D)** The structure of the second layer and the third layer of the SS-LSTM network.

Based on the LSTM model, we propose our SS-RNN model, which better utilizes historical information and could enhance the long-term memory of the model. The architecture of the SS-RNN model is shown in Figure 1. It consists of a feature extractor and a three-layer strengthened skip LSTM (SS-LSTM) network (Figure 1A). The feature extractor is added here to process the datasets with multiple features (not time series data) like the Diabetes data and Breast cancer data used in this paper. It extracts the features of multiple feature data. Then, the output of the feature extractor is reshaped to a matrix of 32*4 for further input into the SS-LSTM network (refer to Supplementary Figure S55 and Supplementary Material). For standard time series datasets, such as Arrhythmia dataset, Epilepsy dataset 1, and Epilepsy dataset 2 used in this paper, we input them to SS-LSTM directly for training. Figure 1B shows the structure of a neuron in the second layer SS-LSTM, and the information at moment of *t*-*skip* (*skip* is positive integer) is used to strengthen the memory of the moment *t*.

In comparison with the LSTM model, by adding the information from time *t −* 1, the information from the time of *t-skip* is also involved in the input at current time *t* (i.e., $x_{t}$ ). So, the SS-RNN mathematical model can be written as follows:

$$\{\begin{array}{c} f_{t}=\sigma \left(W_{fskip}h_{t-skip}+W_{fh}h_{t-1}+W_{fx}x_{t}+b_{f}\right)\\ i_{t}=\sigma \left(W_{iskip}h_{t-skip}+W_{ih}h_{t-1}+W_{ix}x_{t}+b_{i}\right)\\ {\tilde{c}}_{t}=\tanh\left(W_{\tilde{c}skip}h_{t-skip}+W_{\tilde{c}h}h_{t-1}+W_{\tilde{c}x}x_{t}+b_{\tilde{c}}\right)\\ c_{t}=f_{t}\cdot c_{t-1}+i_{t}\cdot {\tilde{c}}_{t}\\ o_{t}=\sigma \left(W_{oskip}h_{t-skip}+W_{oh}h_{t-1}+W_{ox}x_{t}+b_{o}\right)\\ h_{t}=o_{t}\cdot \tanh\left(c_{t}\right) \end{array}$$ (2)

where $W_{fskip}$ , $W_{iskip}$ , $W_{\tilde{c}skip},$ and $W_{oskip}$ are weight matrices for the corresponding inputs of the network activation functions, and $h_{t-skip}$ is the output of the moment *t-skip*.

**FIGURE 2:**
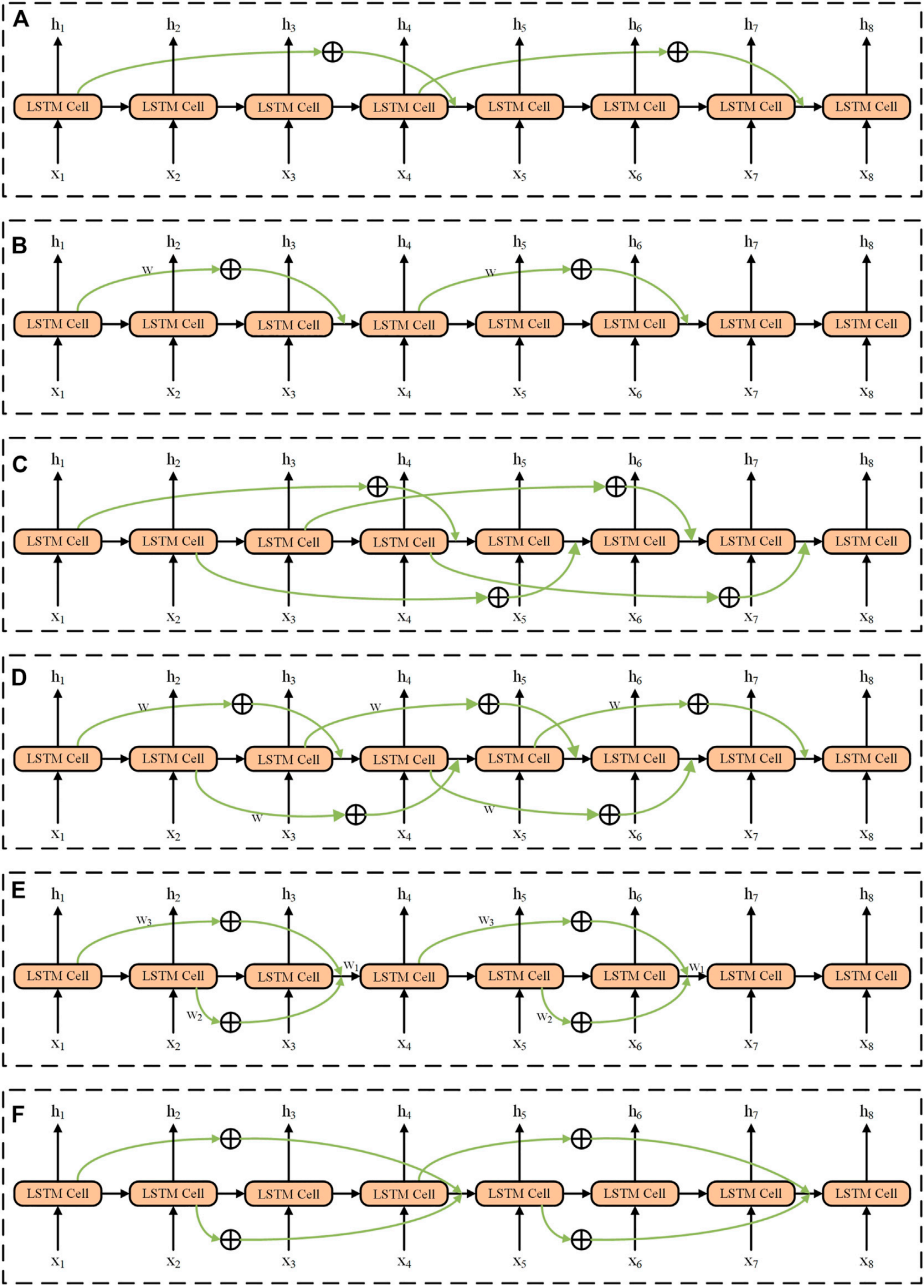
Structures of six models (e.g., *skip* = 3) used in the SS-RNN. **(A)** Model A, the method is discontinuous addition without weight weighting and function mapping. **(B)** Model B, the method is discontinuous addition with weight weighting and function mapping. **(C)** Model C, the method is continuous addition without weight weighting and function mapping. **(D)** Model D, the method is continuous addition with weight weighting and function mapping. **(E)** Model E, add all the information of the time by corresponding skip before; the method is discontinuous addition with weight weighting and function mapping. **(F)** Model F, add all the information of the time by corresponding skip before; the method is discontinuous addition without weight weighting and function mapping.

Obviously, from the above model, there are two important issues to address: 1) information of which historical moments should be involved into the current moment? 2) how should the past information be involved into the current moment? To answer these two questions, we enumerated all the methods to add the historical information to the current recurrent unit. These methods can be divided into continuous addition and discontinuous addition. The last information input consists of adding directly and weight weighting and function mapping for calculation. There are in total six models (Models A–F used in this work) for the addition of historical information, shown in Figure 2, and the detailed descriptions can be seen below (also, refer to the Supplementary Material).

Model A The information of historical moments (*t-skip*) is directly added to the current moment (*t*) and the method is discontinuous (Figure 2A). The mathematical expressions of the LSTM cell can be written as follows:

$$\{\begin{array}{c} \begin{array}{c} f_{t}=\sigma \left(W_{fh}h_{t-1}+W_{fx}x_{t}+b_{f}\right)\\ i_{t}=\sigma \left(W_{ih}h_{t-1}+W_{ix}x_{t}+b_{i}\right)\\ {\tilde{c}}_{t}=\tanh\left(W_{\tilde{c}h}h_{t-1}+W_{\tilde{c}x}x_{t}+b_{\tilde{c}}\right)\\ c_{t}=f_{t}\cdot c_{t-1}+i_{t}\cdot {\tilde{c}}_{t}\\ o_{t}=\sigma \left(W_{oh}h_{t-1}+W_{ox}x_{t}+b_{o}\right)\\ N=o_{t}\cdot \tanh\left(c_{t}\right)\\ h_{t}=\{\begin{array}{cc} N+h_{t-\mathrm{skip}}, & \mathrm{if}\;t=1+i\times \mathrm{skip}\\ N, & \mathrm{if}\;t\neq 1+i\times \mathrm{skip} \end{array} \end{array} \end{array}$$ (3)

where *skip* is the order and *i*∈**N+** (**N+** is the set of positive integers); the part marked in bold indicates that the original formula has been changed. The order of Model A in Figure 2A is 3. For example, as shown in Figure 2A, when *t* = 4 with *skip* = 3, the input of recurrent unit *h*_*4*_ comes from *h*_*1*_, *h*_*3*_ and *x*_*4*_, and *h*_*1*_ is directly added to the original output of *h*_*4*_ to form a new output of *h*_*4*_. Every three moments, additional historical information is added to the current moment.

Model B Similar to Model A, but the past information is added to the current moment after the transformation of the activation function by a weight of $W_{n}$ (Figure 2B). The corresponding mathematical expressions can be rewritten as:

$$\{\begin{array}{cc} \begin{array}{c} M=W_{fh}h_{t-1}+W_{\mathrm{fskip}}\cdot h_{\mathrm{t-skip}}\\ f_{t}=\{\begin{array}{c} \sigma \left(W_{fx}x_{t}+b_{f}+M\right),\\ \sigma \left(W_{fh}h_{t-1}+W_{fx}x+b_{f}\right), \end{array} \end{array} & \begin{array}{c} \mathrm{if}\;t=1+i\times \mathrm{skip}\\ \mathrm{if}\;t\neq 1+i\times \mathrm{skip} \end{array}\\ \begin{array}{c} N=W_{ih}h_{t-1}+W_{\mathrm{iskip}}\cdot h_{\mathrm{t-skip}}\\ i_{t}=\{\begin{array}{c} \sigma \left(W_{ix}x_{t}+b_{i}+N\right),\\ \sigma \left(W_{ih}h_{t-1}+W_{ix}x_{t}+b_{i}\right), \end{array} \end{array} & \begin{array}{c} \mathrm{if}\;t=1+i\times \mathrm{skip}\\ \mathrm{if}\;t\neq 1+i\times \mathrm{skip} \end{array}\\ \begin{array}{c} Q=W_{\tilde{c}h}h_{t-1}+W_{\tilde{c}\mathrm{skip}}\cdot h_{\mathrm{t-skip}}\\ {\tilde{c}}_{t}=\{\begin{array}{c} \tanh\left(W_{\tilde{c}x}x_{t}+b_{\tilde{c}}+Q\right),\\ \tanh\left(W_{\tilde{c}h}h_{t-1}+W_{\tilde{c}x}x_{t}+b_{\tilde{c}}\right), \end{array}\\ c_{t}=f_{t}\cdot c_{t-1}+i_{t}\cdot {\tilde{c}}_{t} \end{array} & \begin{array}{c} \mathrm{if}\;t=1+i\times \mathrm{skip}\\ \mathrm{if}\;t\neq 1+i\times \mathrm{skip} \end{array}\\ \begin{array}{c} R=W_{oh}h_{t-1}+W_{\mathrm{oskip}}\cdot h_{\mathrm{t-skip}}\\ o_{t}=\{\begin{array}{c} \sigma \left(W_{ox}x_{t}+b_{o}+R\right),\\ \sigma \left(W_{oh}h_{t-1}+W_{ox}x_{t}+b_{o}\right), \end{array}\\ h_{t}=o_{t}\cdot \tanh\left(c_{t}\right) \end{array} & \begin{array}{c} \mathrm{if}\;t=1+i\times \mathrm{skip}\\ \mathrm{if}\;t\neq 1+i\times \mathrm{skip} \end{array} \end{array}$$ (4)

When *t* = 4, the input of loop unit *h*_*4*_ comes from *h*_*1*_, *h*_*3*,_ and *x*_*4*_. After *h*_*1*_ is weighted, the function is transformed to add it to the current moment and form the output of new *h*_*4*_.

Model C It continuously adds additional historical information to the current moment in a direct addition (Figure 2C). The corresponding mathematical expressions can be rewritten as:

$$\begin{array}{c} h_{t}=o_{t}\cdot \tanh\left(c_{t}\right)+h_{t-\mathrm{skip}} \end{array}$$ (5)

The parts in bold represent changes to the original formula. The other part is the basic formula of LSTM. For example, when *t* = 4, the input of loop unit h_4_ comes from *h*_*1*_, *h*_*3*_, and *x*_*4*_, and *h*_*1*_ is directly added to the current moment to form the output of new h_4_. Model C can be regarded as the general form of Model A. In both models, the additional historical information is calculated in the same way. Model A adds historical information intermittently, and Model C adds historical information continuously where every current moment adds the historical information of the moment of *t-skip*, and which leads to a greater computational complexity for the model.

Model D It continuously adds historical information to the current moment in the form of weight weighting and function mapping (Figure 2D). The corresponding mathematical expressions can be rewritten as:

$$\begin{array}{c} \{\begin{array}{c} f_{t}=\sigma \left(W_{fh}h_{t-1}+W_{fx}x_{t}+W_{\mathrm{fskip}}\cdot h_{t-\mathrm{skip}}+b_{f}\right)\\ i_{t}=\sigma \left(W_{ih}h_{t-1}+W_{ix}x_{t}+W_{\mathrm{iskip}}\cdot h_{t-\mathrm{skip}}+b_{i}\right)\\ {\tilde{c}}_{t}=\tanh\left(W_{\tilde{c}h}h_{t-1}+W_{\tilde{c}x}x_{t}+W_{\tilde{c}\mathrm{skip}}\cdot h_{t-\mathrm{skip}}+b_{\tilde{c}}\right)\\ c_{t}=f_{t}\cdot c_{t-1}+i_{t}\cdot {\tilde{c}}_{t}\\ o_{t}=\sigma \left(W_{oh}h_{t-1}+W_{ox}x_{t}+W_{\mathrm{oskip}}\cdot h_{t-\mathrm{skip}}+b_{o}\right)\\ h_{t}=o_{t}\cdot \tanh\left(c_{t}\right) \end{array} \end{array}$$ (6)

When *t* = 4, the input of loop unit *h*_*4*_ comes from *h*_*1*_, *h*_*3*_, and *x*_*4*_, and *h*_*1*_ is directly added to the current moment to form the output of new *h*_*4*_. Model D can be regarded as the general form of Model B. In Model B and Model D, additional historical information is calculated in the same way, Model B adds historical information intermittently, and Model D adds historical information continuously.

Model E It intermittently adds additional historical information to the current moment in the form of weight weighting and function mapping (Figure 2E). The corresponding mathematical expressions can be rewritten as:

$$\{\begin{array}{c} M=W_{f1}h_{t-1}+W_{f2}h_{t-2}+W_{f3}h_{t-3}\\ f_{t}=\sigma \left(W_{fx}x_{t}+b_{f}+M\right)\\ N=W_{i1}h_{t-1}+W_{i2}h_{t-2}+W_{i3}h_{t-3}\\ i_{t}=\sigma \left(W_{ix}x_{t}+b_{i}+N\right)\\ Q=W_{\tilde{c}1}h_{t-1}+W_{\tilde{c}2}h_{t-2}+W_{\tilde{c}3}h_{t-3}\\ {\tilde{c}}_{t}=\tanh\left(W_{\tilde{c}x}x_{t}+b_{\tilde{c}}+Q\right)\\ c_{t}=f_{t}\cdot c_{t-1}+i_{t}\cdot {\tilde{c}}_{t}\\ R=W_{o1}h_{t-1}+W_{o2}h_{t-2}+W_{o3}h_{t-3}\\ o_{t}=\sigma \left(W_{ox}x_{t}+b_{o}+R\right)\\ h_{t}=o_{t}\cdot \tanh\left(c_{t}\right) \end{array}$$ (7)

When *t* = 4, the input of loop unit *h*_*4*_ comes from *h*_*1*_, *h*_*2*_, *h*_*3*_, and *x*_*4*_, and *h*_*1*_ and *h*_*2*_ are added to the current moment through weight weighting and function mapping and constitutes the output of new *h*_*4*_.

Model F It intermittently adds historical information to the current moment, and the historical information directly adds to the current moment (Figure 2F). The corresponding mathematical expressions after the improvement of LSTM can be rewritten as:

$$\begin{array}{c} \{\begin{array}{c} N=o_{t}\cdot \tanh\left(c_{t}\right)\\ h_{t}=\{\begin{array}{cc} N+\sum_{s=2}^{\mathrm{skip}}h_{t-s}, & \mathrm{if}\;t=1+i\times \mathrm{skip}\\ N, & if\;t\neq 1+i\times \mathrm{skip} \end{array} \end{array} \end{array}$$ (8)

### Data Collection

To test the effect of long-term memory introduced in this work on data classification, we first conduct experiments on three time series datasets (i.e., Arrhythmia dataset, Epilepsy dataset 1, and Epilepsy dataset 2). In addition, due to the potential correlations between the characteristics in some non-time-series biomedical data, we also perform experiments on two disease datasets: Diabetes dataset and Breast cancer dataset, to validate the ability of the model on non-time series data classification. Each dataset was split into training and testing set using the standard split. Table 1 summarizes the details of the five datasets.

**TABLE 1:** Description of five datasets used in this work for data classification.

Datasets	Source	Size	Train^a^	Test^a^	Classes^b^	Sources
Arrhythmia dataset	MIT-BIH Arrhythmia Database	109,338	87,470	21,868	5	https://www.physionet.org/content/mitdb/1.0.0/
Epilepsy dataset 1	Epileptologie Bonn	11,500	9,200	2,300	5	https://archive.ics.uci.edu/ml/datasets/Epileptic+Seizure+Recognition
Epilepsy dataset 2	CHB-MIT Scalp EEG Database	361,377	289,102	72,275	2	https://physionet.org/content/chbmit/1.0.0/
Diabetes dataset	UC Irvine Machine Learning Repository	520	416	104	2	http://archive.ics.uci.edu/ml/datasets/Early+stage+diabetes+risk+prediction+dataset
Breast cancer dataset	UC Irvine Machine Learning Repository	116	93	23	2	https://archive.ics.uci.edu/ml/datasets/Breast+Cancer+Coimbra

^a^Sizes of the training and testing sets for the five datasets, respectively.

b Number of classes of five datasets.

Arrhythmia dataset It contains 109,338 recordings of 48 half-hour excerpts of two-channel ambulatory ECG, and which have been divided into five classes based on the heart rate: one normal and four abnormal.

Epilepsy datasets There are two well-known Epilepsy datasets used in this work. One is from the Department of Epilepsy at the University of Bonn, Germany, and which contains five categories (A–E) of 100 single-channel 23.6-s segments of electroencephalogram (EEG) signals (11,500 in total). The other is from Children’s Hospital Boston including 361,377 EEG recordings from 22 epileptic patients and these recordings have been grouped into two classes.

Diabetes dataset It contains 16 features, such as age, sex, and polyuria, and the source is from the University of California at Irvine Machine Learning Repository. This has been collected using direct questionnaires from the patients at Sylhet Diabetes Hospital in Sylhet, Bangladesh, and approved by a medical doctor.

Breast cancer dataset It contains nine features from UC Irvine Machine Learning Repository (see Supplementary Material).

The original five datasets are available through the websites listed in Table 1, and we also rearranged them for the convenience of use, and which can be found in the Supplementary Material.

### Evaluation Index

For the classification task, the models are evaluated by the classification accuracy, precision, recall, and F1-score, which are defined by the confusion matrix. It is one of the most intuitive metrics used for evaluating the performance and accuracy of the model in machine learning, especially used for classification problems. The terms associated with confusion matrix can be defined as follows: True positives (TP), when the actual class of the data point is 1 and the predicted outcome is also 1. True negatives (TN) are the cases when the actual class of the given data point is 0 and the predicted result is also 0. False positives (FP) are the cases when the actual class of the data point is 0 and the predicted outcome is 1, which can be assumed that the model predicts incorrectly as the actual class is positive. False negatives (FN) are the cases when the actual class should be 1 and the predicted outcome is 0, where the model predicts incorrectly as negative. The forms are expressed as follows:

$$\begin{array}{c} \{\begin{array}{c} Accuracy=\frac{TP+TN}{TP+FP+FN+TN}\\ Precision=\frac{TP}{TP+FP}\\ Recall=\frac{TP}{TP+FN}\\ F1-score=\frac{2\times Precision\times Recall}{Precision+Recall} \end{array} \end{array}$$ (9)

## Results

### The Workflow of the SS-RNN

In SS-RNN, the information of historical moments (e.g., *t*-*skip*) can be added to the current moment (i.e., *t*) to accurately classify sequential data with long-term dependences. To determine the best methods of the past information addition and verify the effectiveness of the SS-RNN model, we did six groups of comparison experiments on five datasets, respectively. The six different models (Models A–F) and five datasets are shown in *Theoretical Model Analysis and Data Collection*. For each experiment, there are three steps: data preprocessing, training, and test.

Data preprocessing Outliers and missing values often appear in the dataset, whereas the network model cannot process those data samples. We first fill the missing values with the mean of the variable and delete the samples with outliers, which can be judged from the method of Anomaly Detection. The pre-processed time series datasets (e.g., Arrhythmia and Epilepsy datasets in Table 1) can be directly input into the SS-LSTM model. However, for the non-time series data with multiple features and different dimensions (e.g., Diabetes and Breast cancer datasets used in this work), after the above preprocessing, it needs to be fed into the feature extractor to obtain a new set of data and their characters, which can be further transformed to a matrix of 32*4 as input into the SS-LSTM. Taking the Diabetes dataset as an example, we also give detailed descriptions in the Supplementary Material.

Training For each dataset (Table 1), the training set is used to train the model. The optimized parameters of the network are as follows: dimensions of the network are 128, 64, 32, and 16, respectively (Figure 1A). For each dataset, the configuration of the SS-LSTM model is implemented in Pytorch using Eqs 3–8, and the dimensions for the three layers of the SS-LSTM model are 18, 8, and 5, respectively. The activation function is *tanh*, and the training algorithm is stochastic gradient descent with a learning rate of 0.01 and a training epoch of 50. Here, we used the cross-entropy loss as the objective function for training the network:

$$\text{Loss}=-\sum_{i=1}^{n}y_{i}\log\left(\hat{y_{i}}\right)$$ (10)

where $y_{i}$ is the true value, and $\hat{y_{i}}$ is corresponding predicted value. The batch size of each dataset after fine-tuning is shown in the Supplementary Material.

Test For each dataset, 25 different comparative experiments were performed using different structures of LSTM. One of the experiments adopted the ordinary LSTM, while the others used SS-LSTM with different models (i.e., Models A–F in Figure 2). For each model, the values of *skip* were set as 2, 3, 4 and 5, respectively. Furthermore, we also used the other classical models (LSTM, GRU and Bi-LSTM) to create the classification set for three of the datasets (i.e., Arrhythmia, Epilepsy 1 and Diabetes), and made a comparison with our SS-RNN model.

### Testing the Models With Data

To test the effect of the addition of past information on the data classification, we used our network with six different SS-LSTM models (Models A–F; Figure 2) to classify the data for five datasets (Table 1), respectively. For each SS-LSTM model, different values of *skip* (e.g., *skip* = 2, 3, 4, 5) were used. As shown in Supplementary Figures S1–S10, S12–S17, S19–S24, S26–S31 in the Supplementary Material, the loss functions calculated by Eq. 10 for the experiments in this work always converged before 50 steps, indicating that 50 steps are sufficient for the training and test processes.

#### Epilepsy Dataset 1

**FIGURE 3:**
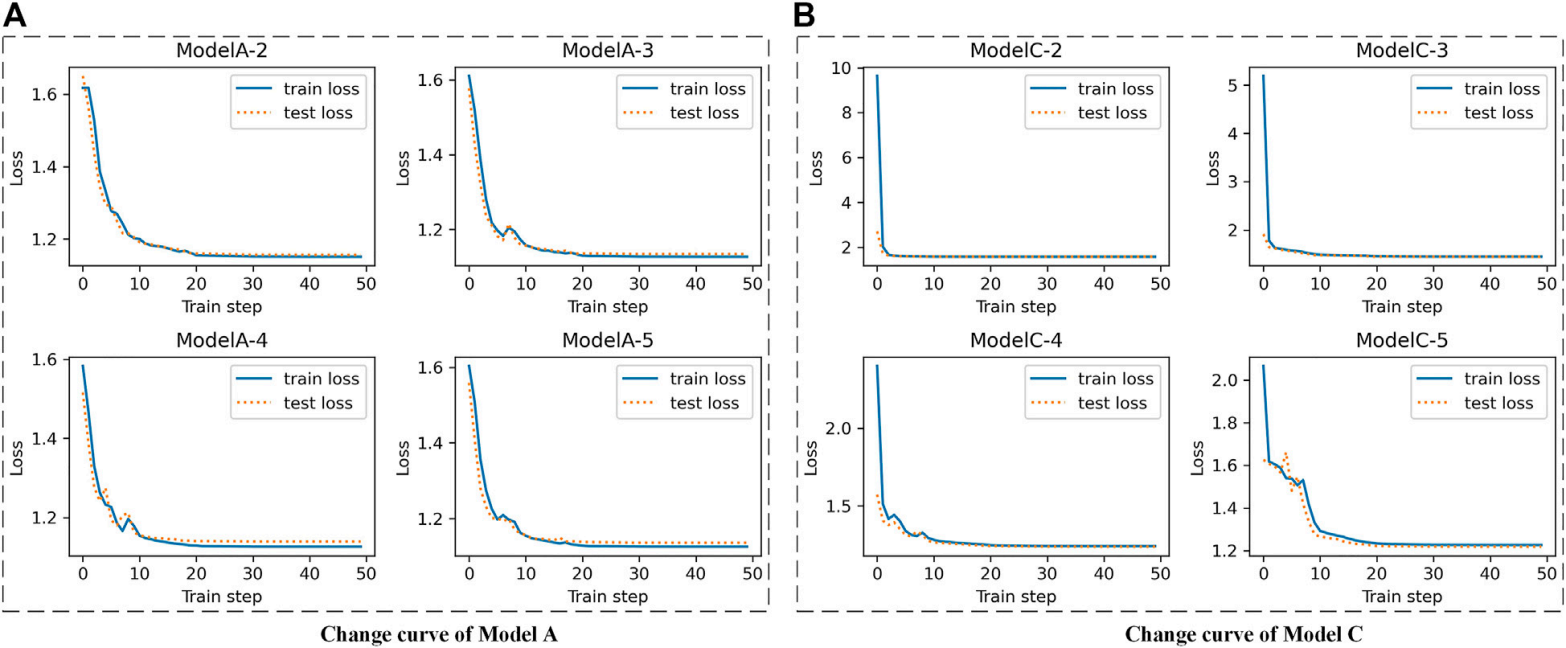
The change curves of loss function between train set and test set with different skip value of Epilepsy dataset 1. e.g., Model A-2 is Model A **(A)** with *skip* = 2, Model C-4 is Model C **(B)** with *skip* = 4.

**FIGURE 4:**
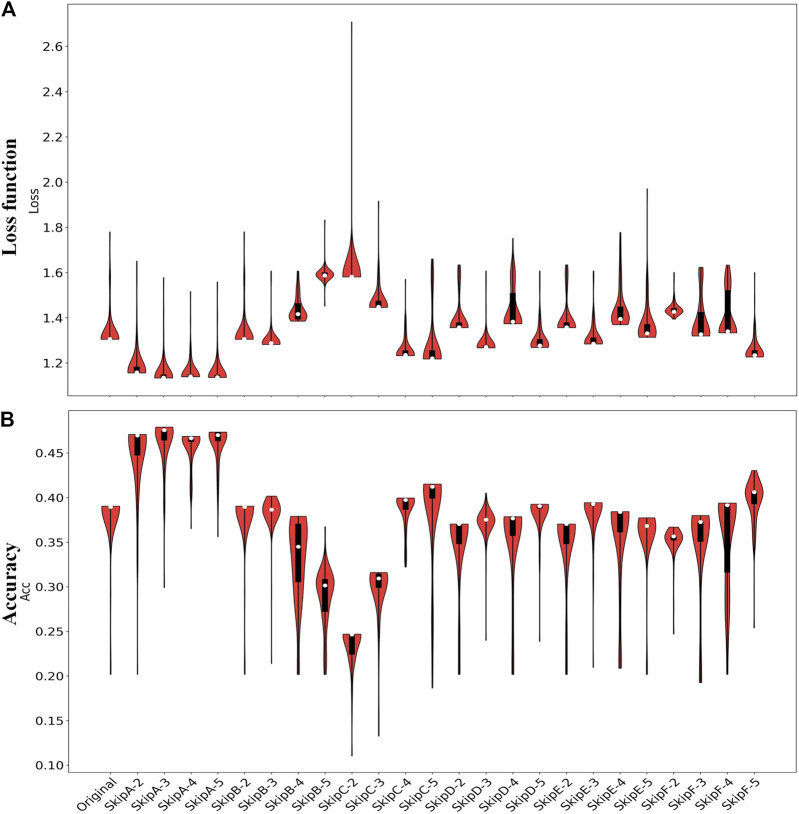
The loss functions **(A)** and predicted accuracy **(B)** of each model for classification of Epilepsy dataset 1. Original represents the results from the original LSTM, and others represent the results from the SS-LSTM with different models, e.g., SkipA-2 is Model A with *skip* = 2 (Figure 2). For each violin in **(A, B)**, the top of the black rectangle is the three-quarter digit, the bottom is the quarter digit, the white dots are the mean, and the width of the orange area is the distribution of density.

For the Epilepsy dataset, 19,200 samples were used to train our SS-LSTM, which were further tested by the rest of the samples. The loss functions show that Models A and C are more stable than the others, and the loss value of the training set is consistent with the test set, indicating that no overfitting has occurred (Figure 3, Supplementary Figures S1–S4). As shown in Figure 4, the value of loss function of Model A is also the lowest among all models (Figure 4A), and the predicted accuracy of Model A is ∼47%, which is not only higher than that (∼37%) of the original LSTM, but also significantly better than those predicted by SS-LSTM with other models (e.g., ∼40% of Model C with *skip* = 4, i.e., Model C-4). The results indicate that the past information (*t-skip*) directly added to the current moment (*t*) could effectively improve the classified accuracy on the Epilepsy dataset 1. However, Model C with *skip* = 2 has the lowest predicted accuracy (∼24%), and which could suggest that Model C is not suitable for processing this dataset.

#### Diabetes Dataset

**FIGURE 5:**
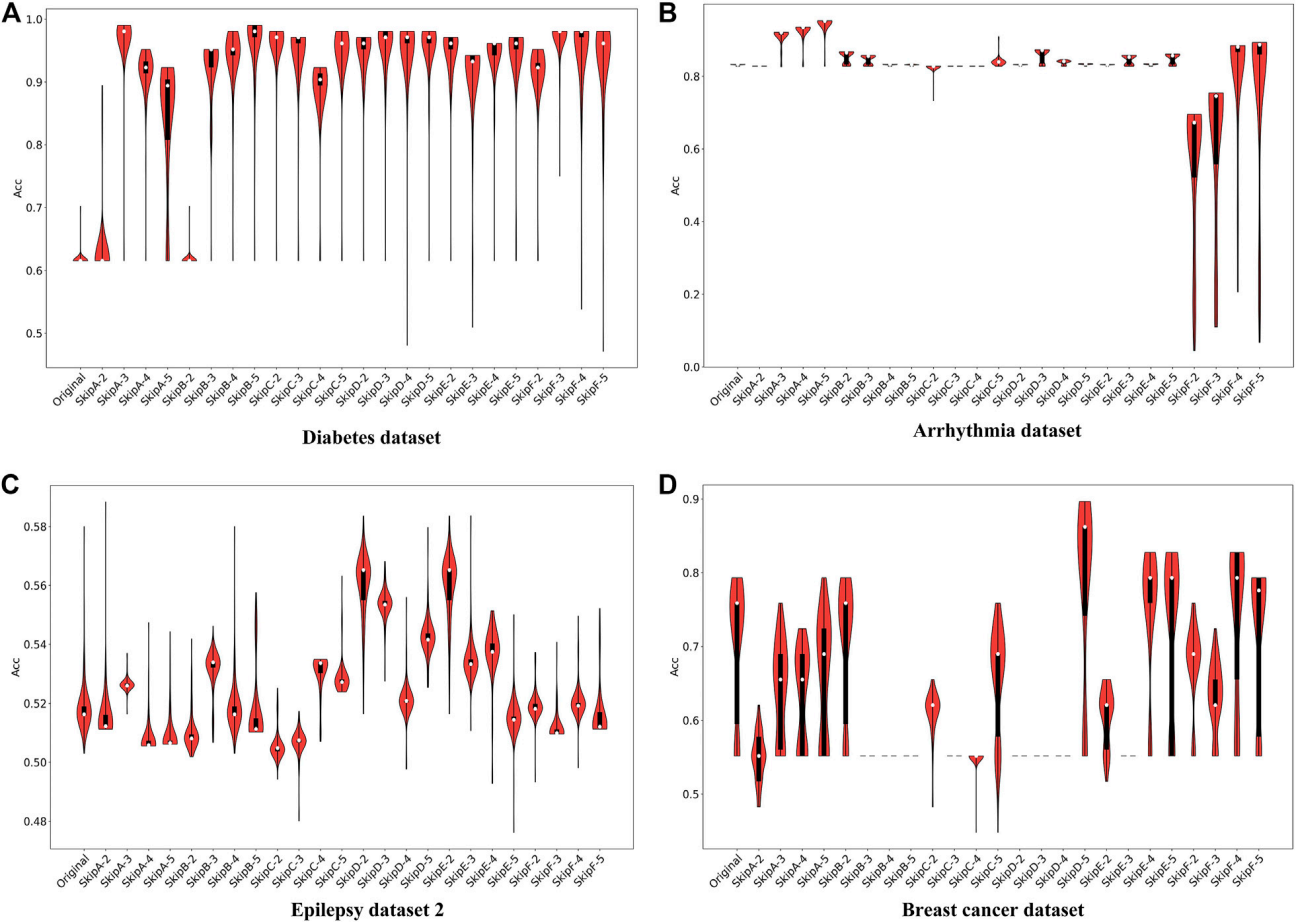
The predicted accuracy of each model for classification on Diabetes dataset **(A)**, Arrhythmia dataset **(B)**, Epilepsy dataset 2 **(C)** and Breast cancer dataset **(D)**.

As shown in Figure 5A, the predicted accuracy of most SS-LSTM models is much higher than that (∼61%) of the original LSTM model for the Diabetes dataset, and Model A with *skip* = 3 has the highest accuracy (∼98%). The accuracy of Model B is significantly and positively correlated with the order. The change curve of the loss function of each model in the training process is also shown in the Supplementary Material.

#### Arrhythmia Dataset and Other Datasets

For the Arrhythmia dataset, the long-term memory in Model A can markedly improve the classification accuracy, e.g., the ACC increases from ∼82 to 94%, as *skip* increasing from 2 to 5 (Figure 5B). Surprisingly, although the large values of *skip* can also be helpful for Model F, the ACC of Model F with any *skip* values is obviously lower than the original LSTM. Furthermore, in the other models (i.e., Model B, Model D, and Model E), no matter how the *skip* changes, the accuracy stays at the same level (∼82%), which suggests that the addition of past information could be a burden for the RNN and has no positive effect on data classification (Figure 5B). The structure of Models B and D, which both have a common characteristic that adopts the same way of weight weighting and function mapping to put the historical information added to the current time damage the dynamic performance of the RNN. So, this is not an ideal method for the Arrhythmia dataset.

We also have experiments on Epilepsy dataset 2 and Breast cancer dataset, and the relevant results and analysis are shown in the Supplementary Material.

### Comparison Results With Other Models

**FIGURE 6:**
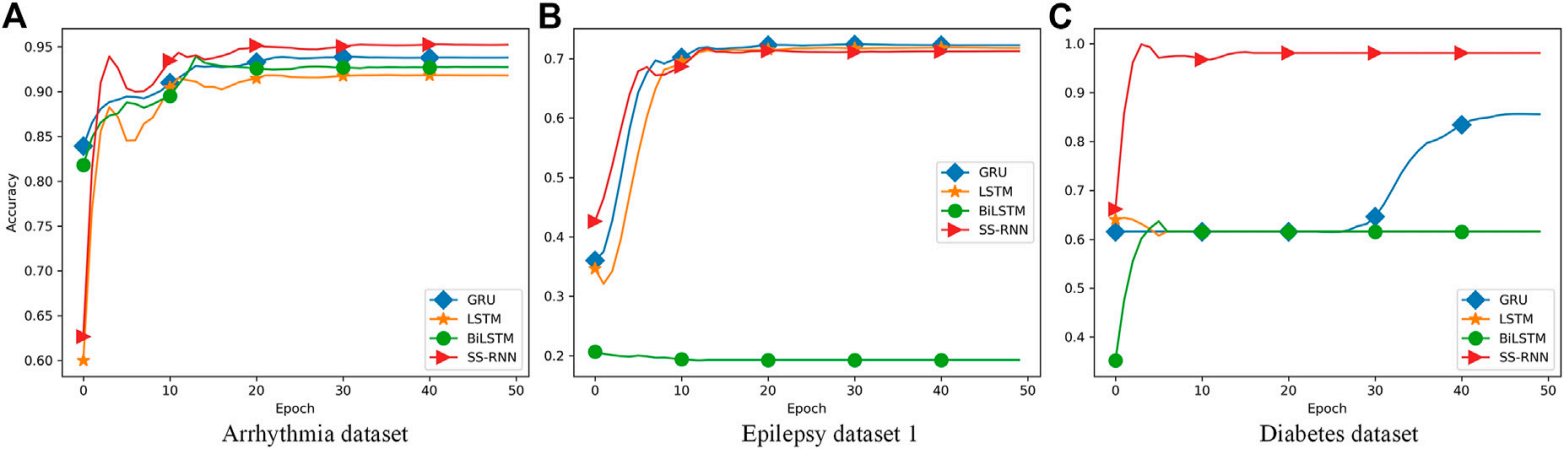
Comparisons between LSTM, GRU, Bi-LSTM, and our SS-RNN (SkipA-3). **(A)** Accuracy of the Arrhythmia dataset. **(B)** Accuracy of the Epilepsy dataset 1. **(C)** Accuracy of the Diabetes dataset.

Furthermore, we also made classifications for three of the datasets (Arrhythmia dataset, Epilepsy dataset 1, and Diabetes dataset) by using the classical networks such as LSTM, GRU, and Bi-LSTM with default parameters of the torch.nn module, and compared the results with that from the SS-RNN with Model A of *skip* = 3 (Figure 6; Tables 2–4). We also show the simulation results of other indexes with Model A of *skip* = 3 and Model C of *skip* = 5 in the Supplementary Material.

**TABLE 2:** Arrhythmia dataset classification comparison results with LSTM, GRU, and Bi-LSTM.

	Accuracy	Precision	Recall	F1-score
LSTM	0.9181	0.9564	0.9380	0.9316
GRU	0.9380	0.9660	0.9380	0.9479
Bi-LSTM	0.9274	0.9596	0.9274	0.9384
SS-RNN(SkipA-3)	0.9524	0.9670	0.9524	0.9573

**TABLE 3:** Epilepsy dataset 1 classification comparison results with LSTM, GRU, and Bi-LSTM.

	Accuracy	Precision	Recall	F1-score
LSTM	0.7178	0.7190	0.7178	0.2506
GRU	0.7226	0.7240	0.7226	0.2540
Bi-LSTM	0.1926	0.0371	0.1926	0.3276
SS-RNN(SkipA-3)	0.7126	0.7115	0.7126	0.3834

**TABLE 4:** Diabetes dataset classification comparison results with LSTM, GRU, and Bi-LSTM.

	Accuracy	Precision	Recall	F1-score
LSTM	0.6154	0.3787	0.6154	0.4689
GRU	0.8556	0.8832	0.8558	0.8467
Bi-LSTM	0.6154	0.3787	0.6154	0.4689
SS-RNN(SkipA-3)	0.9808	0.9817	0.9808	0.9809

From Figure 6, it shows that our SS-RNN method can improve the classification accuracy as compared to the classical methods. Also, from Tables 2–4, it can be found that the other main indexes are almost improved. At the same time, we compared our method with the latest methods RNN, RNN+GRU, RNN+LSTM, and MCNN (Zhang et al., 2017; Singh et al., 2018) with the same Arrhythmia dataset. The result is shown in Table 5, which also indicates that our SS-RNN method can improve the classification accuracy.

**TABLE 5:** Arrhythmia dataset classification comparison results with RNN, RNN+GRU, RNN+LSTM, and MCNN.

	Accuracy	Recall (Sensitivity)
RNN	0.8540	0.8060
RNN GRU	0.8250	0.7890
RNN LSTM	0.8810	0.9240
MCNN	0.9110	NA^a^
SS-RNN(SkipA-3)	0.9524	0.9524

a NA means that it is not available in the original paper.

In fact, as a variant of LSTM, GRU reduces the forget gate and input gate, and adds the update gate. GRU has simpler internal structure and less parameters than LSTM, which reduces the risk of overfitting. Although LSTM and GRU partially solve the problem of the vanishing gradient of the RNN, the information loss is still very severe in the propagation of a very long distance. Bi-LSTM, namely, bi-directional LSTM, does not change any internal structure of LSTM itself. LSTM is applied twice in different directions, and then the LSTM results obtained twice are spliced as the final output. For datasets with both forward and backward dependencies, this method can enhance the correlation between data and improve the efficiency of the model. It is often used to capture some specific pre or post features of language and syntax in natural language processing. However, in biological datasets like ECG and EEG, the progression and onset of diseases are irreversible, so the relationship between data in the reverse time direction is not of practical significance for disease classification. In addition, excessive number of parameters may lead to overfitting in network training, so the Bi-LSTM model is not suitable here.

The long-term memory ability of LSTM and GRU is weak, and with the increase of the time step, the farther away the memory, the more information the model forgot and the less it remembered. Our model has enhanced the information in distant moments, which makes up for the defect of long-term dependence in RNNs. Therefore, our SS-RNN method can improve the precision, recall, F1-score, and finally improve the classification accuracy of sequential data compared with other models.

## Discussion

The performance of the loss function is different between five datasets and six models. Model A has the best performance. In Epilepsy dataset 1, Model A has the lowest loss function and the highest accuracy of all models. In the Diabetes dataset, the loss function of Model A-3 is the lowest and the accuracy is the highest. In the Arrhythmia dataset, the performance of the loss function of each model is different, and Model A has the best performance, in which the loss function is negatively correlated with the order and the accuracy is positively correlated with the order. In Epilepsy dataset 2, overfitting occurred due to the convergence effect of each model. Therefore, Model A did not show good performance, Model C had the lowest loss function, and Model D-2 had the highest accuracy. As for the Breast cancer dataset, the training effect of the network is not optimal because the data scale is too small, and the average loss of Model D-5 is the lowest and the accuracy is the highest. There is a certain relationship between order and accuracy in each model.

**FIGURE 7:**
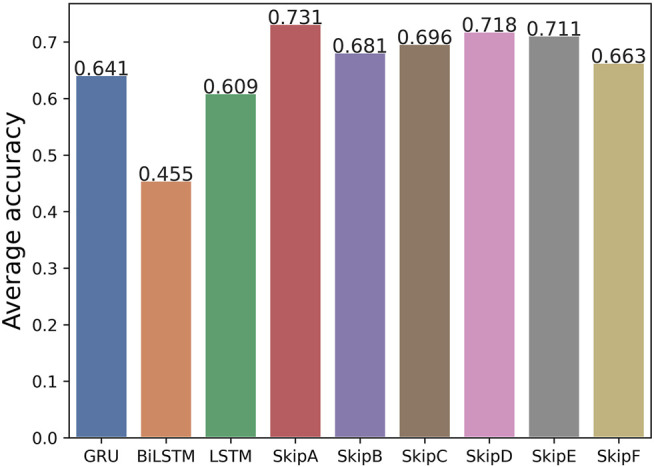
Average accuracy of the Arrhythmia dataset, Epilepsy dataset 1, and Diabetes dataset between the original LSTM, GRU, and Bi-LSTM models and our six models without batch size tuned.

Furthermore, we calculated the average accuracy of the six models by our method on the Arrhythmia dataset, Epilepsy dataset 1, and Diabetes dataset. Comparing the results with the original LSTM, GRU, and Bi-LSTM models, the average accuracy is improved. It is shown in Figure 7. It means that our SS-RNN method is generally useful. We also compared the average accuracy of the six models by our method with original LSTM with five groups of datasets. It can be found in Supplementary Figure S54. According to the results in Figure 7, it shows that Model A is the best with the highest average accuracy among the six ways of adding historical information. By comparing the differences of various adding methodologies, it can be found that the discontinuous adding method is better than the continuous adding method, while the direct adding method is better than the method of weight weighting and function mapping. It does not mean that more historical information is better. Adding more historical information did not improve the memory ability of the RNN. Different data have different dependence intensity, so the same model has different modeling performance for different datasets.

## Conclusion

In order to effectively capture the long-term dependencies in sequential data, we propose the SS-RNN, which allows the historical information to add to the moment by different methods. We designed six models with different *skips* to simulate the possible patterns of the addition of past information, and tested them on five disease-related datasets with different sizes and data types. By comparing our method with the original LSTM, GRU, and Bi-LSTM and the recent methods RNN+GRU, RNN+LSTM, and MCNN, the simulation results suggest that our method can significantly improve the accuracy of sequential data classification. Furthermore, the best method to add the past information could be the method discontinuous addition without weight weighting and function mapping. It can effectively solve the problems of exploding gradient and vanishing gradient. There is a certain correlation between the model performance and the order.

The SS-RNN provides a new idea to improve the classification accuracy of sequential data by optimizing the LSTM model. Therefore, users can also optimize their own network model by adding the SS-RNN module, which is of great significance for the classification diagnosis and precision treatment of diseases. Although the SS-RNN generally has a good classification effect for large datasets, the performance of the model for small sample datasets needs to be further improved. In the future, few-shot learning could be further introduced to train the SS-RNN network to improve the classification efficiency for small sample datasets. The code of the SS-RNN model can be available through github (https://github.com/WTU-RCNS-Bioinformatics-Lab/SS-RNN).

## Data Availability

The original contributions presented in the study are included in the article/Supplementary Material, further inquiries can be directed to the corresponding author.
