# Supplementary material for: SS-RNN: A Strengthened Skip Algorithm for Data Classification Based on Recurrent Neural Networks
Source: Front Genet. 2021 Oct 13;12:746181. doi: 10.3389/fgene.2021.746181 (PMC8548744; doi:10.3389/fgene.2021.746181)
Supplement: Supplementary file 1 [file DataSheet1.pdf]

Supplementary Material

SS-RNN: A strengthened skip algorithm for data classification based on recurrent neural networks

Wenjie Cao[1,2], Ya-Zhou Shi[1], Huahai Qiu[1], Bengong Zhang[1*]

[1] *Research Center of Nonlinear Science, School of Mathematical& Physical Sciences, Wuhan Textile University, Wuhan 430200, China*

[2] *School of Computer Science and Artificial Intelligence, Wuhan Textile University, Wuhan 430200, China*

1 Supplementary Dataset Introduction

MIT-BIH is the Arrhythmia database provided by the Massachusetts Institute of Technology in the United States, it is one of the internationally recognized standard ECG databases and has been widely used in related fields. The dataset contained 48 and a half hours of dual-channel dynamic electrocardiogram recordings of five common different types of heart rates, one of which is normal and the other four are abnormal.

We select two well-known Epilepsy datasets. One is an Epilepsy dataset from the Department of Epilepsy at the University of Bonn, and the other is an Epilepsy dataset from Children's Hospital Boston. The University of Bonn's Epilepsy dataset contains five categories, identified as A, B, C, D and E, each of which contains 100 single-channel 23.6-second segments of EEG signals. The Epilepsy Dataset from Children's Hospital Boston records EEG data from pediatric patients with refractory epileptic seizures, this dataset records EEG data from 22 epileptic patients.

Diabetes mellitus is a group of metabolic diseases characterized by high blood sugar and a common disease with high incidence. Diabetics suffer from the disease for a long time, the current medical level does not have a complete cure for the treatment, and only drugs can be used to maintain blood glucose level. The prevention and diagnosis of Diabetes mellitus is a very meaningful work, which is worthy of further study. The dataset used in the experiment is from UC Irvine Machine Learning Repository, which contains 16 features.

According to the latest data from the 2018 International Agency for Research on Cancer (IARC) survey, the incidence of breast cancer in women worldwide is 24.2%, ranking the first among women's cancers, 52.9% of which occur in developing countries. In China, the incidence of breast cancer is increasing year by year, and more than 300,000 women are diagnosed with breast cancer every year. Breast cancer

is a serious threat to women's life and health, so more attention should be paid to its diagnosis and prevention. The dataset used in the experiment is from UC Irvine Machine Learning Repository, it contains 9 features.

2 Supplementary Formula

The formula S1-S2 is the calculation formula of RNN and GRU respectively.

$$\begin{cases} h_t = f(U \cdot x_t + W \cdot h_{t-1}) \\ O_t = g(V \cdot S_t) \end{cases} \tag{S1}$$

$$\begin{cases} r_t = \sigma(W_{rh} h_{t-1} + W_{rx} x_t + b_r) \\ z_t = \sigma(W_{zh} h_{t-1} + W_{zx} x_t + b_z) \\ \tilde{h}_t = \tanh(W_{\tilde{h}h}(r_t \cdot h_{t-1}) + W_{\tilde{h}x} x_t + b_z) \\ h_t = (1 - z_t) \cdot h_{t-1} + z_t \cdot \tilde{h}_t \end{cases} \tag{S2}$$

The formula S3-S4 is the improved formula of RNN and GRU of Model A.

$$\begin{cases} \boldsymbol{M = f(U \cdot x_t + W \cdot h_{t-1})} \\ \boldsymbol{h_t} = \begin{cases} \boldsymbol{M + h_{t-skip}}, & \boldsymbol{if\ t = 1 + i \times skip} \\ \boldsymbol{M}, & \boldsymbol{if\ t \neq 1 + i \times skip} \end{cases} \\ O_t = g(V \cdot h_t) \end{cases} \tag{S3}$$

$$\begin{cases} f_t = \sigma\left(W_{fh} h_{t-1} + W_{fx} x_t + b_f\right) \\ \tilde{h}_t = tanh(\,W_{\tilde{h}h}(f_t \cdot h_{t-1} + W_{\tilde{h}x} x_t + b_{\tilde{h}})) \\ \boldsymbol{Q = (1 - z_t) \cdot h_{t-1} + z_t \cdot \tilde{h}_t} \\ \boldsymbol{h_t} = \begin{cases} \boldsymbol{Q + h_{t-skip}}, & \boldsymbol{if\ t = 1 + i \times skip} \\ \boldsymbol{Q}, & \boldsymbol{if\ t \neq 1 + i \times skip} \end{cases} \end{cases} \tag{S4}$$

The formula S5-S6 is the improved formula of RNN and GRU of Model B.

$$\begin{cases} M = W \cdot h_{t-1} + \boldsymbol{W_{skip} \cdot h_{t-skip}} \\ h_t = \begin{cases} f\left(U \cdot x_t + b_f + \boldsymbol{M}\right), & \boldsymbol{if\ t = 1 + i \times skip} \\ f\left(U \cdot x_t + W \cdot h_{t-1} + b_f\right), & \boldsymbol{if\ t \neq 1 + i \times skip} \end{cases} \\ O_t = g(V \cdot h_t) \end{cases} \tag{S5}$$

$$\begin{cases} M = W_{rh} h_{t-1} + \boldsymbol{W_{rskip} \cdot h_{t-skip}} \\ r_t = \begin{cases} \sigma(W_{rx} x_t + b_r + M), & \boldsymbol{if\ t = 1 + i \times skip} \\ \sigma(W_{rh} h_{t-1} + W_{rx} x_t + b_r), & \boldsymbol{if\ t \neq 1 + i \times skip} \end{cases} \\ M = W_{zh} h_{t-1} + \boldsymbol{W_{zskip} \cdot h_{t-skip}} \\ z_t = \begin{cases} \sigma(W_{zx} x_t + b_z + M), & \boldsymbol{if\ t = 1 + i \times skip} \\ \sigma(W_{zh} h_{t-1} + W_{zx} x_t + b_z), & \boldsymbol{if\ t \neq 1 + i \times skip} \end{cases} \\ Q = W_{\tilde{h}h}(r_t \cdot h_{t-1}) + \boldsymbol{W_{\tilde{h}skip}}\left(r_t \cdot \boldsymbol{h_{t-skip}}\right) \\ \tilde{h}_t = \begin{cases} tanh(\,W_{\tilde{h}x} x_t + b_z), & \boldsymbol{if\ t = 1 + i \times skip} \\ tanh(\,W_{\tilde{h}h}(r_t \cdot h_{t-1}) + W_{\tilde{h}x} x_t + b_z), & \boldsymbol{if\ t \neq 1 + i \times skip} \end{cases} \\ h_t = (1 - z_t) \cdot h_{t-1} + z_t \cdot \tilde{h}_t \end{cases} \tag{S6}$$

The formula S7-S8 is the improved formula of RNN and GRU of Model C.

$$\begin{cases} h_t = f(U \cdot x_t + W \cdot h_{t-1}) + \boldsymbol{h_{t-skip}} \\ O_t = g(V \cdot h_t) \end{cases} \tag{S7}$$

$$\begin{cases} r_t = \sigma(W_{rh}h_{t-1} + W_{rx}x_t + b_r) \\ z_t = \sigma(W_{zh}h_{t-1} + W_{zx}x_t + b_z) \\ \tilde{h}_t = tanh(W_{\tilde{h}h}(r_t \cdot h_{t-1}) + W_{\tilde{h}x}x_t + b_z) \\ h_t = (1 - z_t) \cdot h_{t-1} + z_t \cdot \tilde{h}_t + \boldsymbol{h_{t-skip}} \end{cases} \tag{S8}$$

The formula S9-S10 is the improved formula of RNN and GRU of Model D.

$$\begin{cases} h_t = f\left(U \cdot x_t + W \cdot h_{t-1} + \boldsymbol{W_{skip}} \cdot \boldsymbol{h_{t-skip}} + b_f\right) \\ O_t = g(V \cdot h_t) \end{cases} \tag{S9}$$

$$\begin{cases} r_t = \sigma\left(W_{rh}h_{t-1} + W_{rx}x_t + \boldsymbol{W_{rskip}} \cdot \boldsymbol{h_{t-skip}} + b_r\right) \\ z_t = \sigma\left(W_{zh}h_{t-1} + W_{zx}x_t + \boldsymbol{W_{zskip}} \cdot \boldsymbol{h_{t-skip}} + b_z\right) \\ \tilde{h}_t = tanh(W_{\tilde{h}h}(r_t \cdot h_{t-1}) + W_{\tilde{h}x}x_t + \boldsymbol{W_{\tilde{h}skip}}(r_t \cdot \boldsymbol{h_{t-skip}}) + b_z) \\ h_t = (1 - z_t) \cdot h_{t-1} + z_t \cdot \tilde{h}_t \end{cases} \tag{S10}$$

The formula S11-S12 is the improved formula of RNN and GRU of Model E.

$$\begin{cases} \boldsymbol{M} = \boldsymbol{W_1 h_{t-1}} + \boldsymbol{W_2 h_{t-2}} + \boldsymbol{W_3 h_{t-3}} \\ h_t = f\left(U \cdot x_t + b_f + \boldsymbol{M}\right) \\ O_t = g(V \cdot h_t) \end{cases} \tag{S11}$$

$$\begin{cases} \boldsymbol{M} = \boldsymbol{W_{r1} h_{t-1}} + \boldsymbol{W_{r2} h_{t-2}} + \boldsymbol{W_{r3} h_{t-3}} \\ r_t = \sigma(W_{rx}x_t + b_r + \boldsymbol{M}) \\ \boldsymbol{N} = \boldsymbol{W_{z1} h_{t-1}} + \boldsymbol{W_{z2} h_{t-2}} + \boldsymbol{W_{z3} h_{t-3}} \\ z_t = \sigma(W_{zx}x_t + b_z + \boldsymbol{N}) \\ \boldsymbol{Q} = \boldsymbol{W_{\tilde{h}1}}(\boldsymbol{r_t} \cdot \boldsymbol{h_{t-1}}) + \boldsymbol{W_{\tilde{h}2}}(\boldsymbol{r_t} \cdot \boldsymbol{h_{t-2}}) + \boldsymbol{W_{\tilde{h}3}}(\boldsymbol{r_t} \cdot \boldsymbol{h_{t-3}}) \\ \tilde{h}_t = tanh(W_{\tilde{h}x}x_t + b_z + \boldsymbol{Q}) \\ h_t = (1 - z_t) \cdot h_{t-1} + z_t \cdot \tilde{h}_t \end{cases} \tag{S12}$$

The formula S13-S14 is the improved formula of RNN and GRU of Model F.

$$\begin{cases} \boldsymbol{M} = \boldsymbol{f}(\boldsymbol{U} \cdot \boldsymbol{x_t} + \boldsymbol{W} \cdot \boldsymbol{h_{t-1}}) \\ \boldsymbol{h_t} = \begin{cases} \boldsymbol{M} + \sum_{s=2}^{skip} \boldsymbol{h_{t-s}}, & \boldsymbol{if\ t = 1 + i \times skip} \\ \boldsymbol{M}, & \boldsymbol{if\ t \neq 1 + i \times skip} \end{cases} \\ O_t = g(V \cdot h_t) \end{cases} \tag{S13}$$

$$\begin{cases} f_t = \sigma\left(W_{fh}h_{t-1} + W_{fx}x_t + b_f\right) \\ \tilde{h}_t = tanh(\,W_{\tilde{h}h}(f_t \cdot h_{t-1} + W_{\tilde{h}x}x_t + b_{\tilde{h}})) \\ \boldsymbol{Q} = (\boldsymbol{1} - \boldsymbol{z_t}) \cdot \boldsymbol{h_{t-1}} + \boldsymbol{z_t} \cdot \boldsymbol{\tilde{h}_t} \\ \boldsymbol{h_t} = \begin{cases} \boldsymbol{Q} + \sum_{s=2}^{skip} \boldsymbol{h_{t-s}}\,, & \boldsymbol{if\ t = 1 + i \times skip} \\ \boldsymbol{Q}, & \boldsymbol{if\ t \neq 1 + i \times skip} \end{cases} \end{cases} \quad (S14)$$

3 Supplementary Figures and Tables

3.1 Supplementary Tables

Table S1. Characteristics of the Diabetes dataset

Age	Gender	Polyuria	Polydipsia
sudden weight loss	weakness	Polyphagia	Genital thrush
visual blurring	Itching	Irritability	delayed healing
partial paresis	muscle stiffness	Alopecia	Obesity

Table S2. Features of the Breast cancer dataset

Age	BMI	Glucose
Insulin	HOMA	Leptin
Adiponectin	Resistin	MCP1

Table S3. Fine tuning of Batchsize

Dataset	Batchsize (before tuning)	Batchsize (after tuning)
Arrhythmia dataset	2000	100
Epilepsy dataset 1	1000	50
Epilepsy dataset 2	5000	5000
Diabetes dataset	20	20
Breast cancer dataset	20	20

3.2 Supplementary Figures

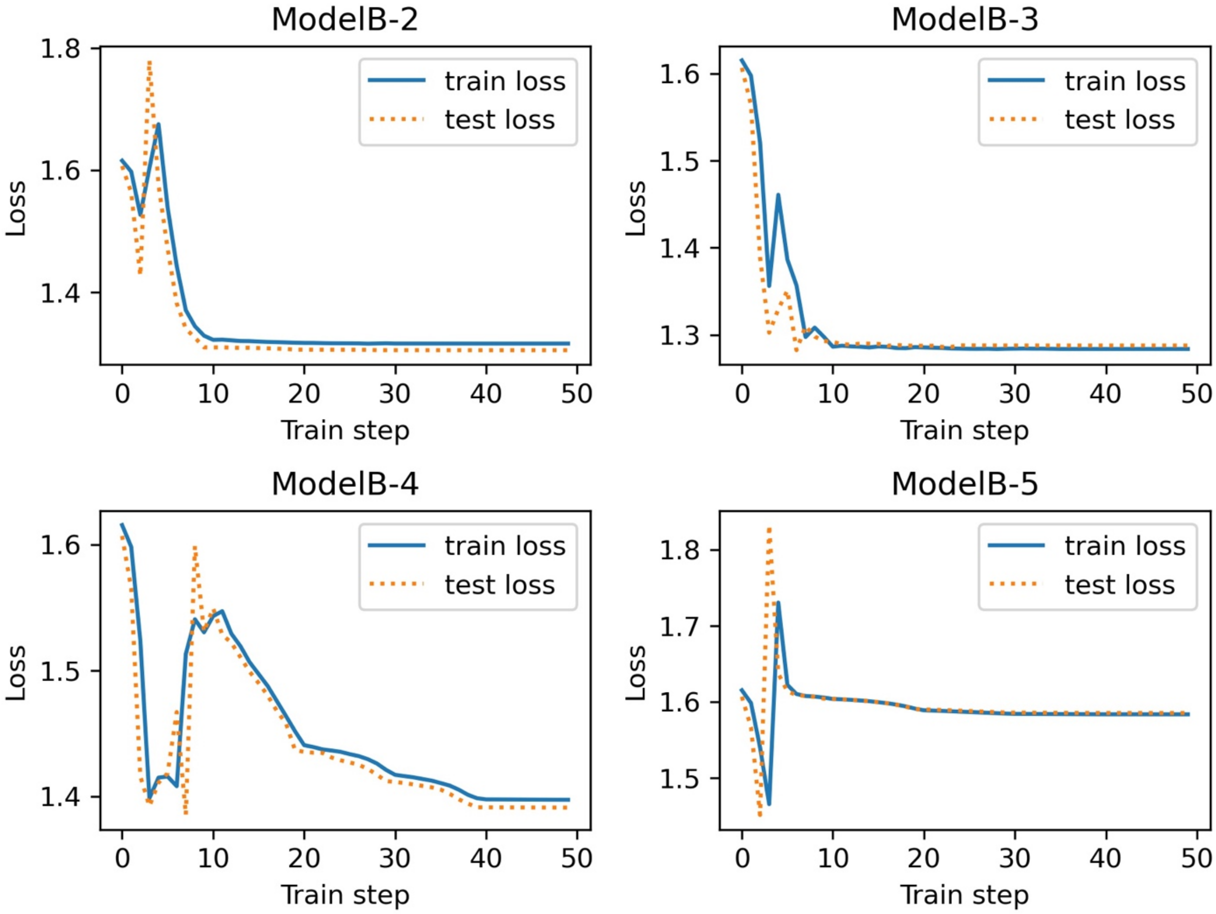

Supplementary Figure 1. Model B curve of changes during training on Epilepsy dataset 1.

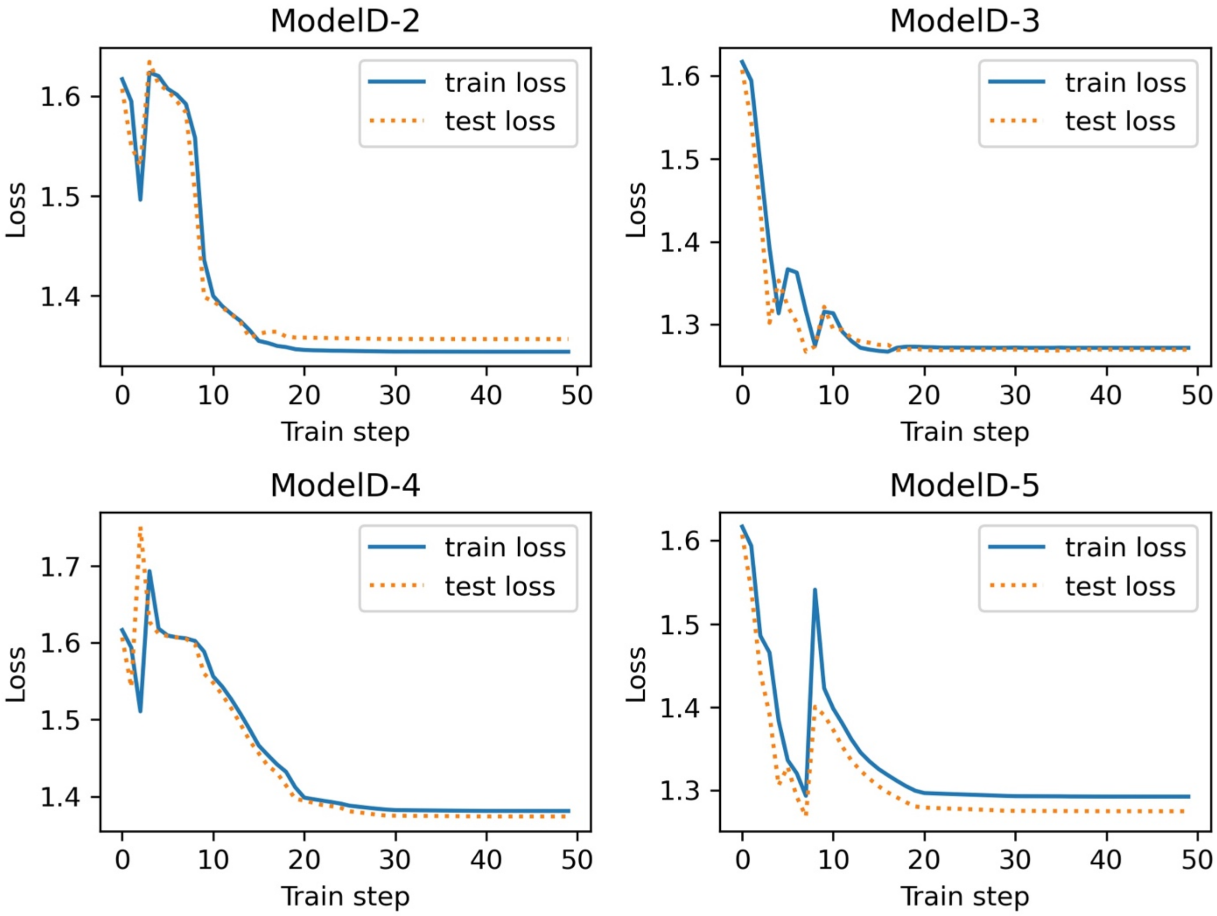

Supplementary Figure 2. Model D curve of changes during training on Epilepsy dataset 1.

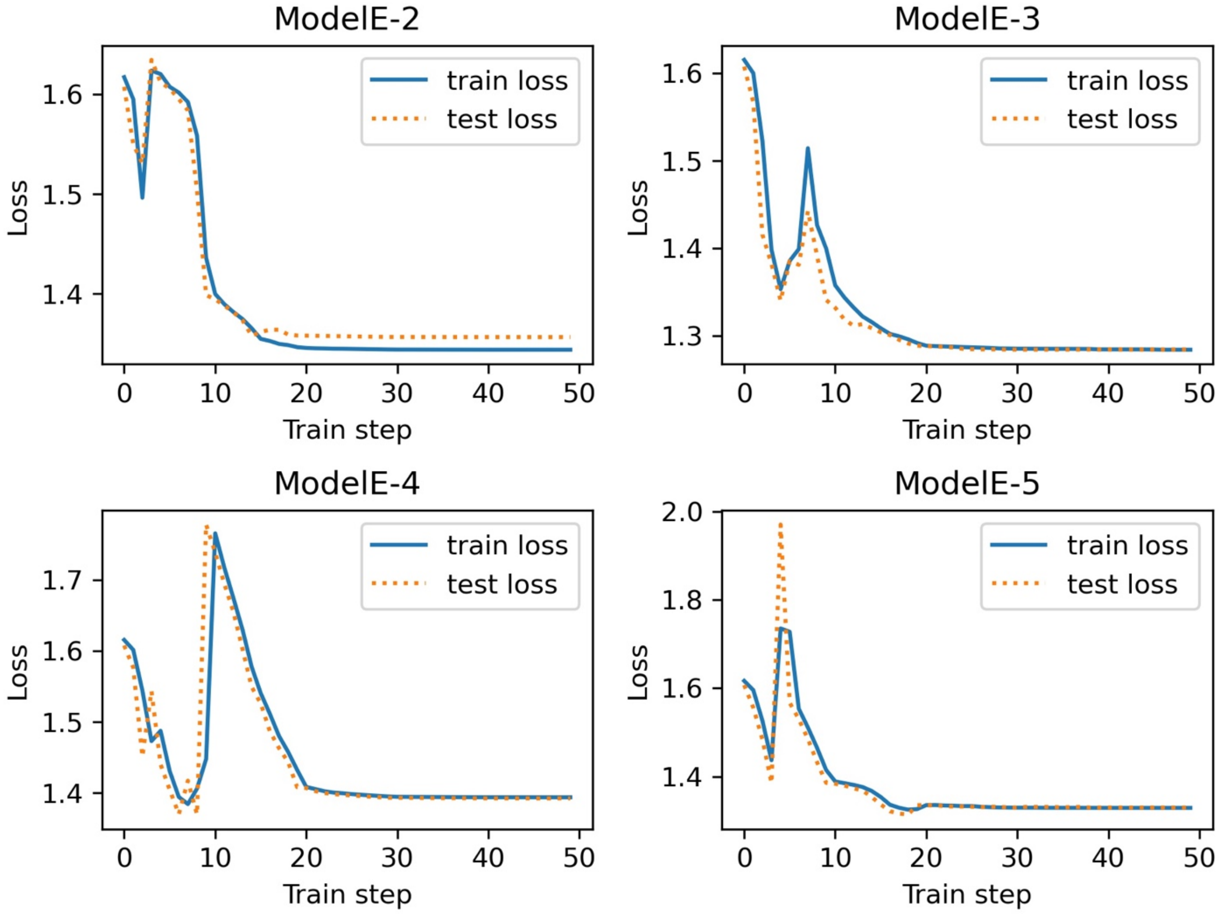

Supplementary Figure 3. Model E curve of changes during training on Epilepsy dataset 1.

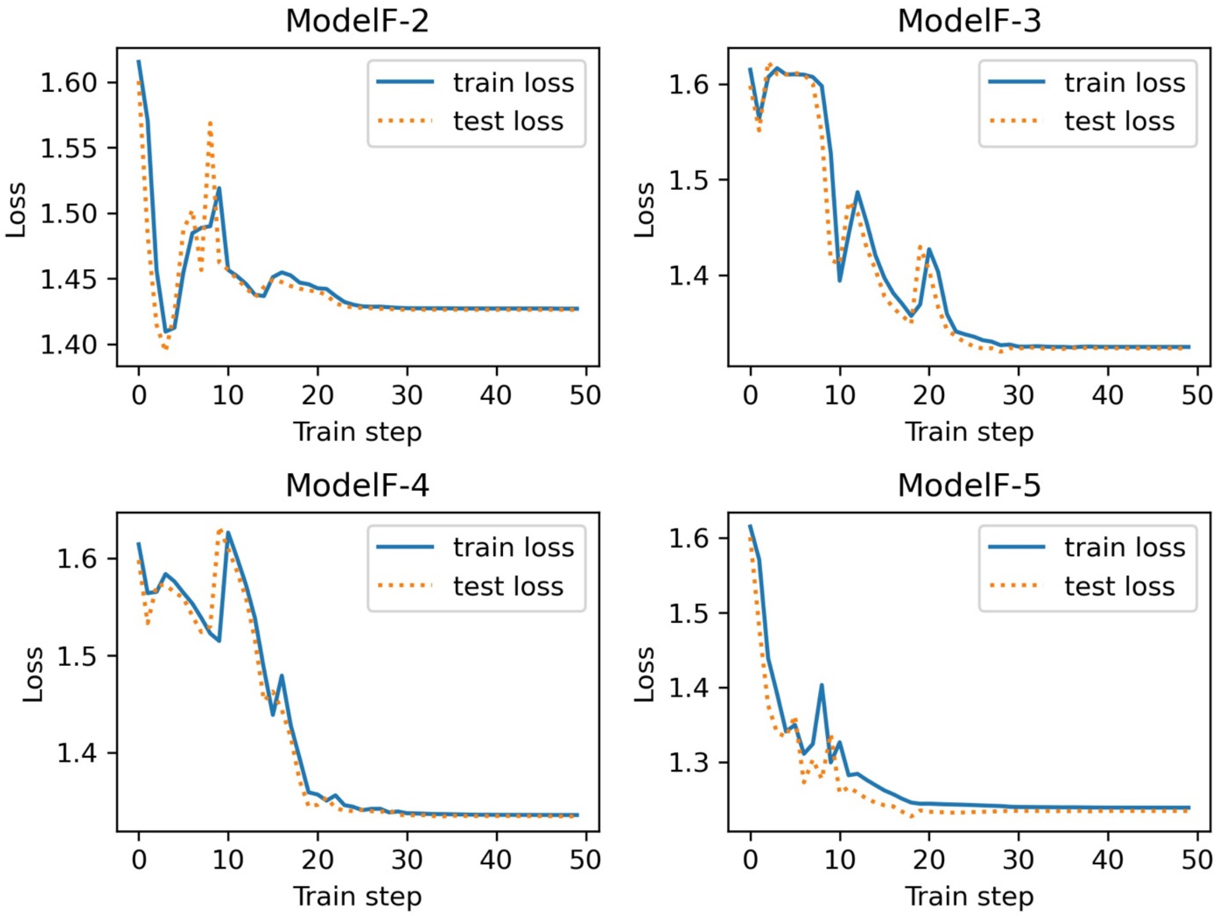

Supplementary Figure 4. Model F curve of changes during training on Epilepsy dataset 1.

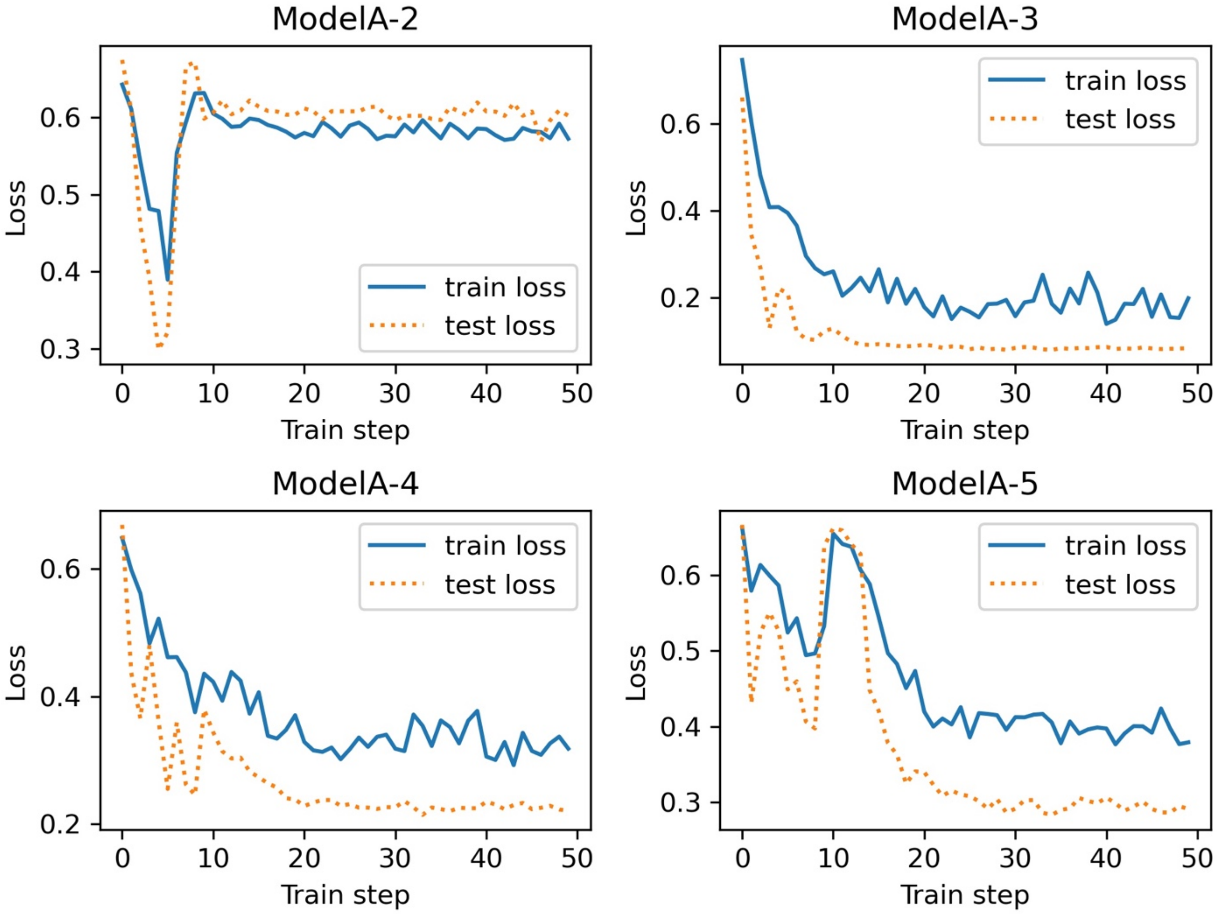

Supplementary Figure 5. Model A curve of changes during training on Diabetes dataset.

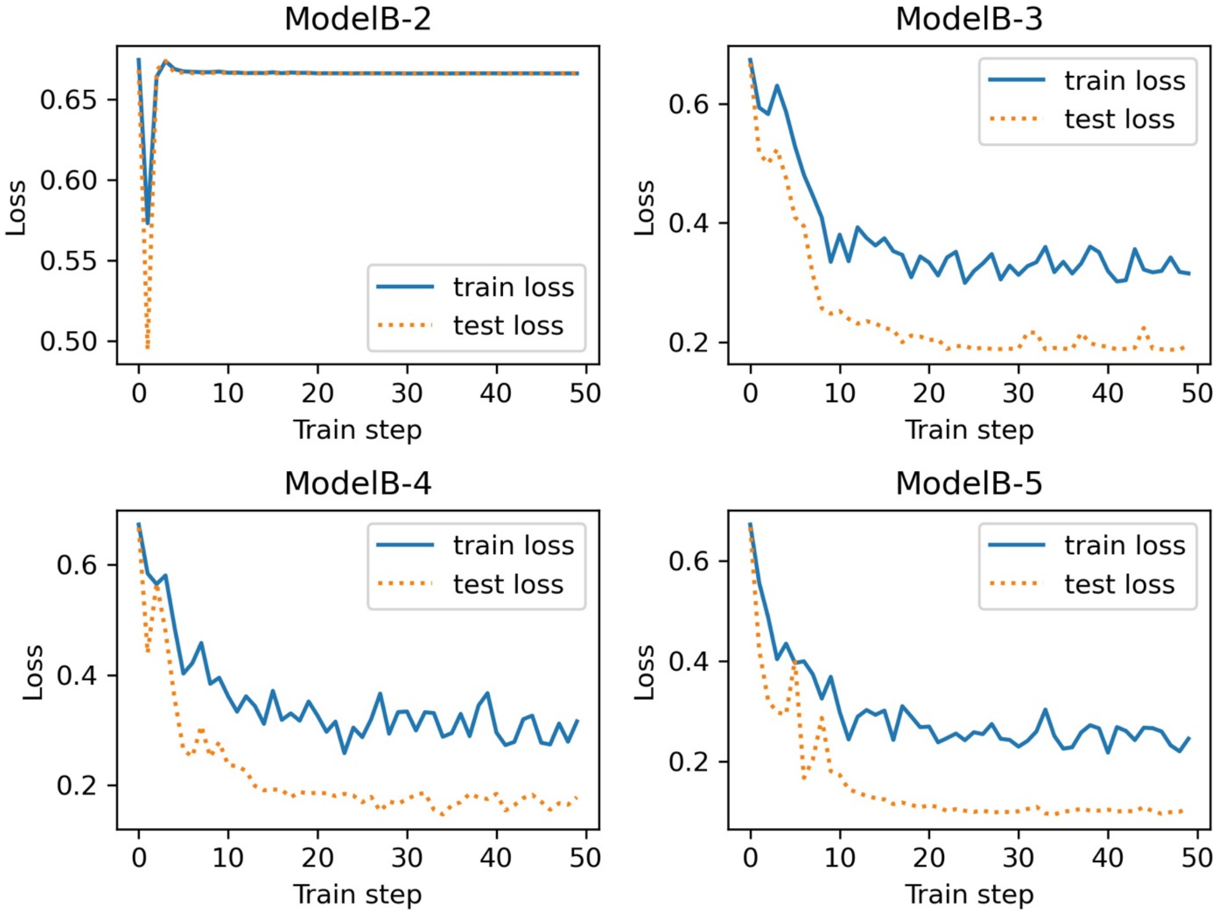

Supplementary Figure 6. Model B curve of changes during training on Diabetes dataset.

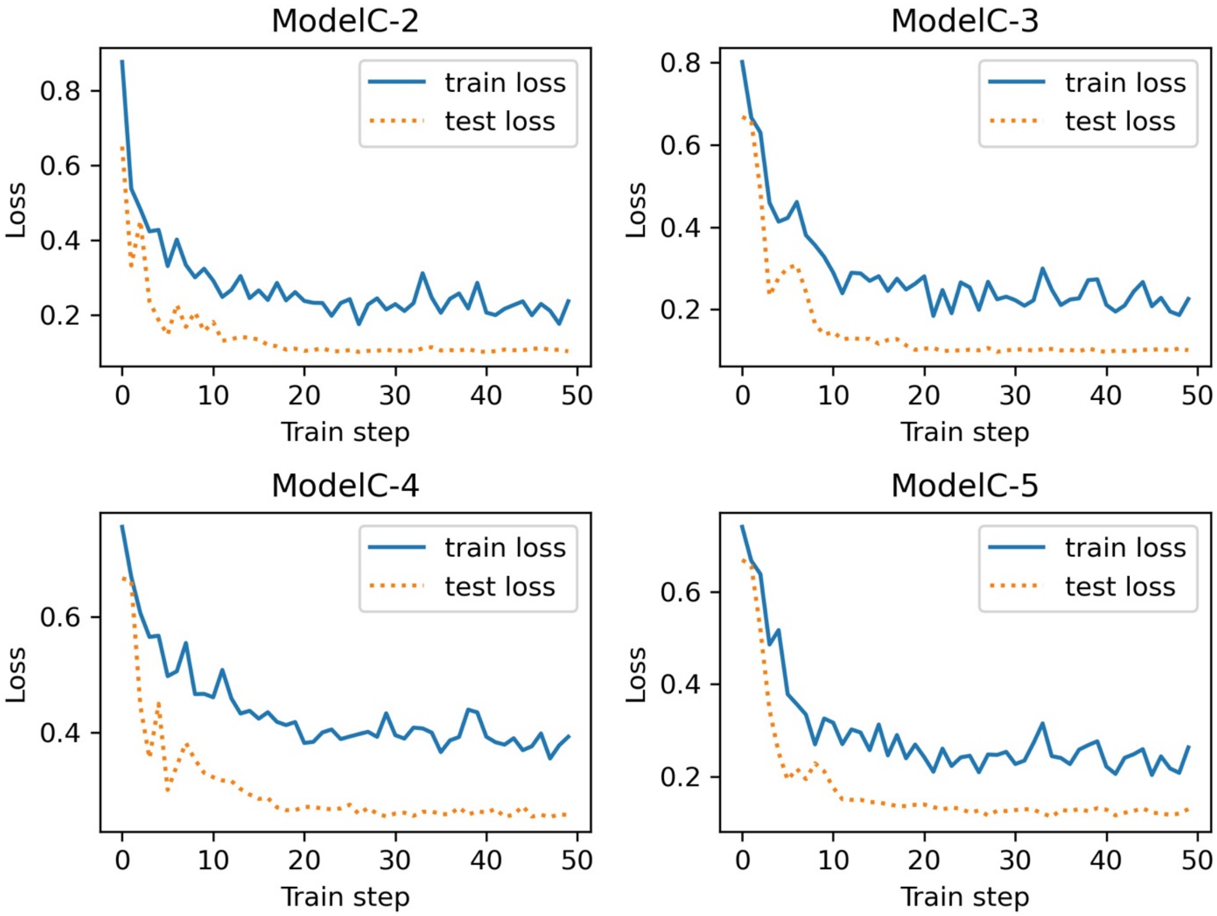

Supplementary Figure 7. Model C curve of changes during training on Diabetes dataset.

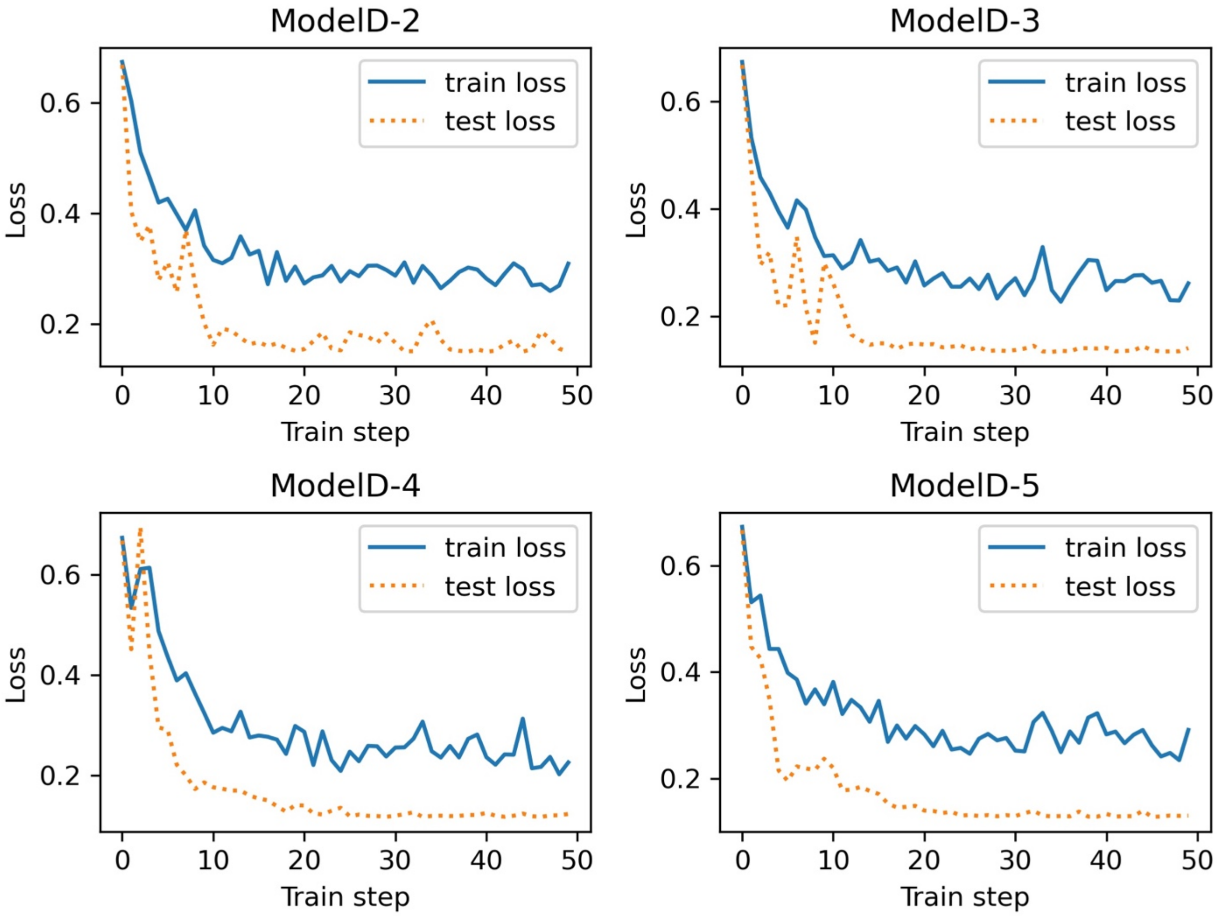

Supplementary Figure 8. Model D curve of changes during training on Diabetes dataset.

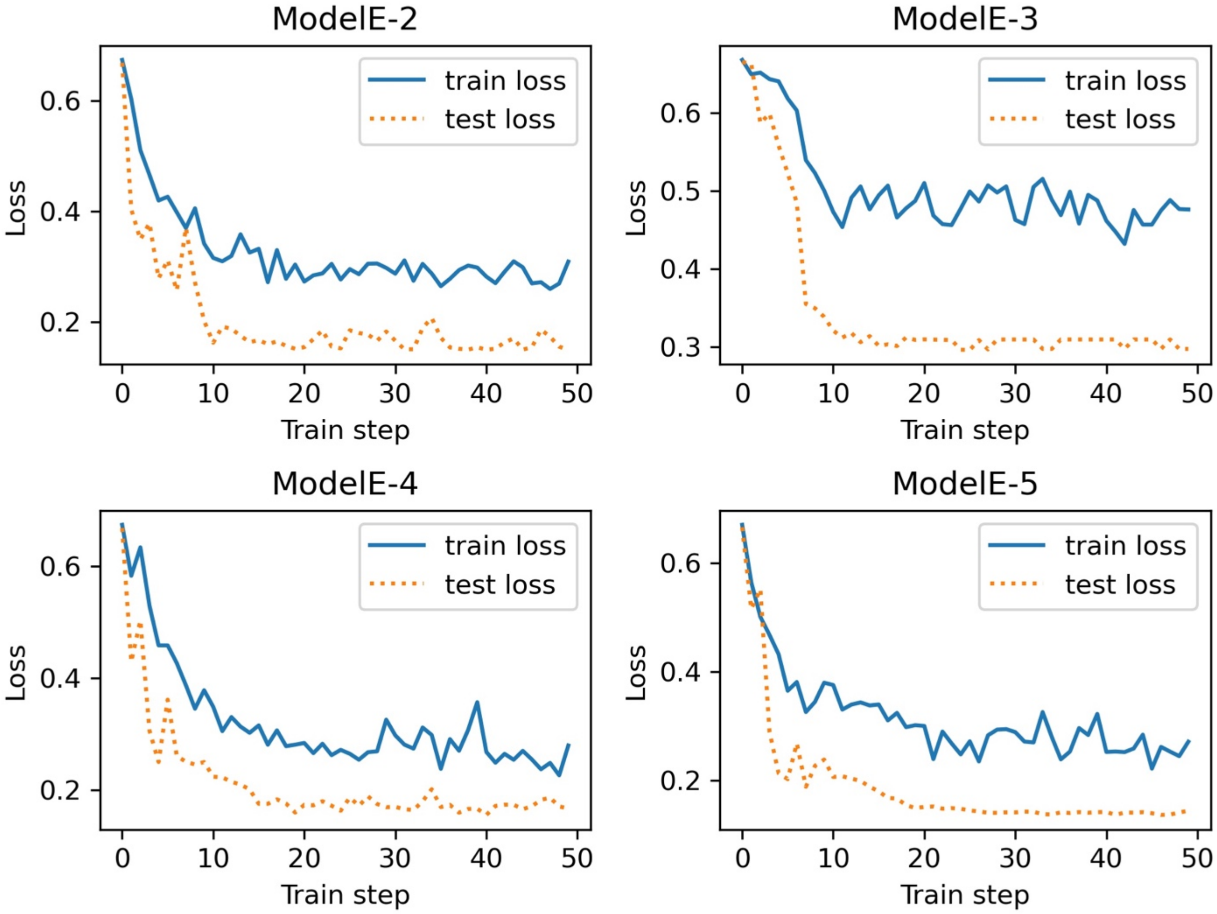

Supplementary Figure 9. Model E curve of changes during training on Diabetes dataset.

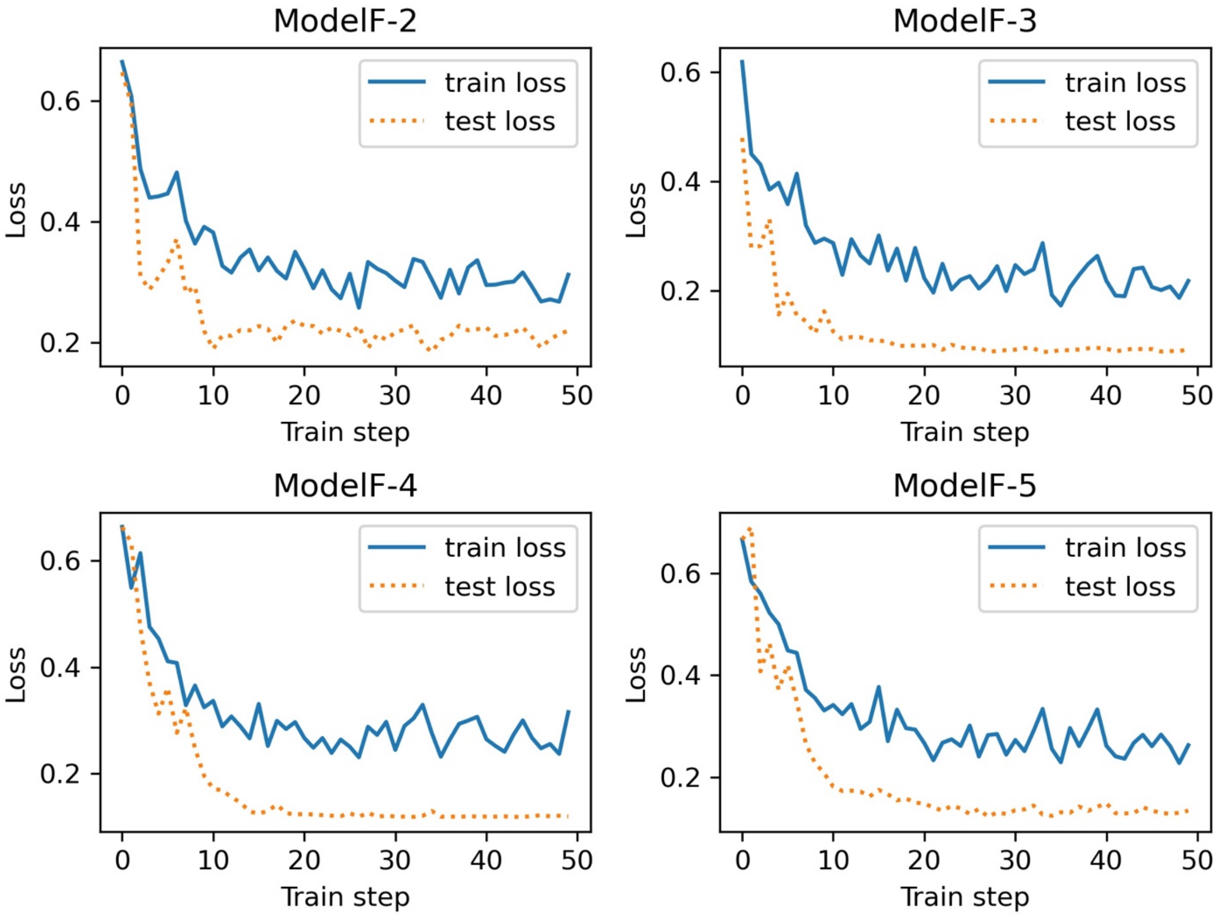

Supplementary Figure 10. Model F curve of changes during training on Diabetes dataset.

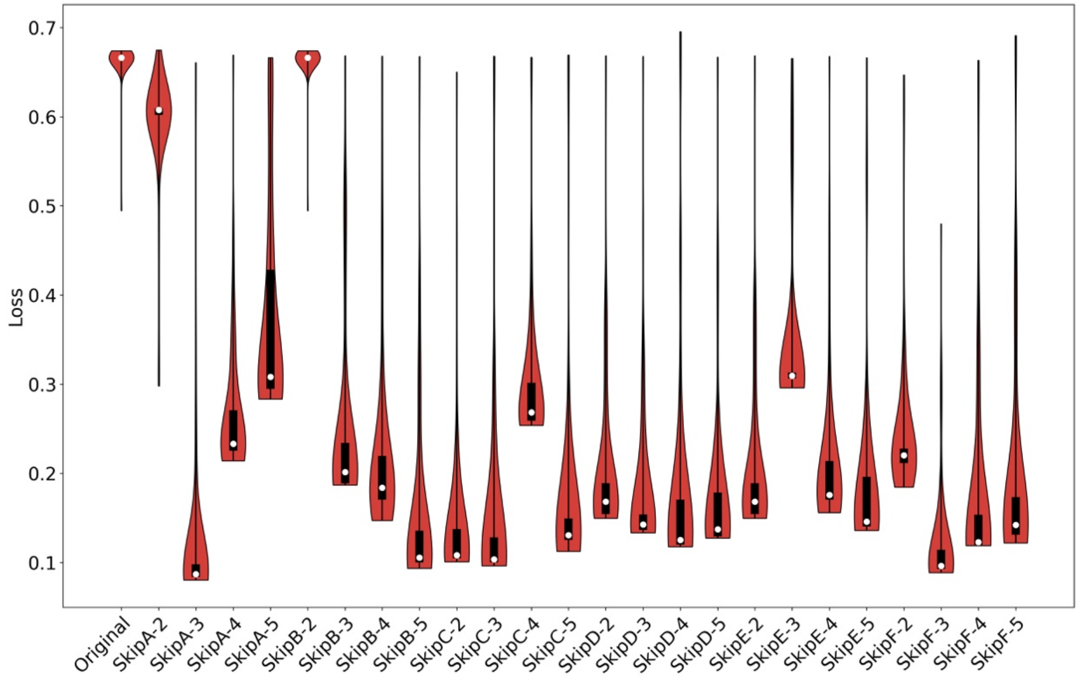

Supplementary Figure 11. Loss of classification of each model for Diabetes dataset.

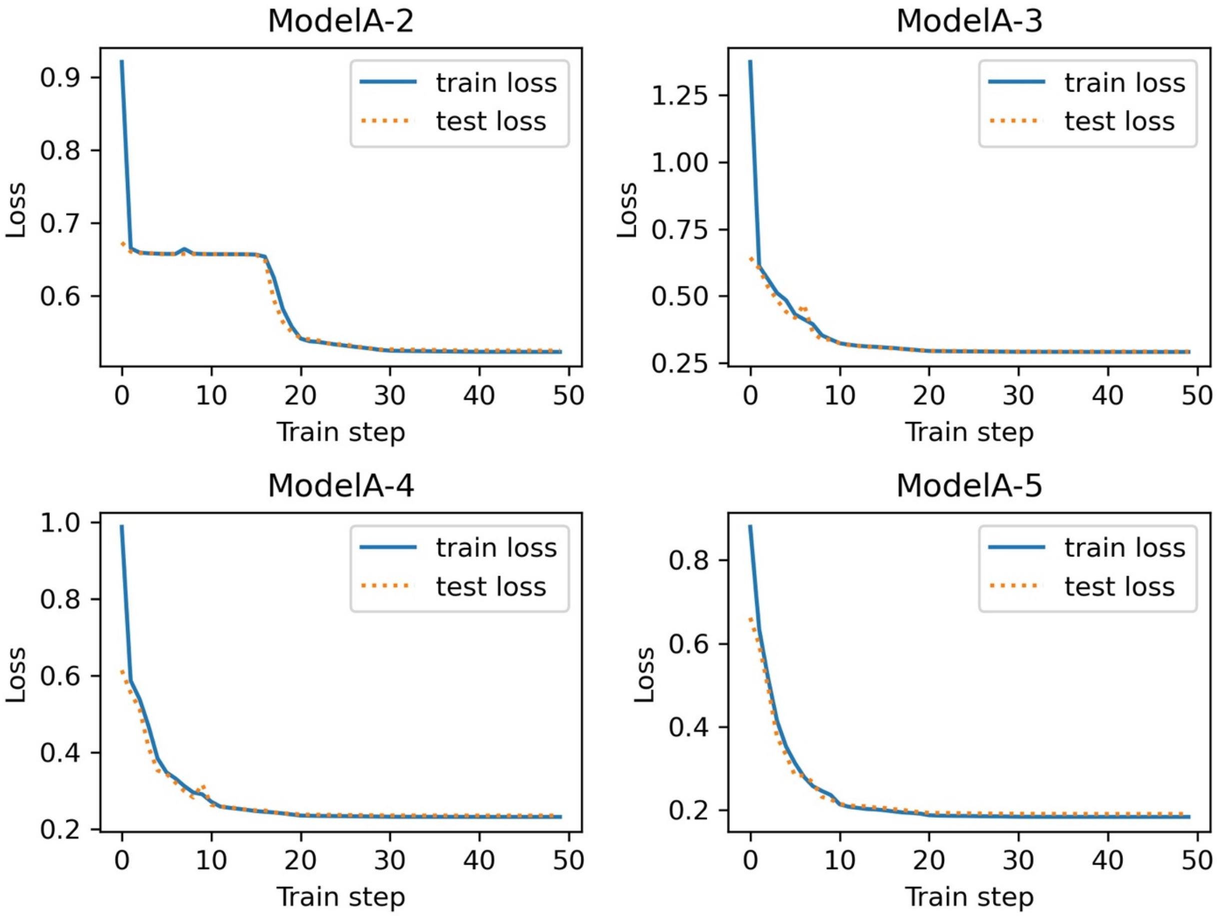

Supplementary Figure 12. Model A curve of changes during training on Arrhythmia dataset.

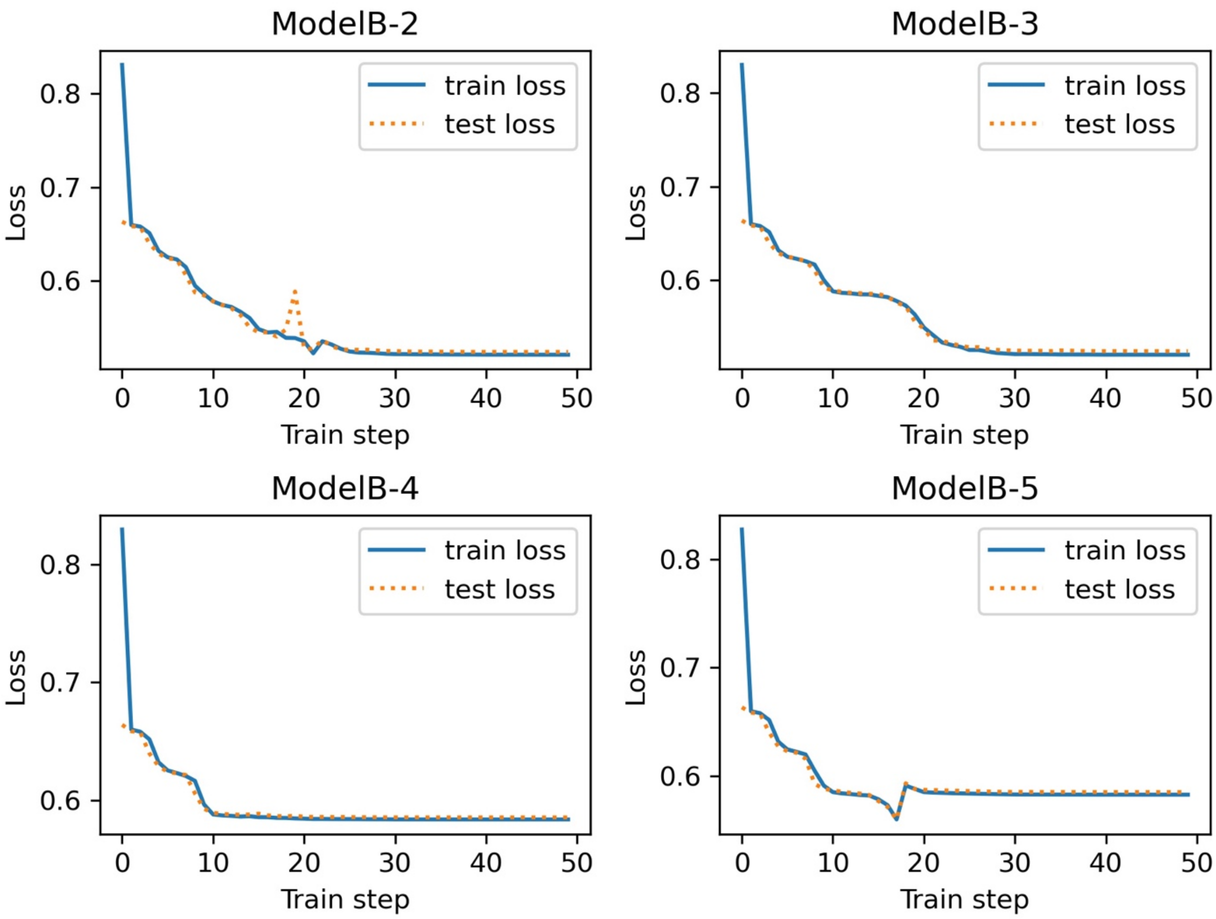

Supplementary Figure 13. Model B curve of changes during training on Arrhythmia dataset.

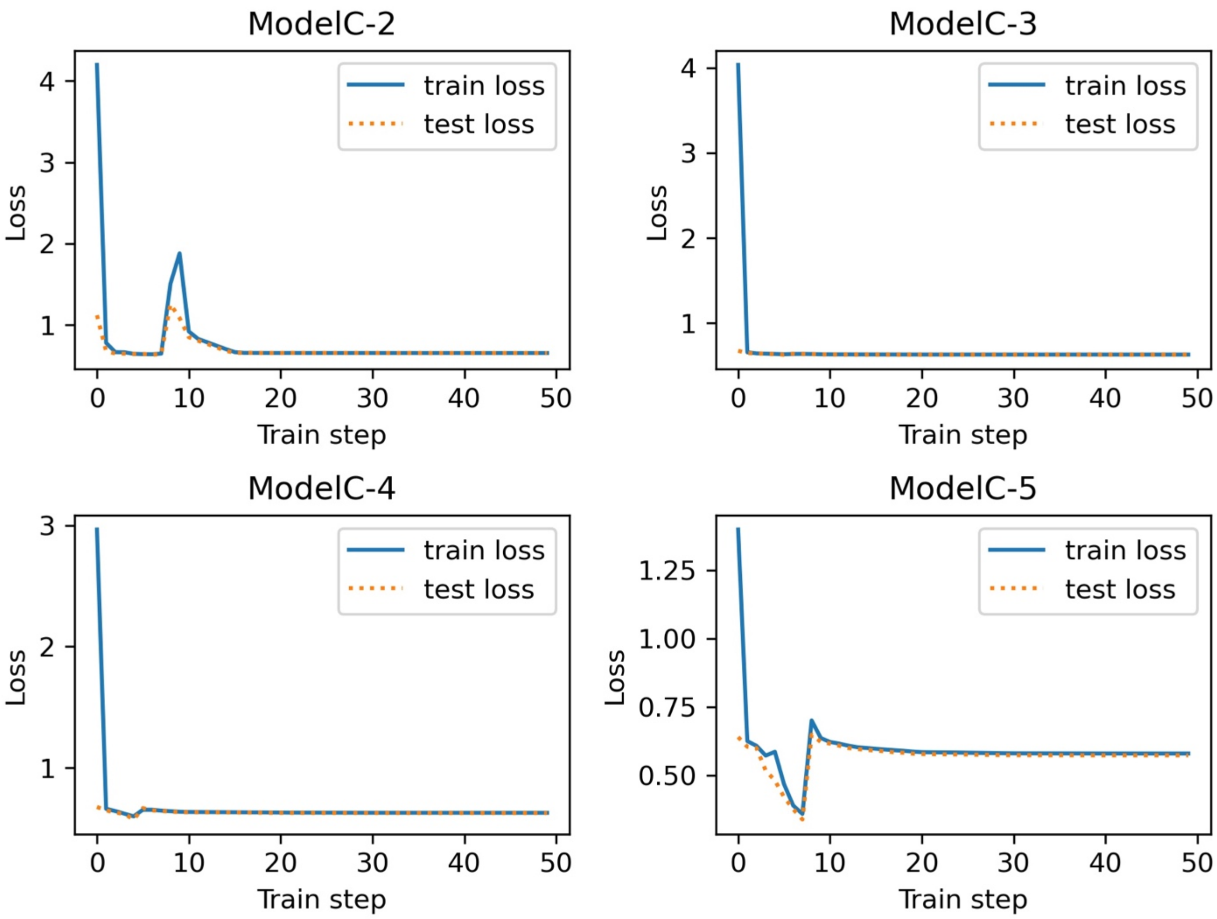

Supplementary Figure 14. Model C curve of changes during training on Arrhythmia dataset.

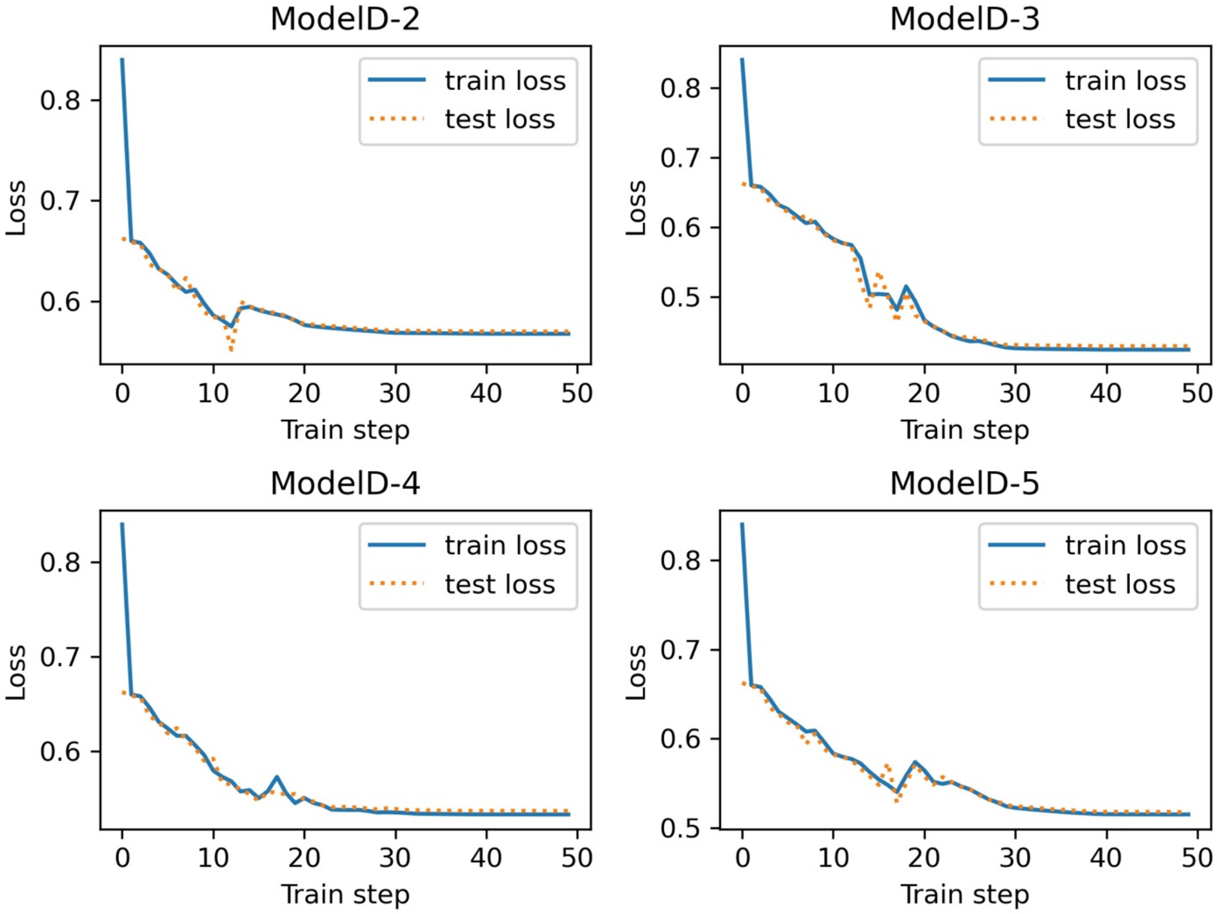

Supplementary Figure 15. Model D curve of changes during training on Arrhythmia dataset.

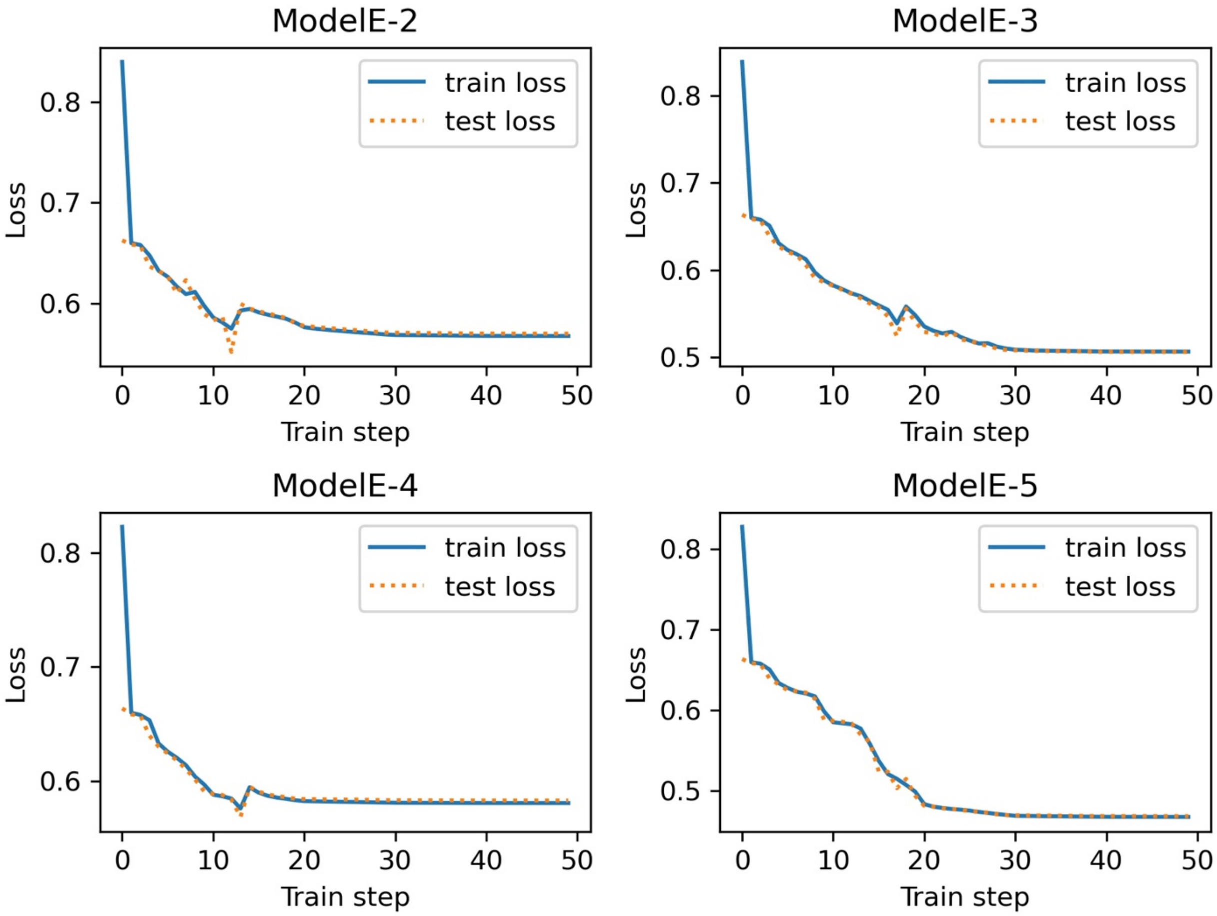

Supplementary Figure 16. Model E curve of changes during training on Arrhythmia dataset.

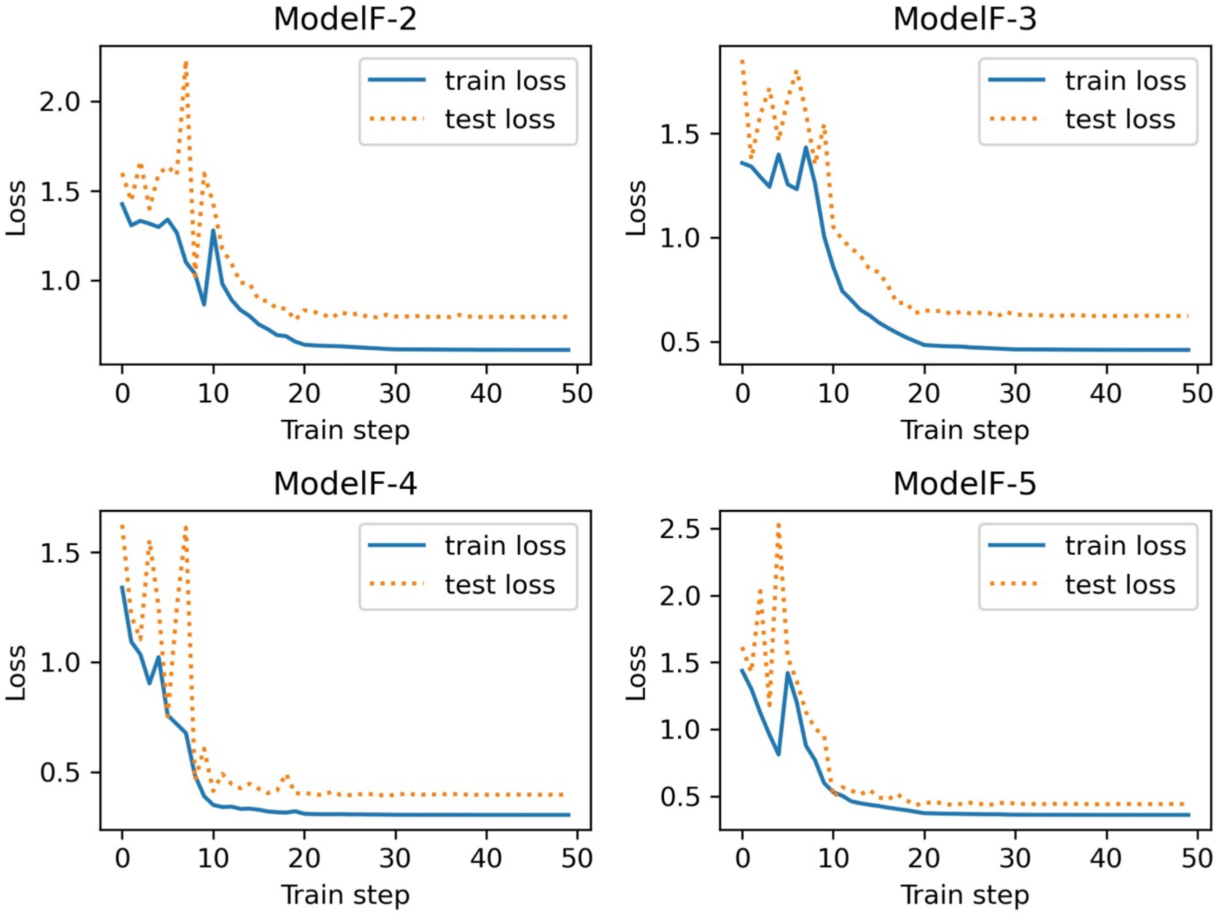

Supplementary Figure 17. Model F curve of changes during training on Arrhythmia dataset.

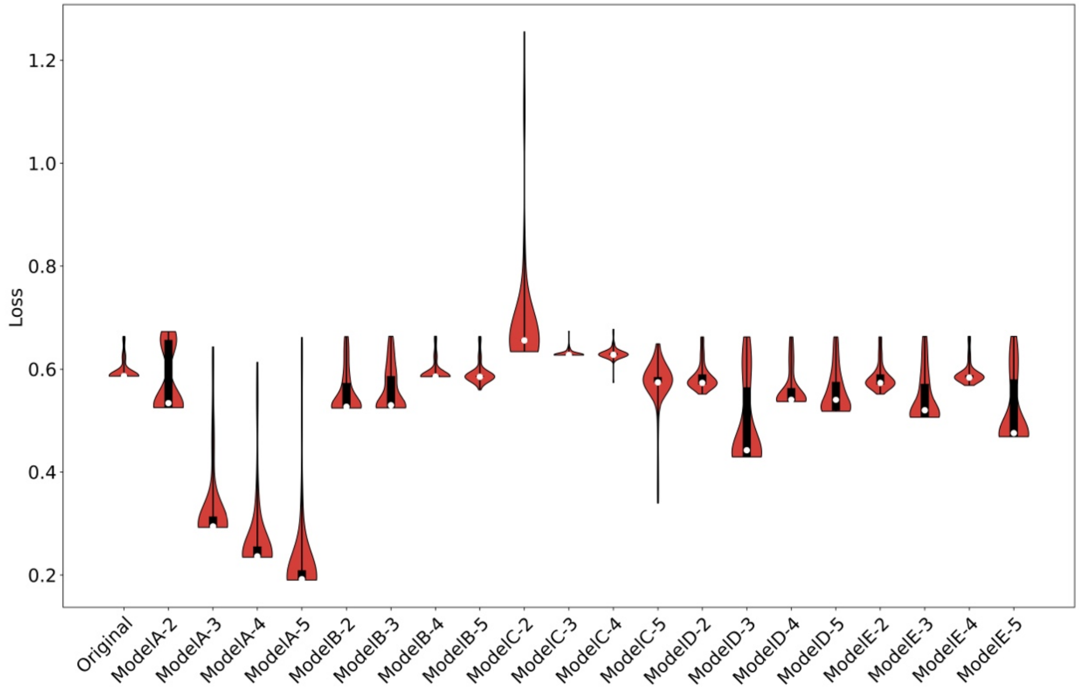

Supplementary Figure 18. Loss of classification of each model for Arrhythmia dataset.

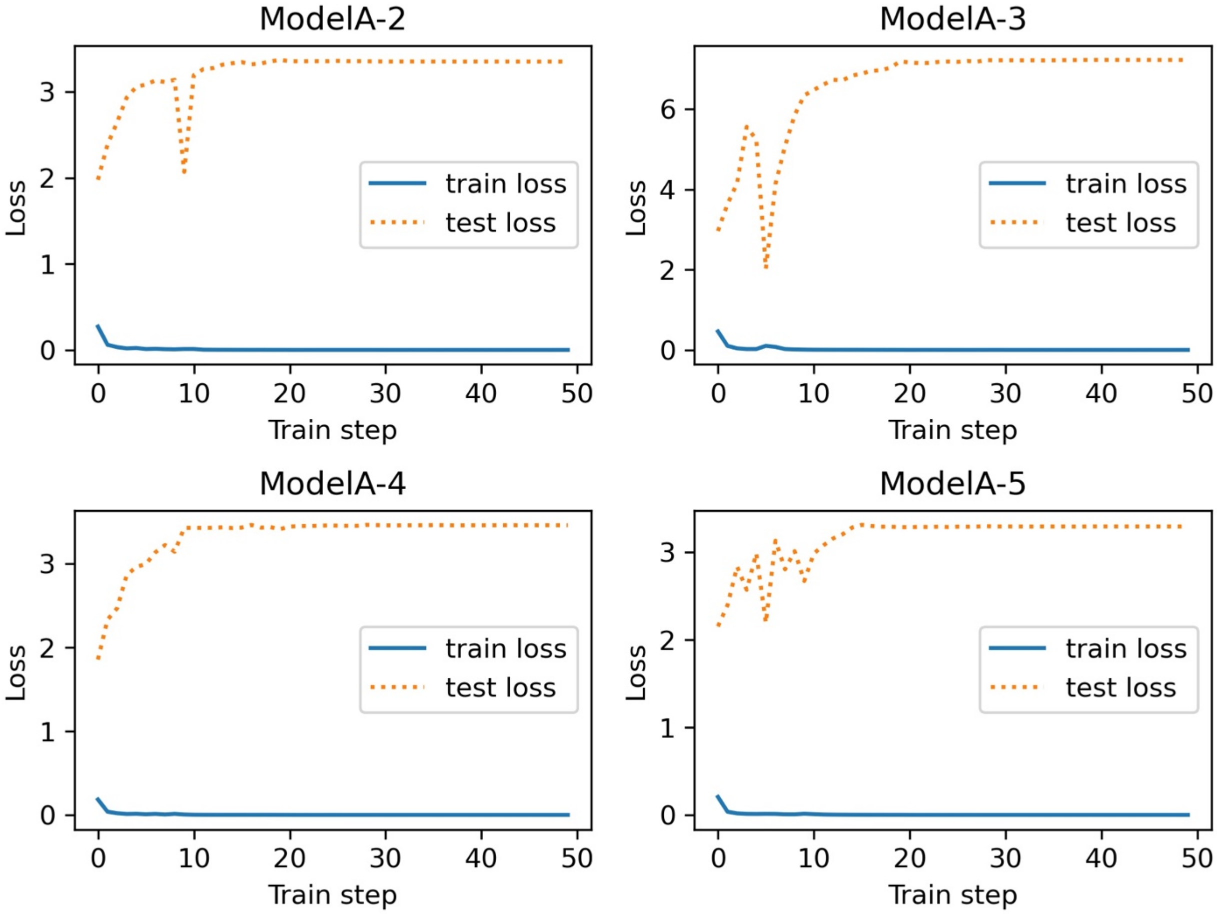

Supplementary Figure 19. Model A curve of changes during training on Epilepsy dataset 2.

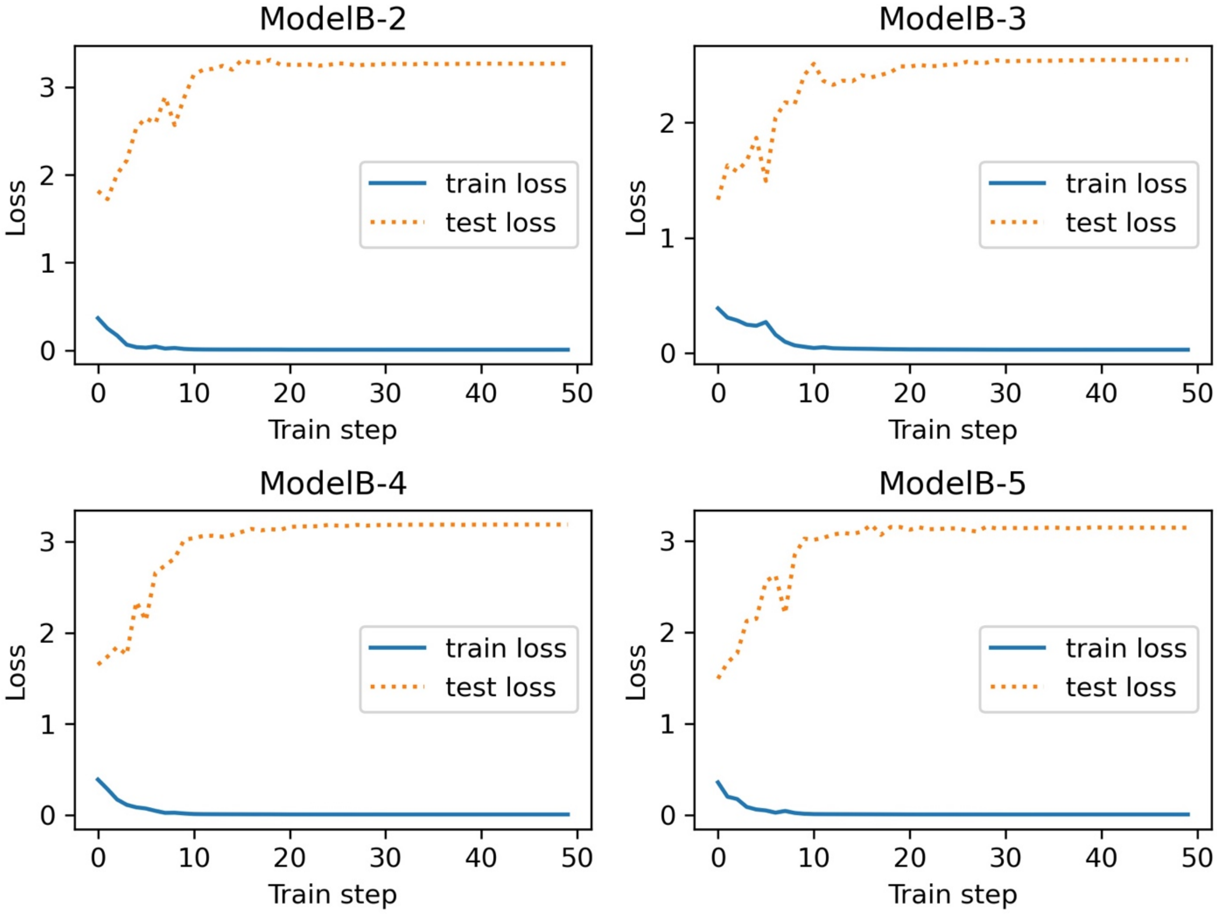

1. **Supplementary Figure 20.** Model B curve of changes during training on Epilepsy dataset 2.

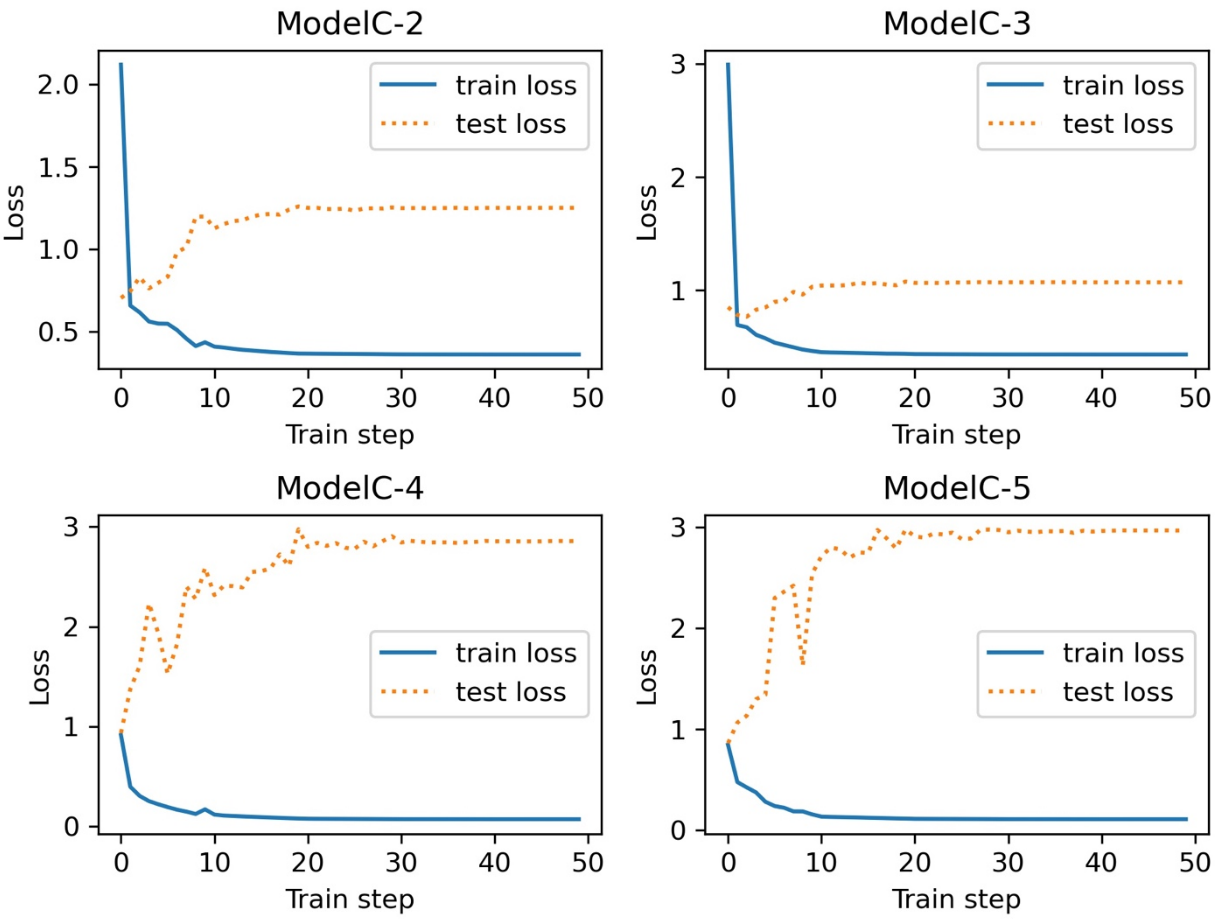

Supplementary Figure 21. Model C curve of changes during training on Epilepsy dataset 2.

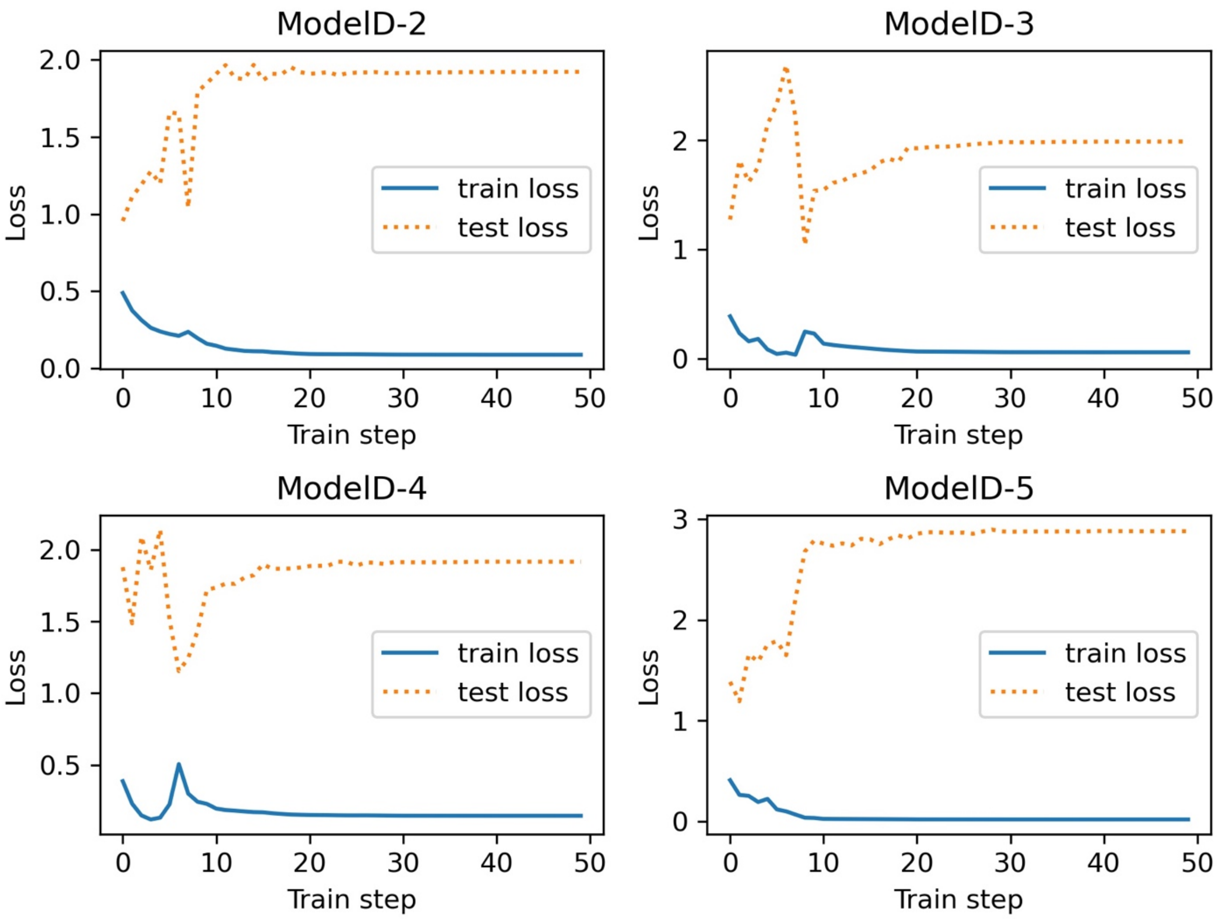

Supplementary Figure 22. Model D curve of changes during training on Epilepsy dataset 2.

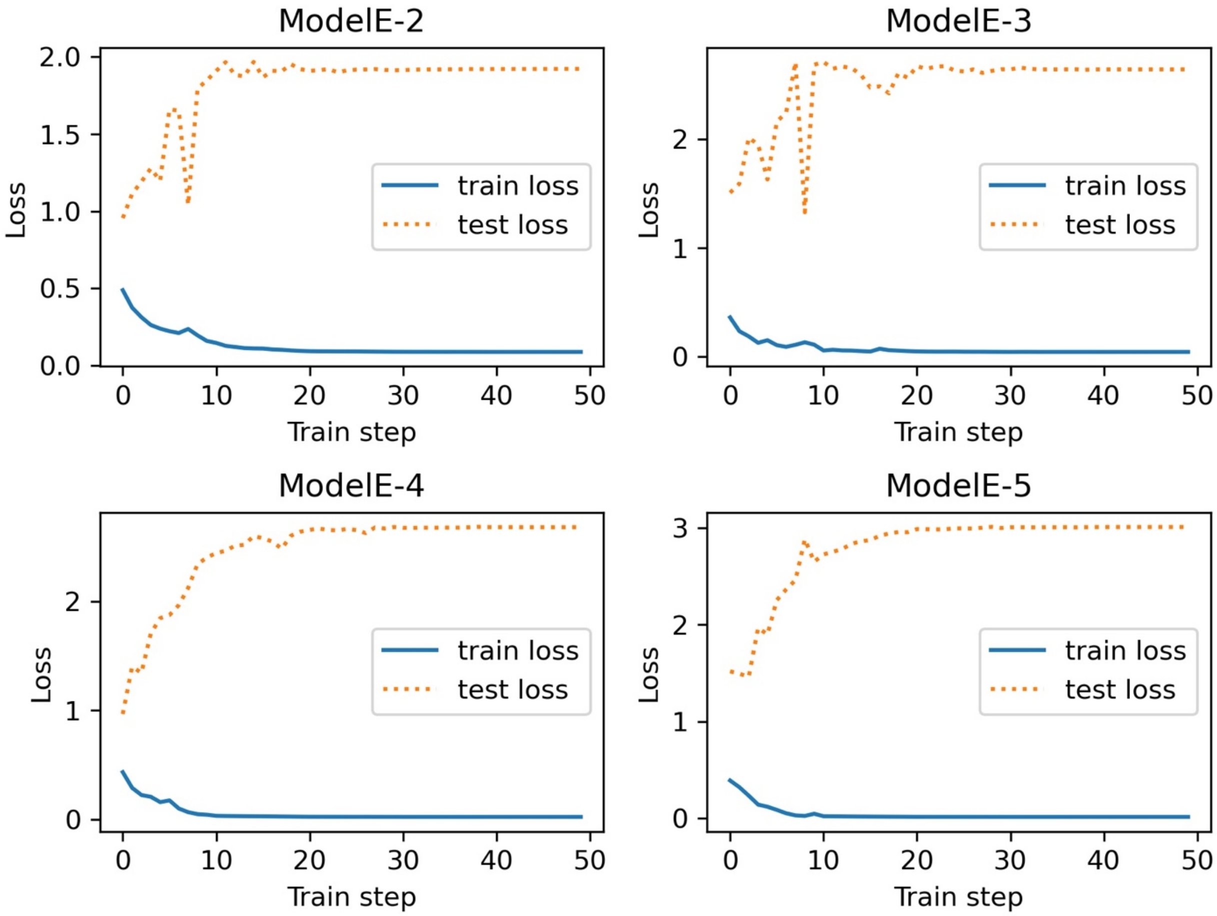

Supplementary Figure 23. Model E curve of changes during training on Epilepsy dataset 2.

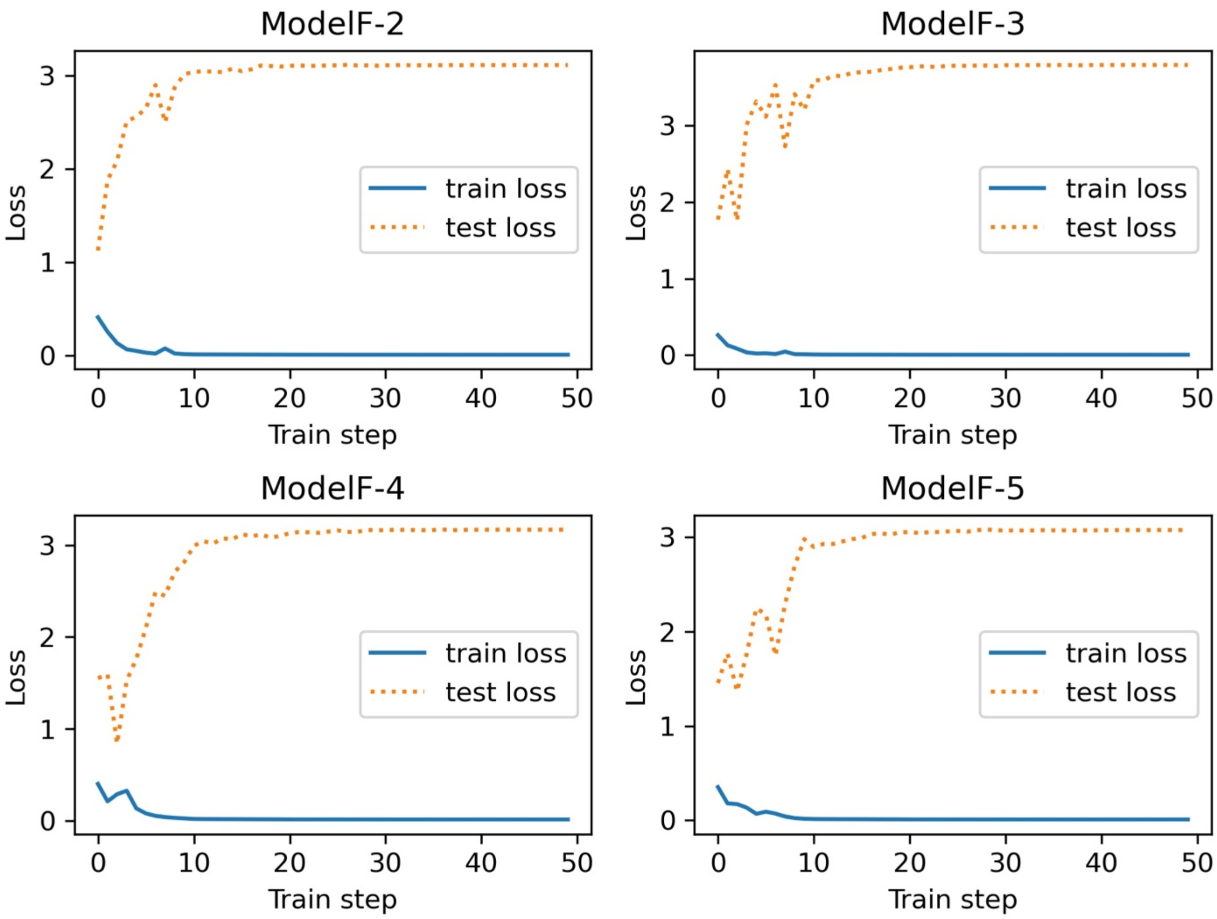

Supplementary Figure 24. Model F curve of changes during training on Epilepsy dataset 2.

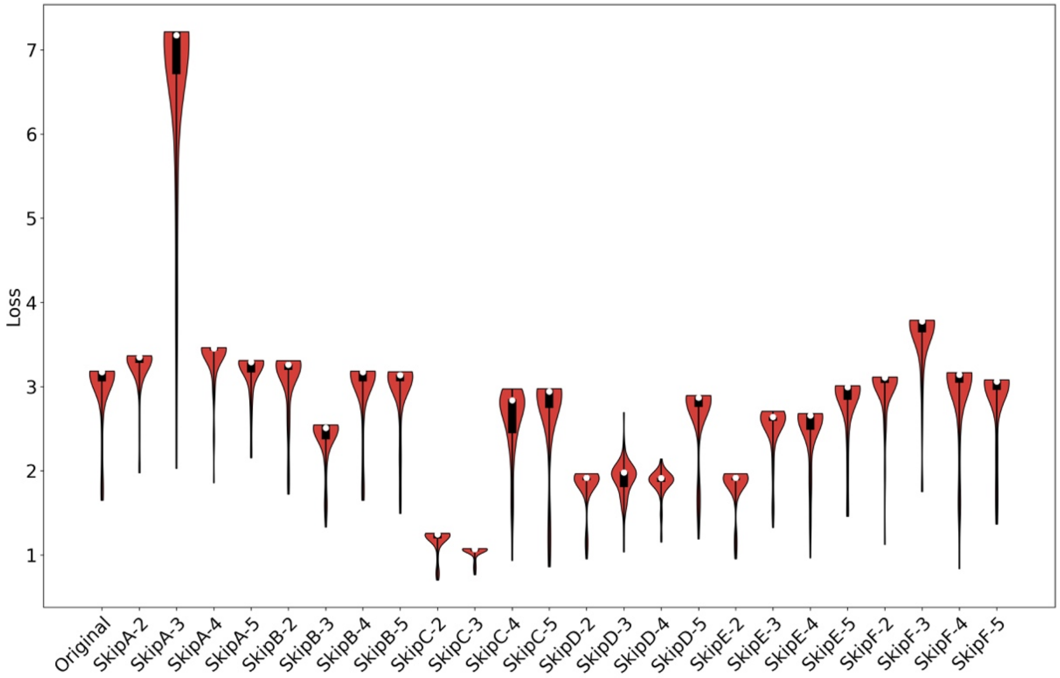

Supplementary Figure 25. Loss of classification of each model for Epilepsy dataset 2.

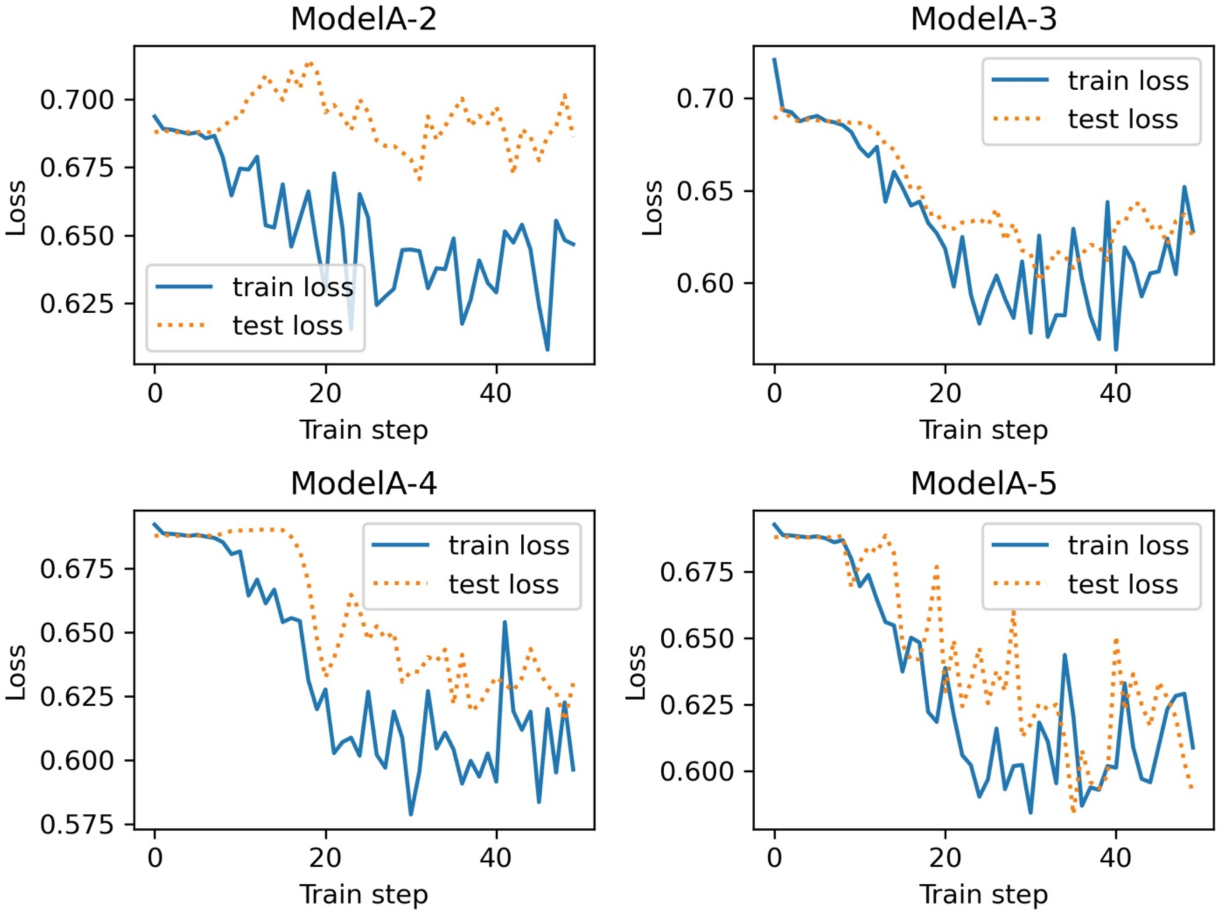

Supplementary Figure 26. Model A curve of changes during training on breast cancer dataset.

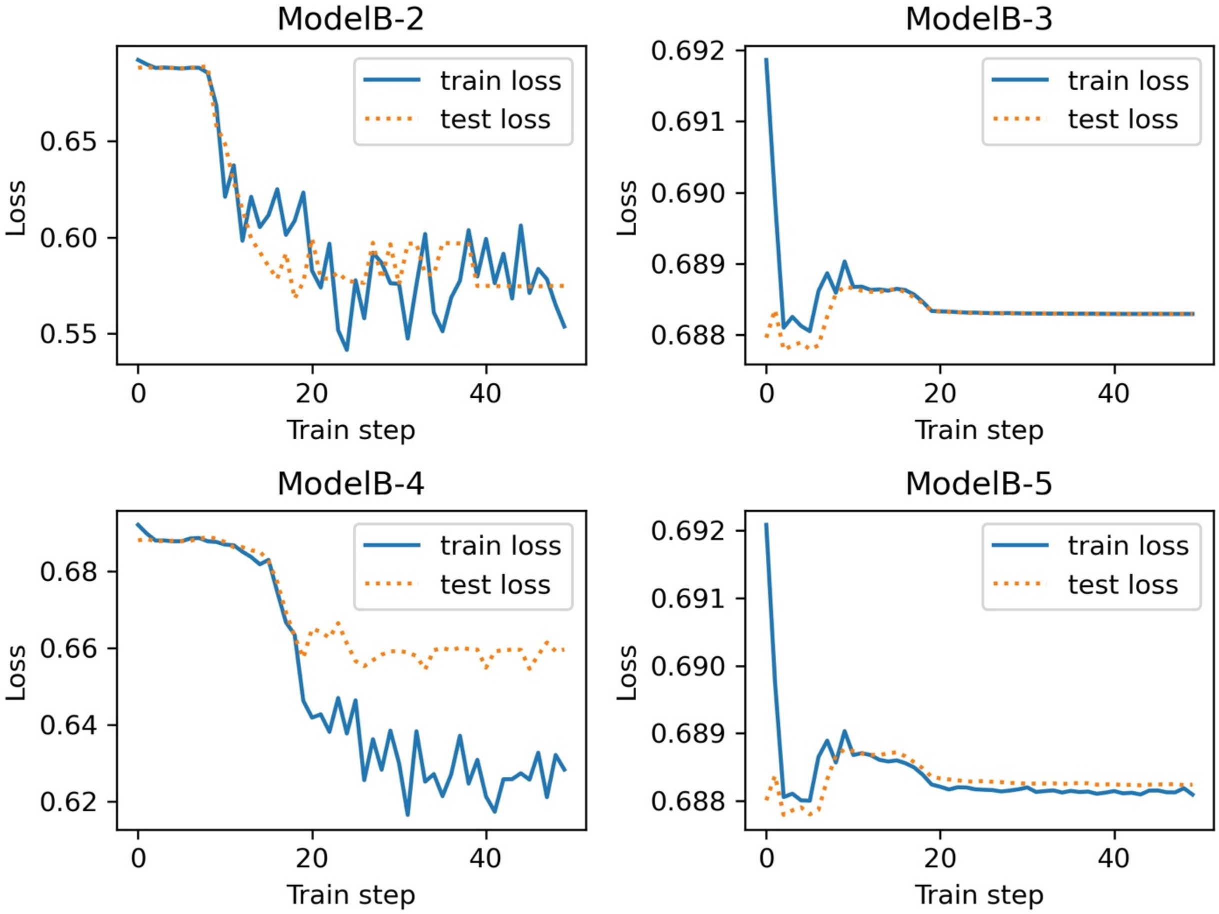

Supplementary Figure 27. Model B curve of changes during training on breast cancer dataset.

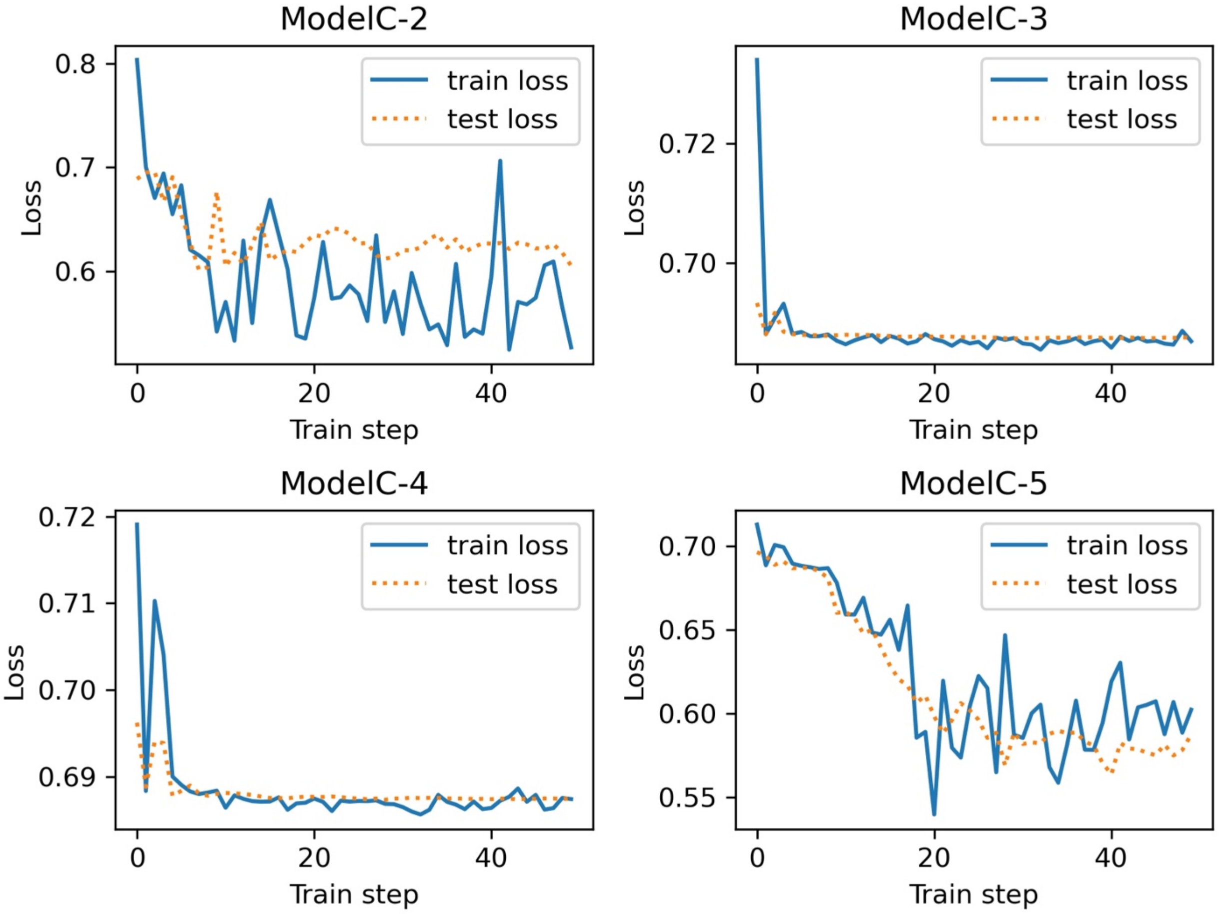

Supplementary Figure 28. Model C curve of changes during training on breast cancer dataset.

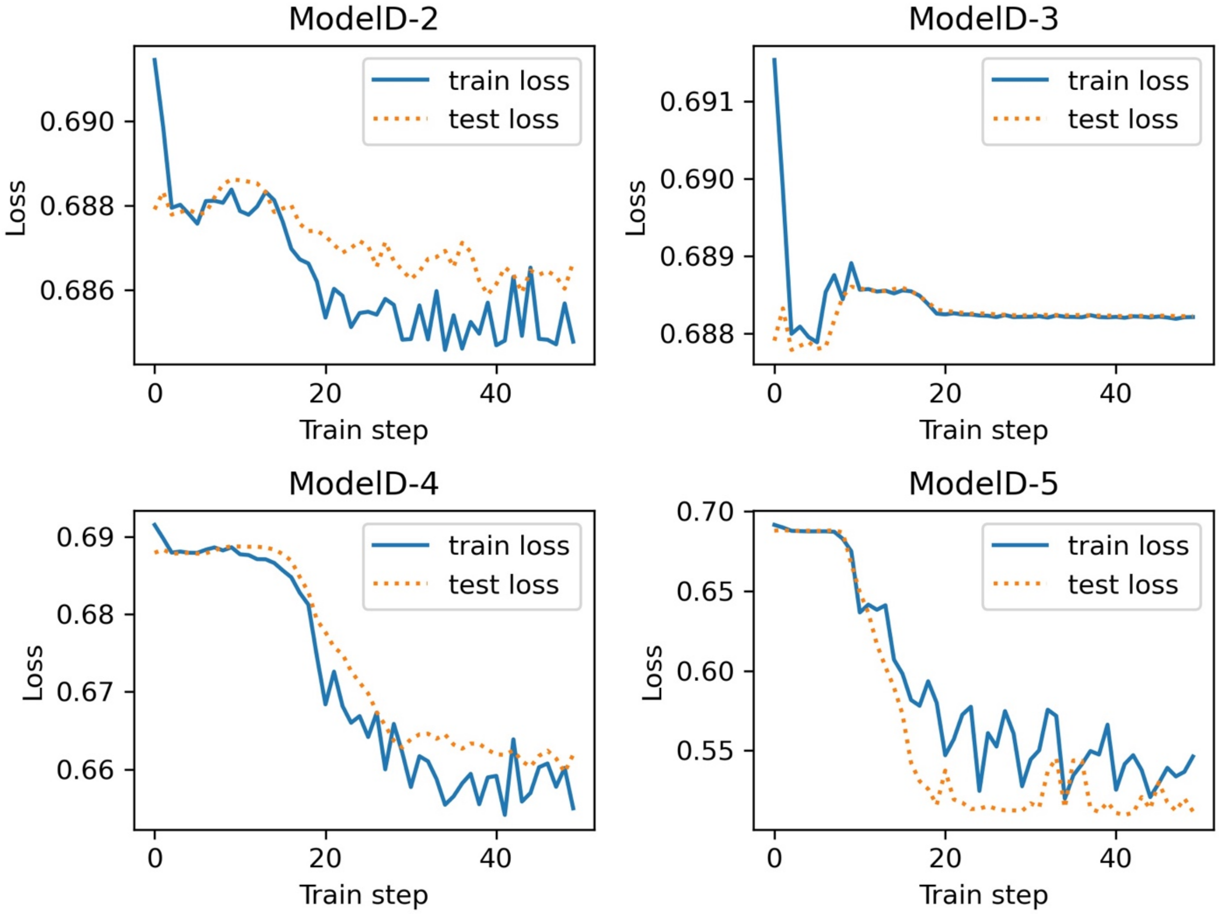

Supplementary Figure 29. Model D curve of changes during training on breast cancer dataset.

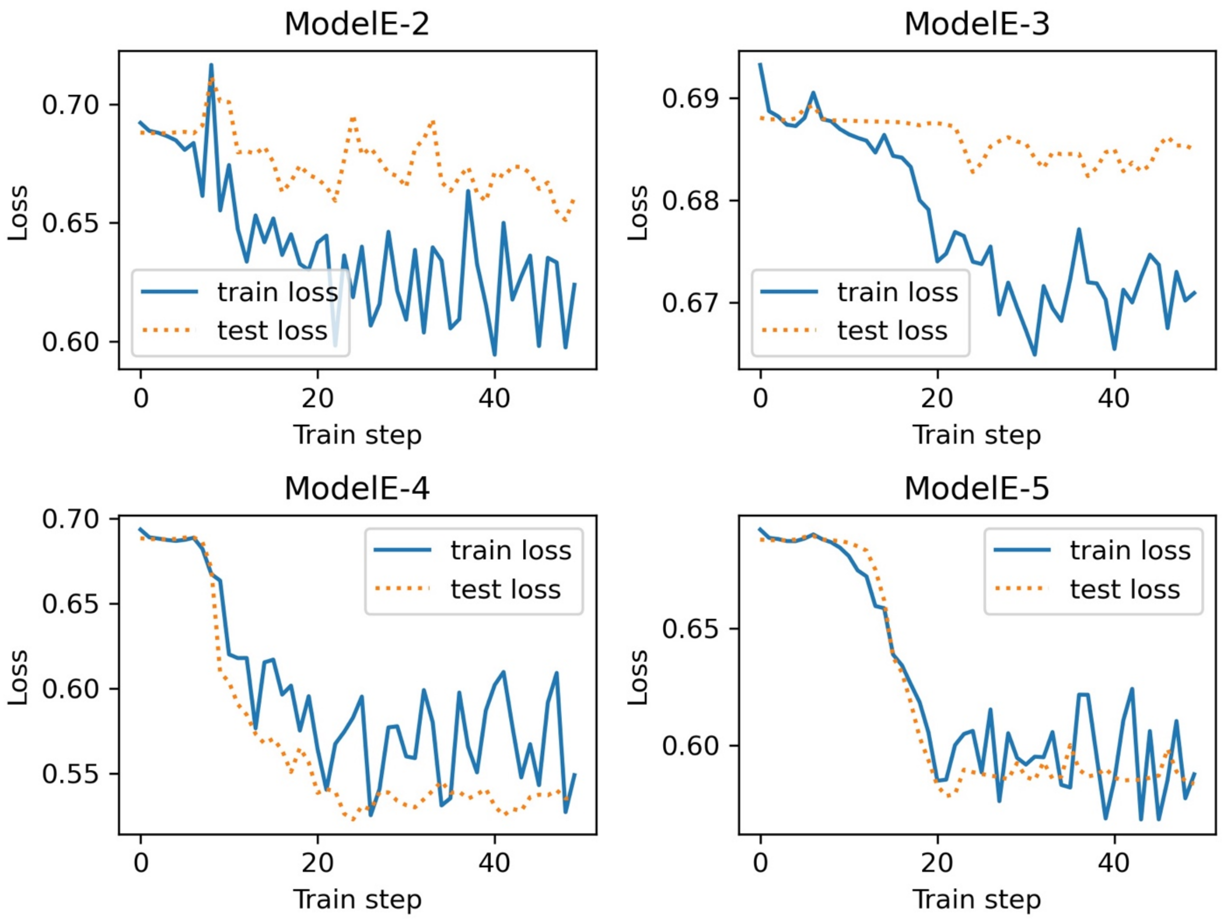

Supplementary Figure 30. Model E curve of changes during training on breast cancer dataset.

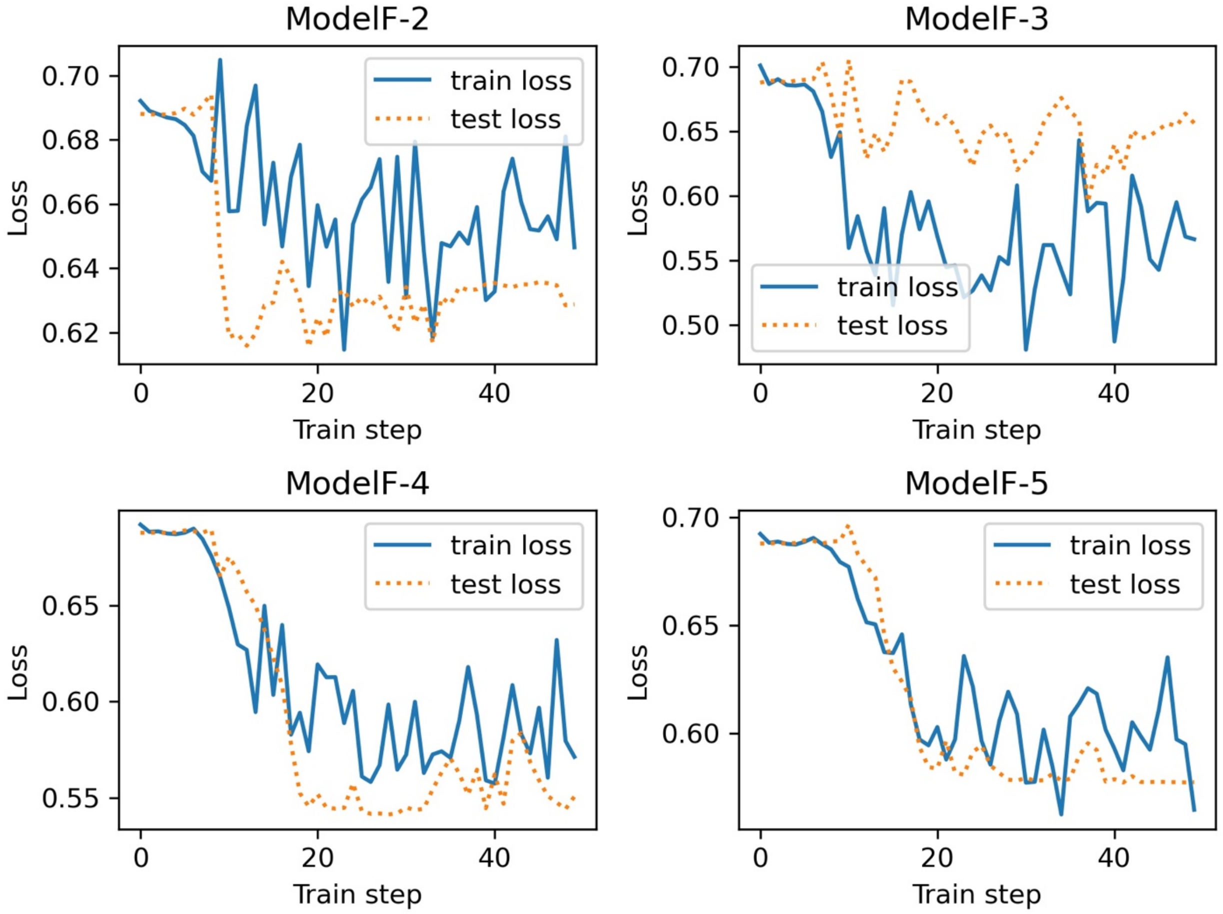

Supplementary Figure 31. Model F curve of changes during training on breast cancer dataset.

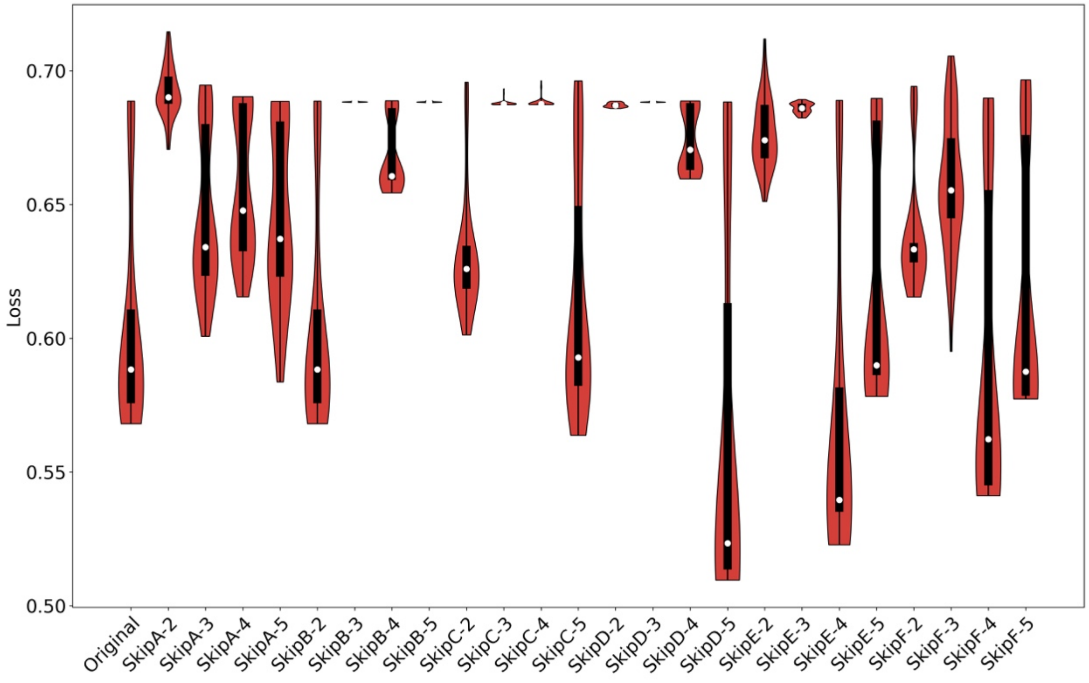

Supplementary Figure 32. Loss of classification of each model for breast cancer dataset.

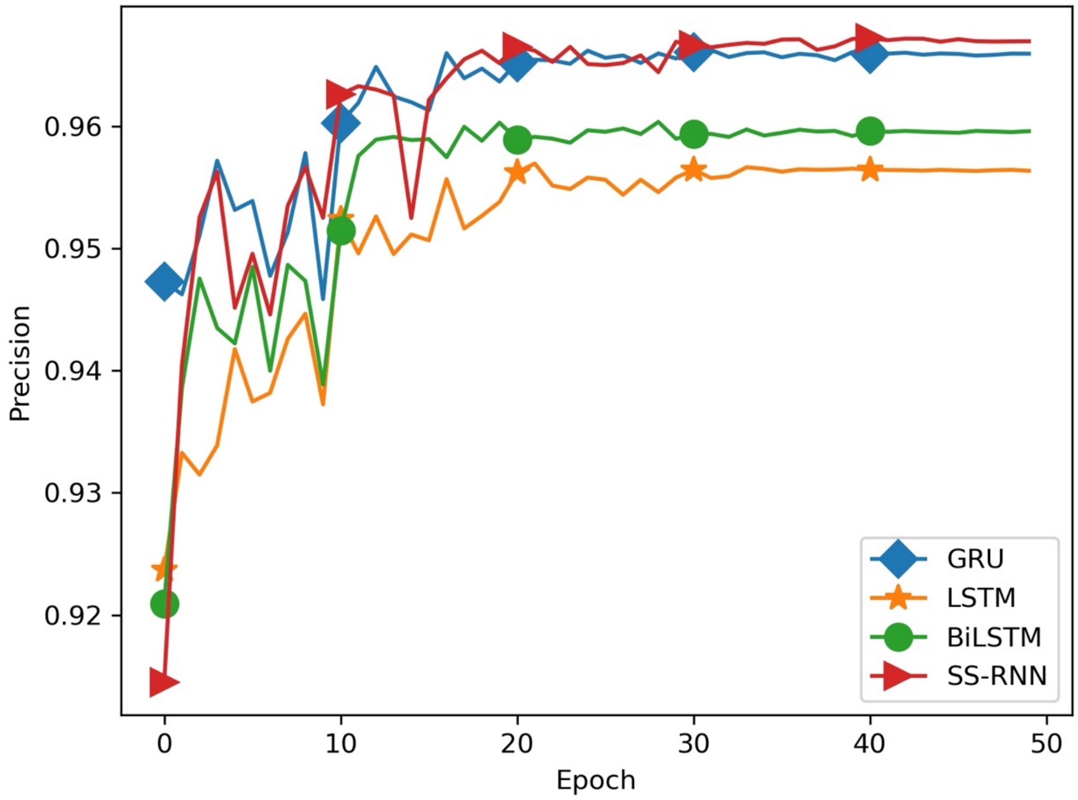

Supplementary Figure 33. Precision of the Arrhythmia dataset between LSTM, GRU, Bi-LSTM and ours SS-RNN (SkipA-3).

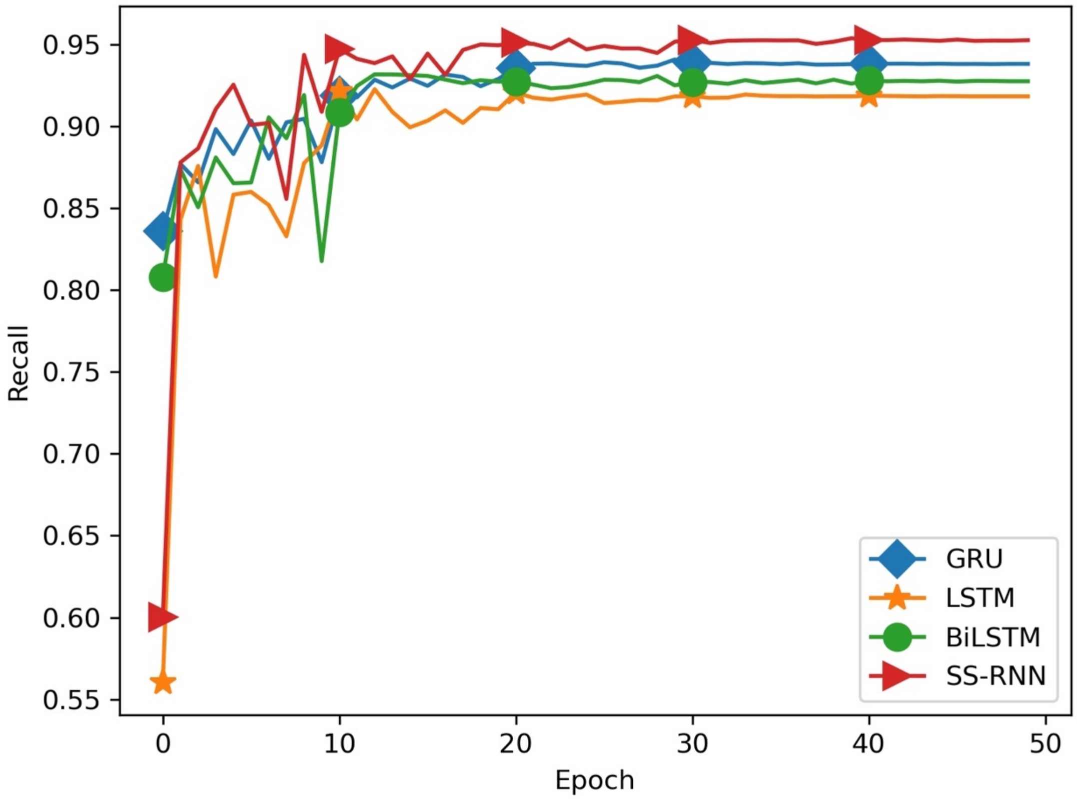

Supplementary Figure 34. Recall of the Arrhythmia dataset between LSTM, GRU, Bi-LSTM and ours SS-RNN (SkipA-3).

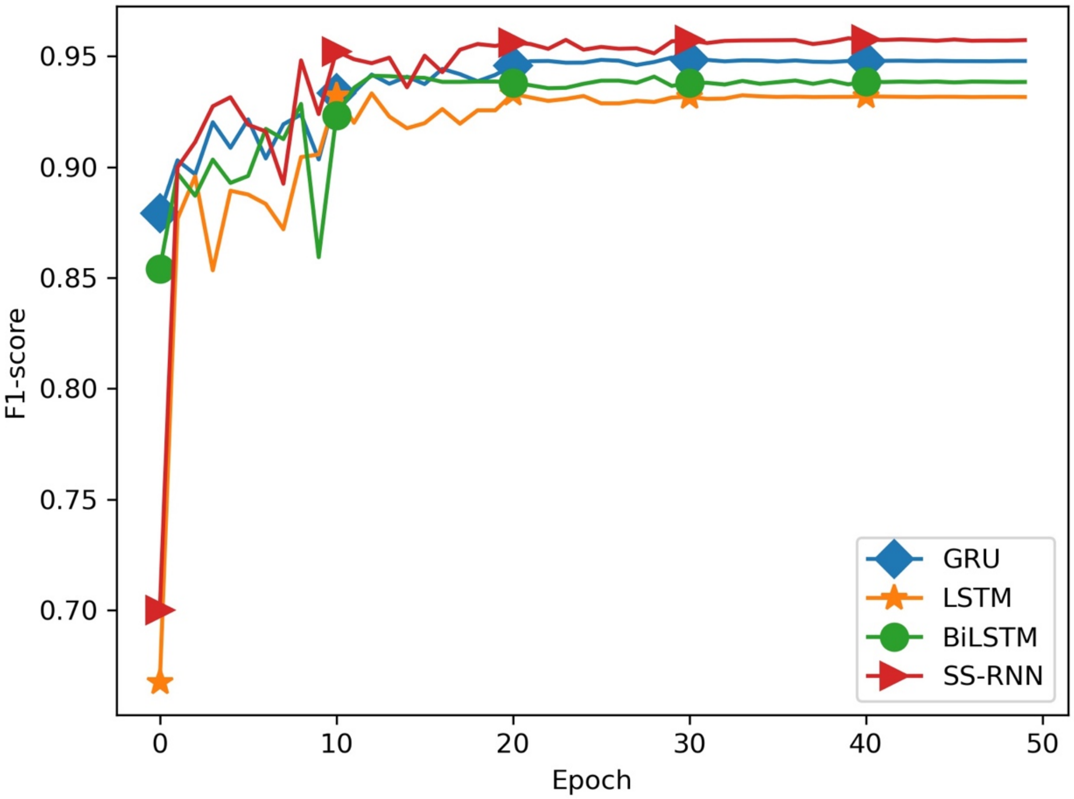

Supplementary Figure 35. F1-score of the Arrhythmia dataset between LSTM, GRU, Bi-LSTM and ours SS-RNN (SkipA-3).

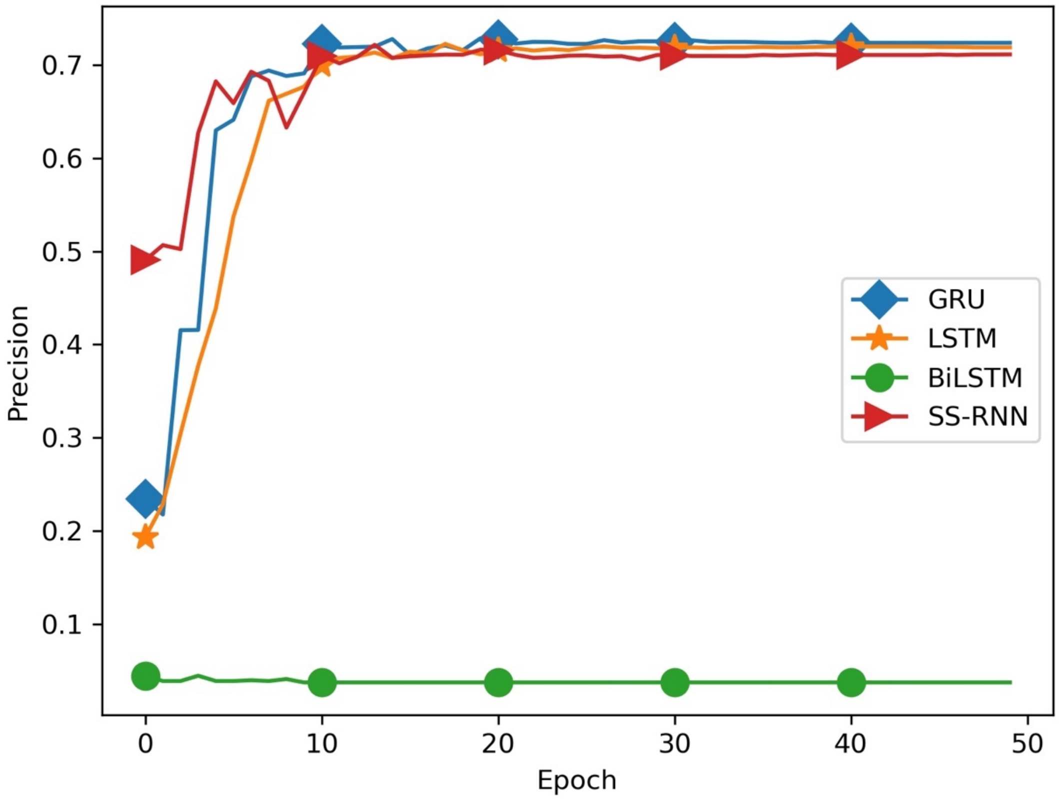

Supplementary Figure 36. Precision of the Epilepsy dataset 1 between LSTM, GRU, Bi-LSTM and ours SS-RNN (SkipA-3).

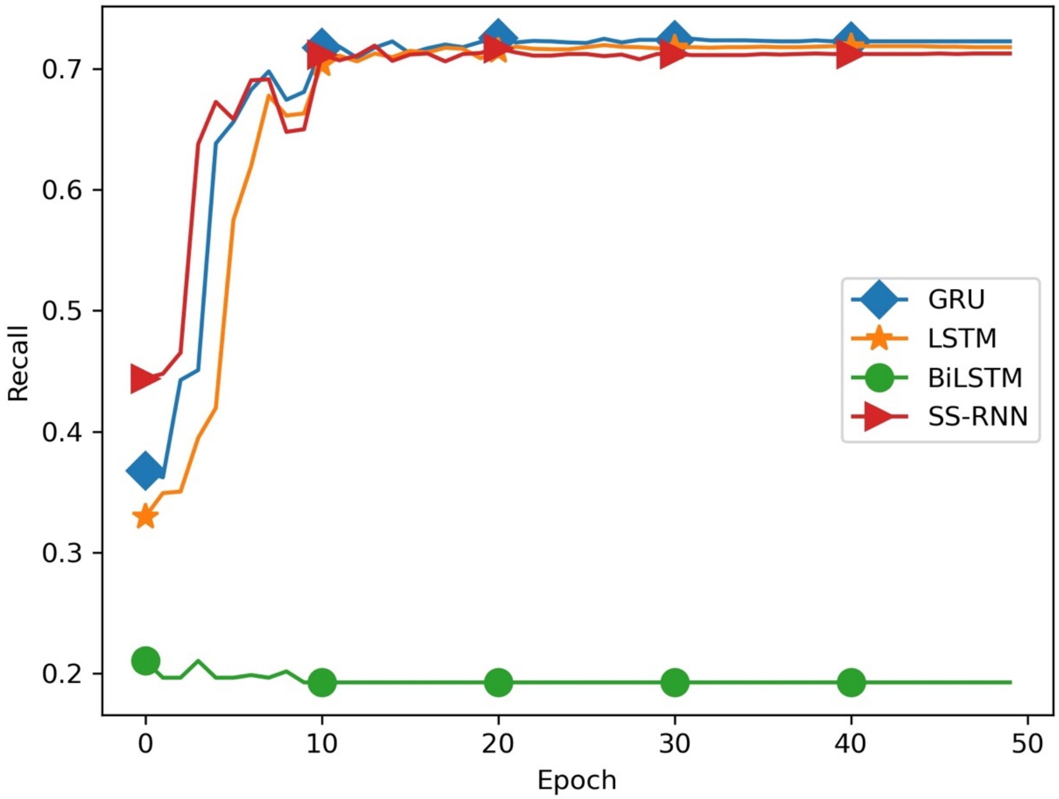

Supplementary Figure 37. Recall of the Epilepsy dataset 1 between LSTM, GRU, Bi-LSTM and ours SS-RNN (SkipA-3).

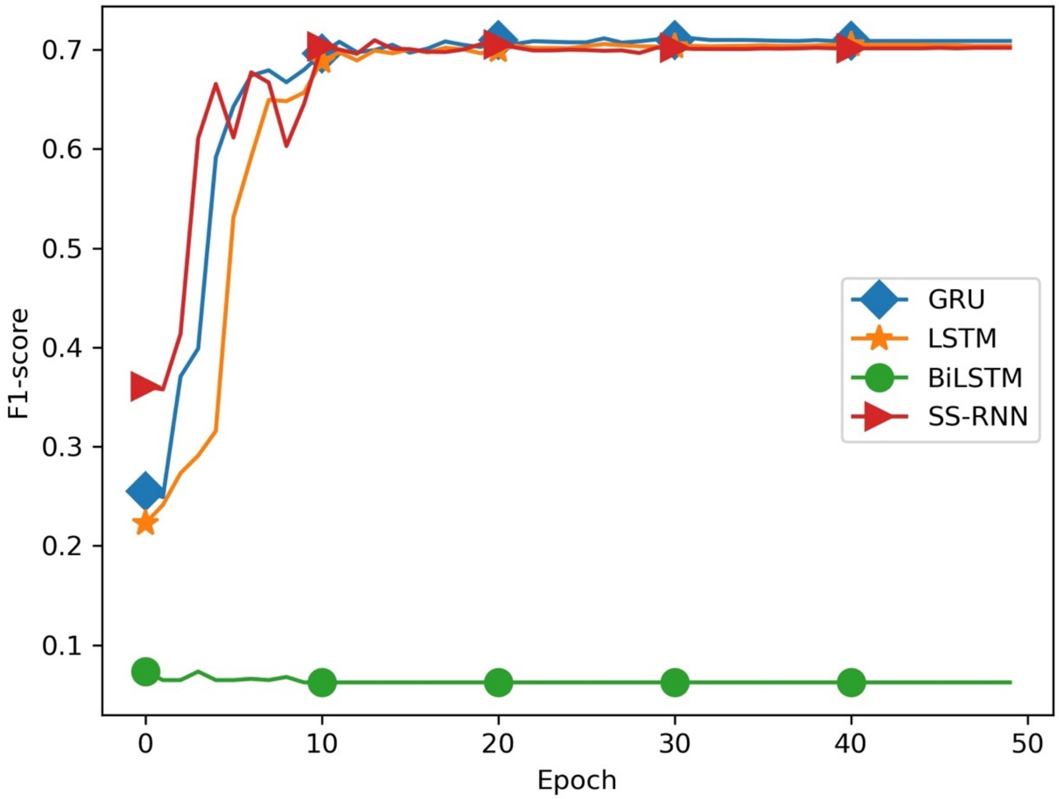

Supplementary Figure 38. F1-score of the Epilepsy dataset 1 between LSTM, GRU, Bi-LSTM and ours SS-RNN (SkipA-3).

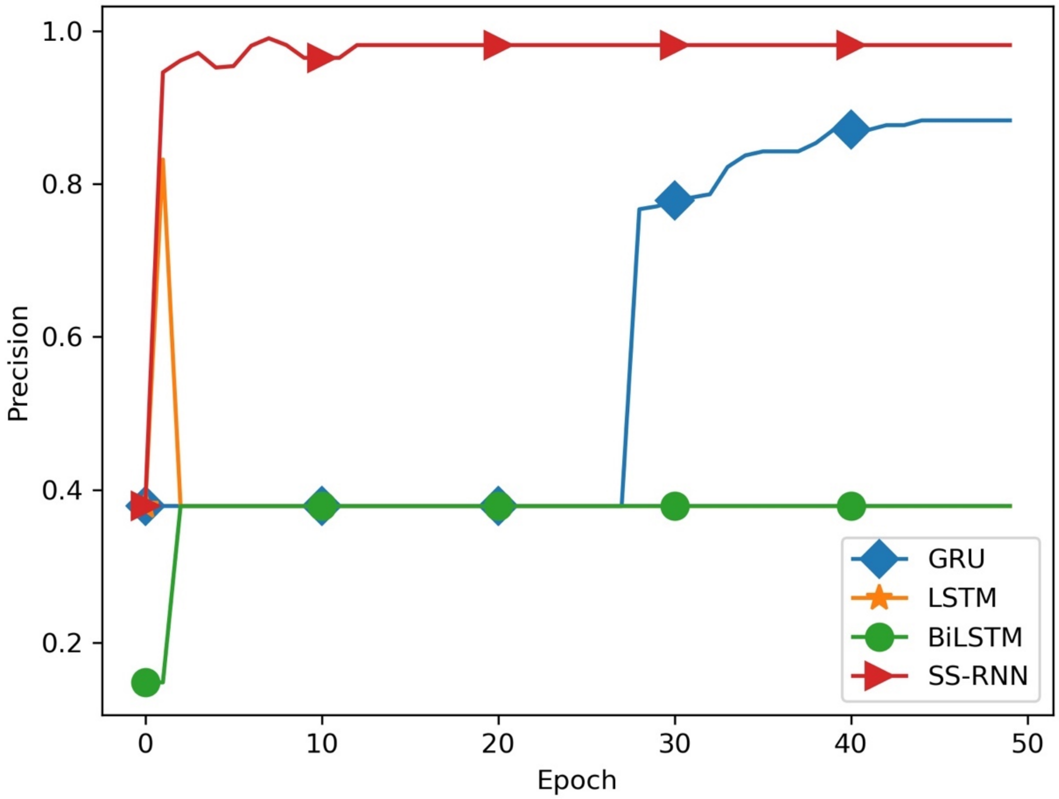

Supplementary Figure 39. Precision of the Diabetes dataset between LSTM, GRU, Bi-LSTM and ours SS-RNN (SkipA-3).

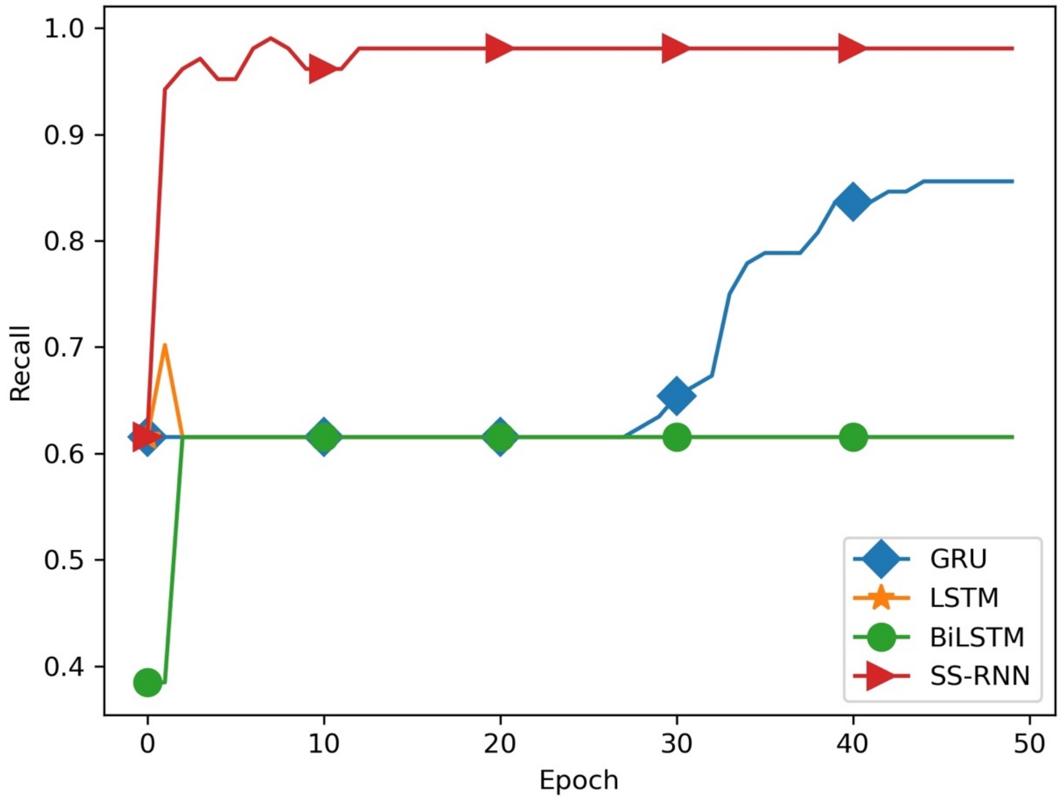

Supplementary Figure 40. Recall of the Diabetes dataset between LSTM, GRU, Bi-LSTM and ours SS-RNN (SkipA-3).

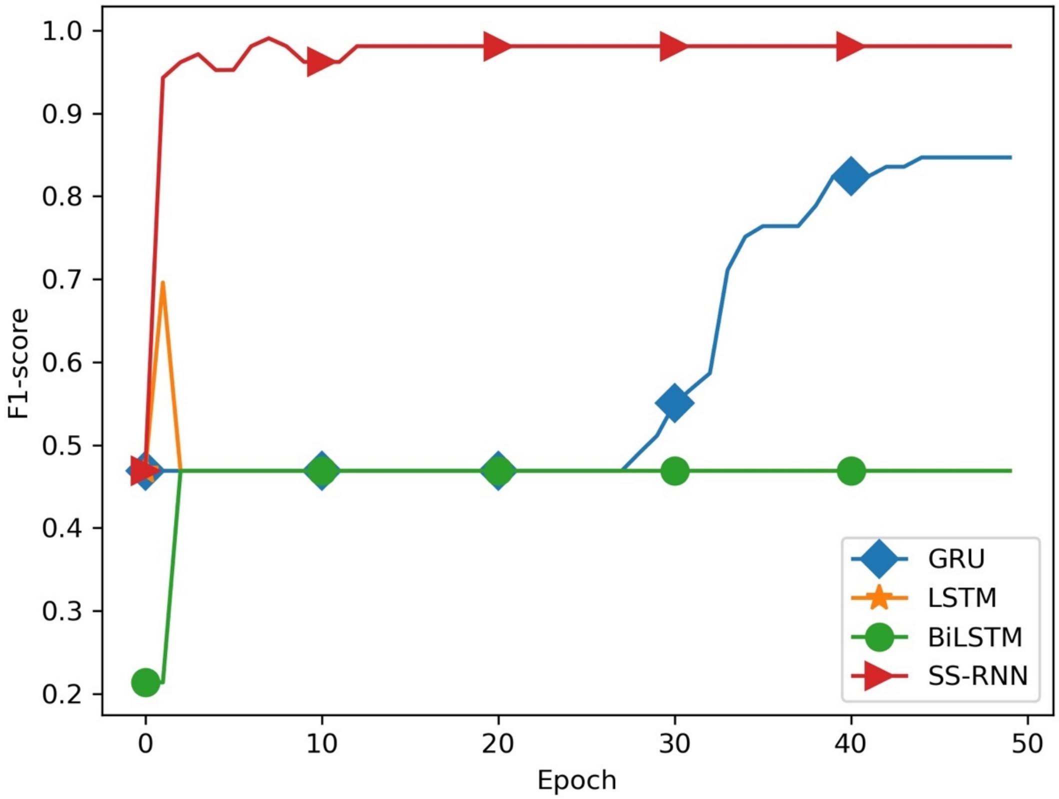

Supplementary Figure 41. F1-score of the Diabetes dataset between LSTM, GRU, Bi-LSTM and ours SS-RNN (SkipA-3).

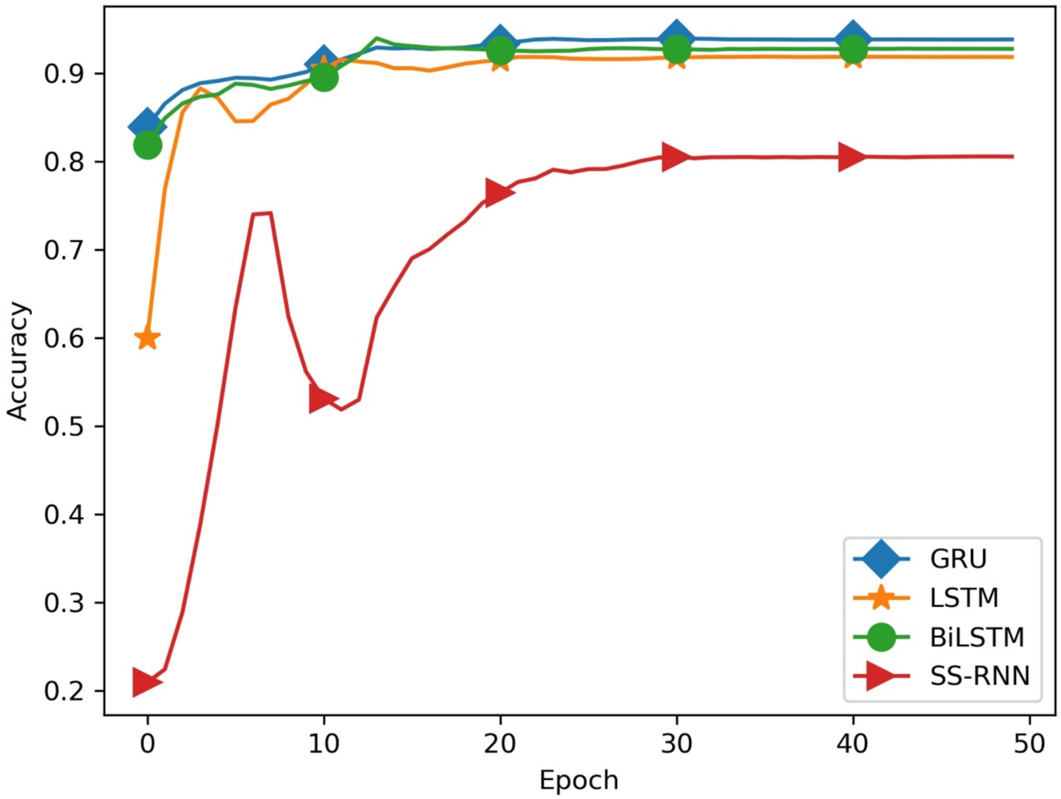

Supplementary Figure 42. Accuracy of the Arrhythmia dataset between LSTM, GRU, Bi-LSTM and ours SS-RNN (SkipC-5).

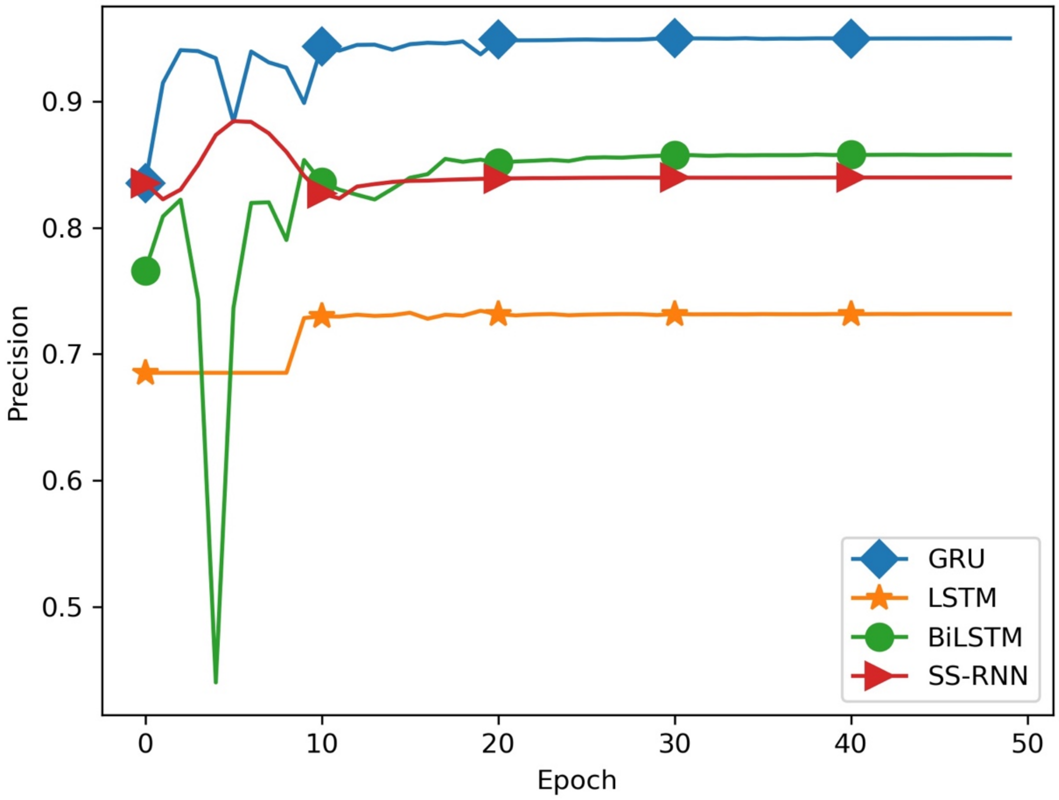

Supplementary Figure 43. Precision of the Arrhythmia dataset between LSTM, GRU, Bi-LSTM and ours SS-RNN (SkipC-5).

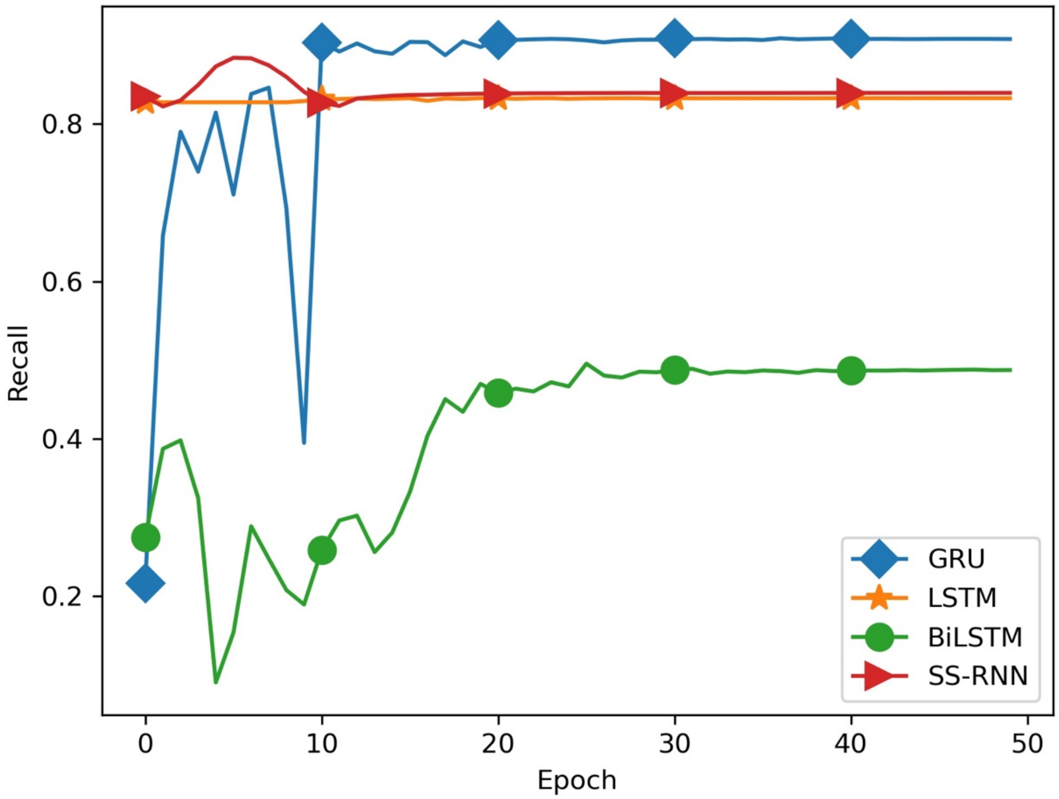

Supplementary Figure 44. Recall of the Arrhythmia dataset between LSTM, GRU, Bi-LSTM and ours SS-RNN (SkipC-5).

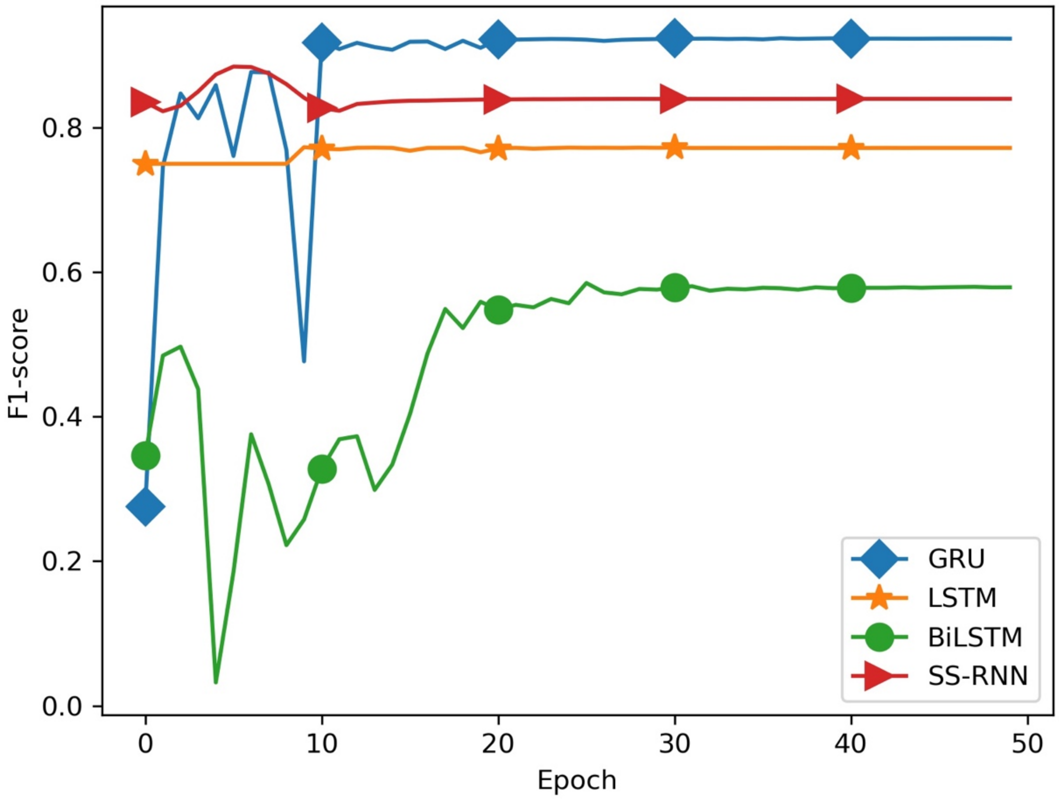

Supplementary Figure 45. F1-score of the Arrhythmia dataset between LSTM, GRU, Bi-LSTM and ours SS-RNN (SkipC-5).

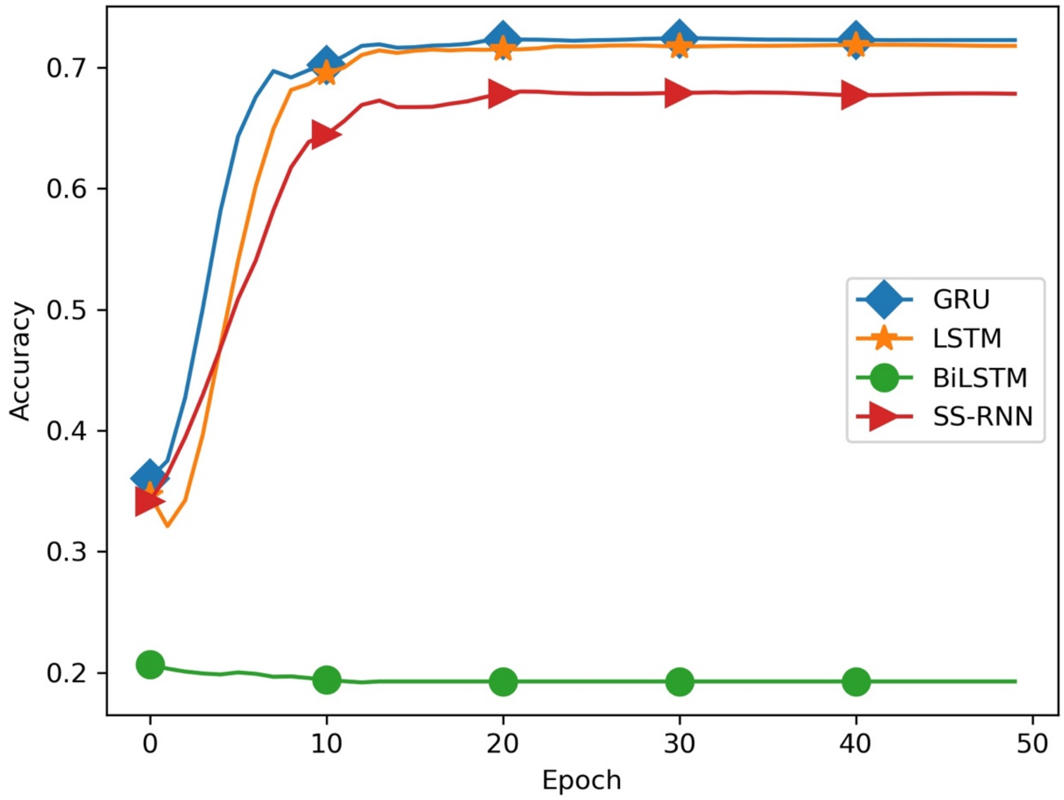

Supplementary Figure 46. Accuracy of the Epilepsy dataset 1 between LSTM, GRU, Bi-LSTM and ours SS-RNN (SkipC-5).

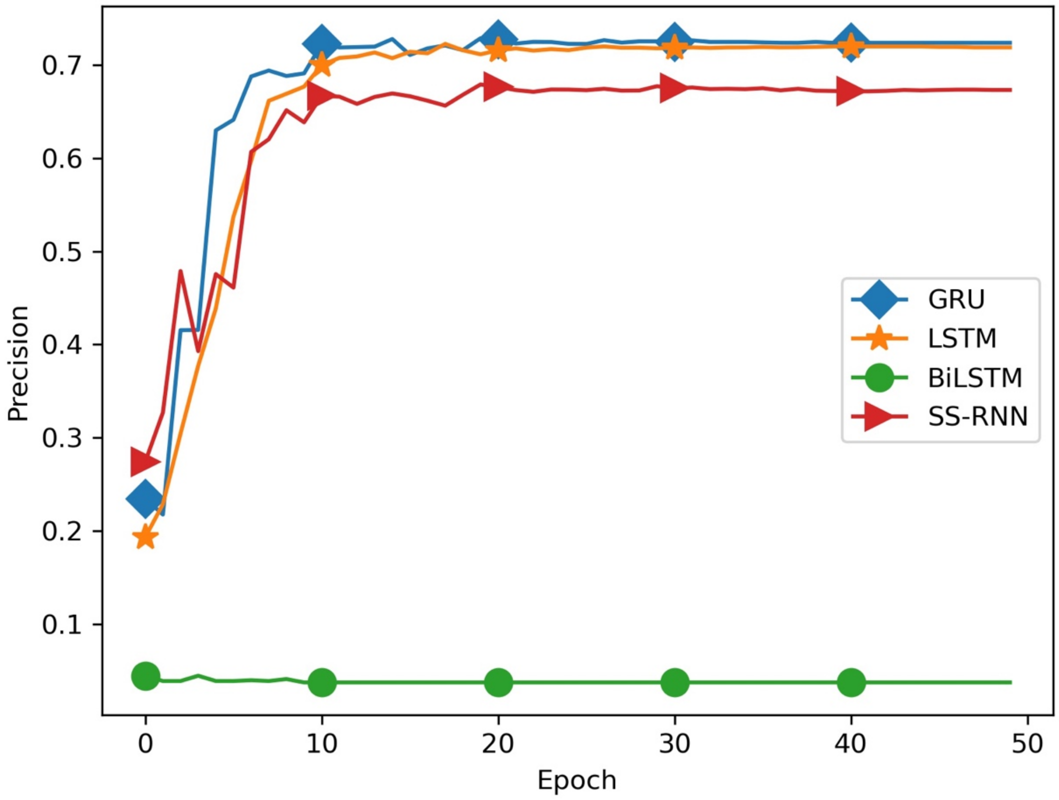

Supplementary Figure 47. Precision of the Epilepsy dataset 1 between LSTM, GRU, Bi-LSTM and ours SS-RNN (SkipC-5).

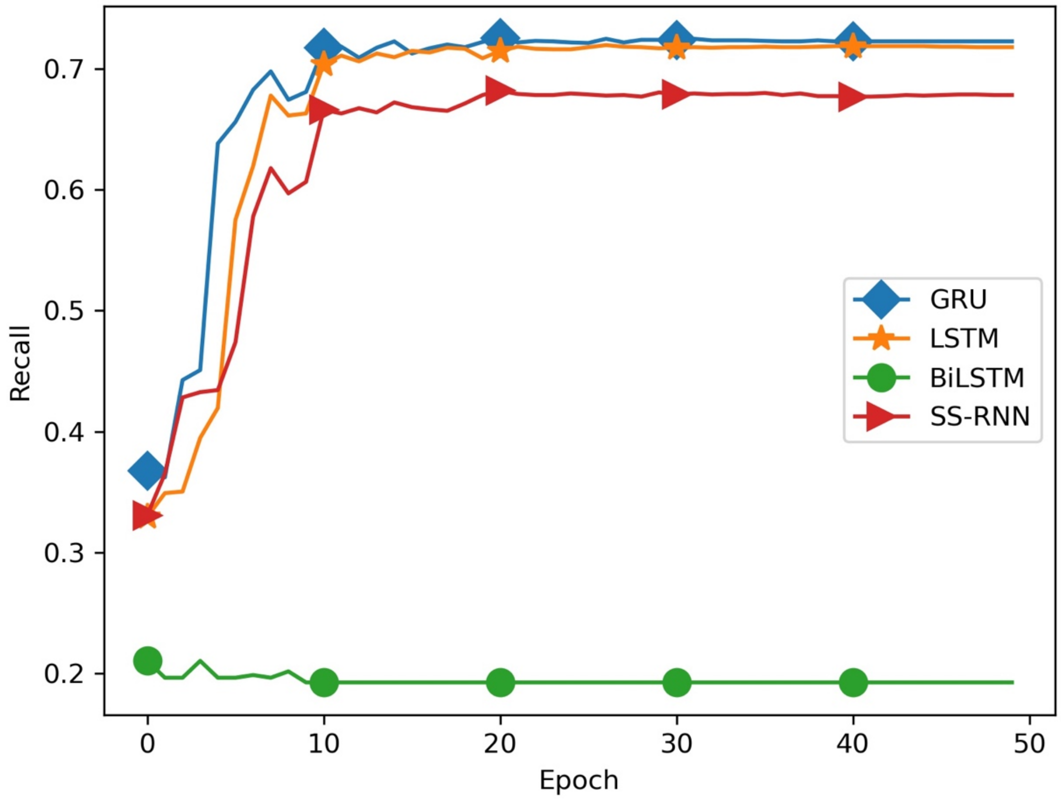

Supplementary Figure 48. Recall of the Epilepsy dataset 1 between LSTM, GRU, Bi-LSTM and ours SS-RNN (SkipC-5).

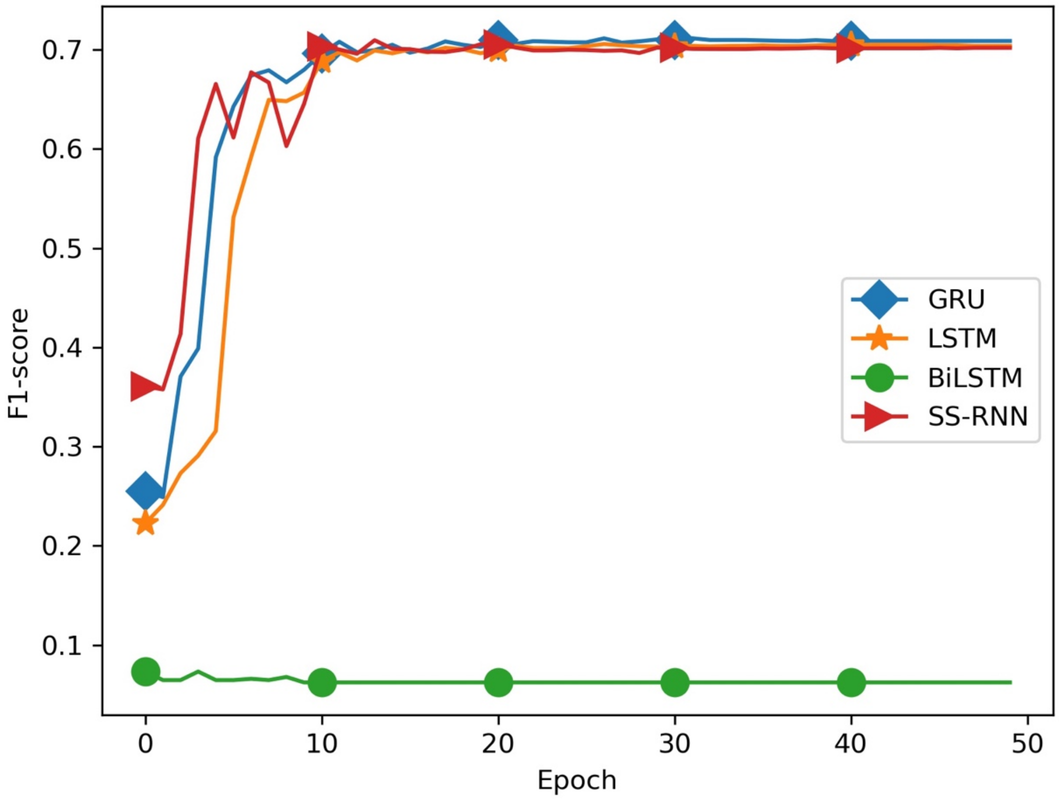

Supplementary Figure 49. F1-score of the Epilepsy dataset 1 between LSTM, GRU, Bi-LSTM and ours SS-RNN (SkipC-5).

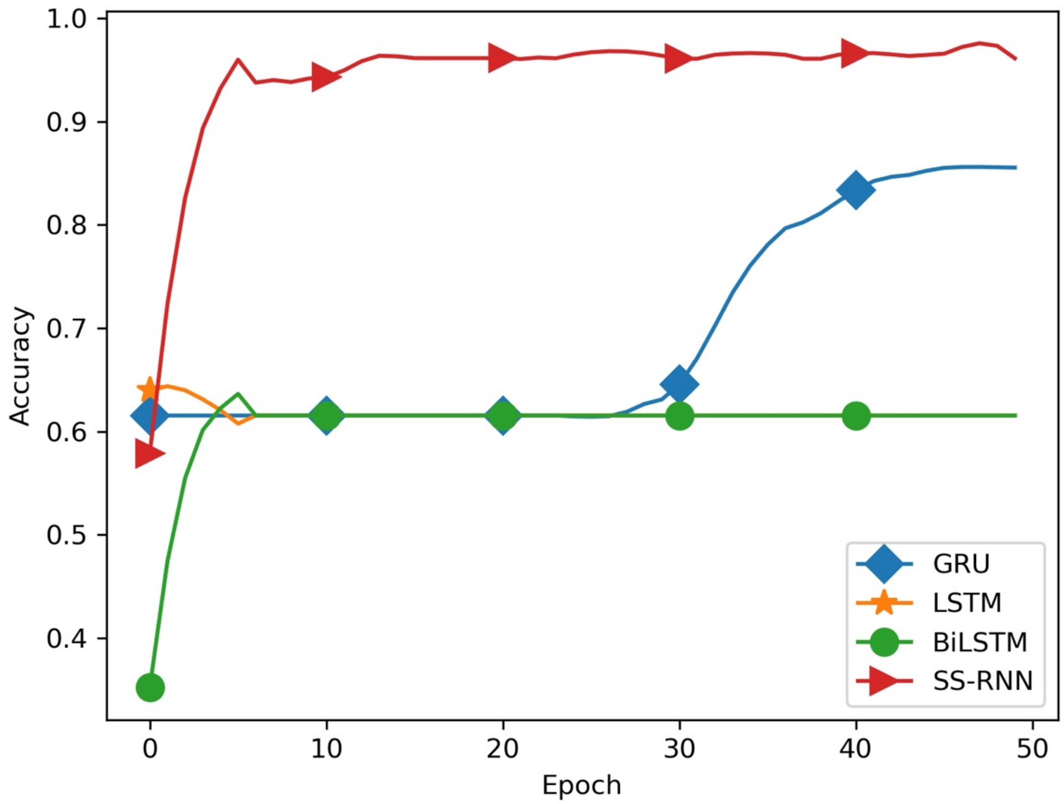

Supplementary Figure 50. Accuracy of the Diabetes dataset between LSTM, GRU, Bi-LSTM and ours SS-RNN (SkipC-5).

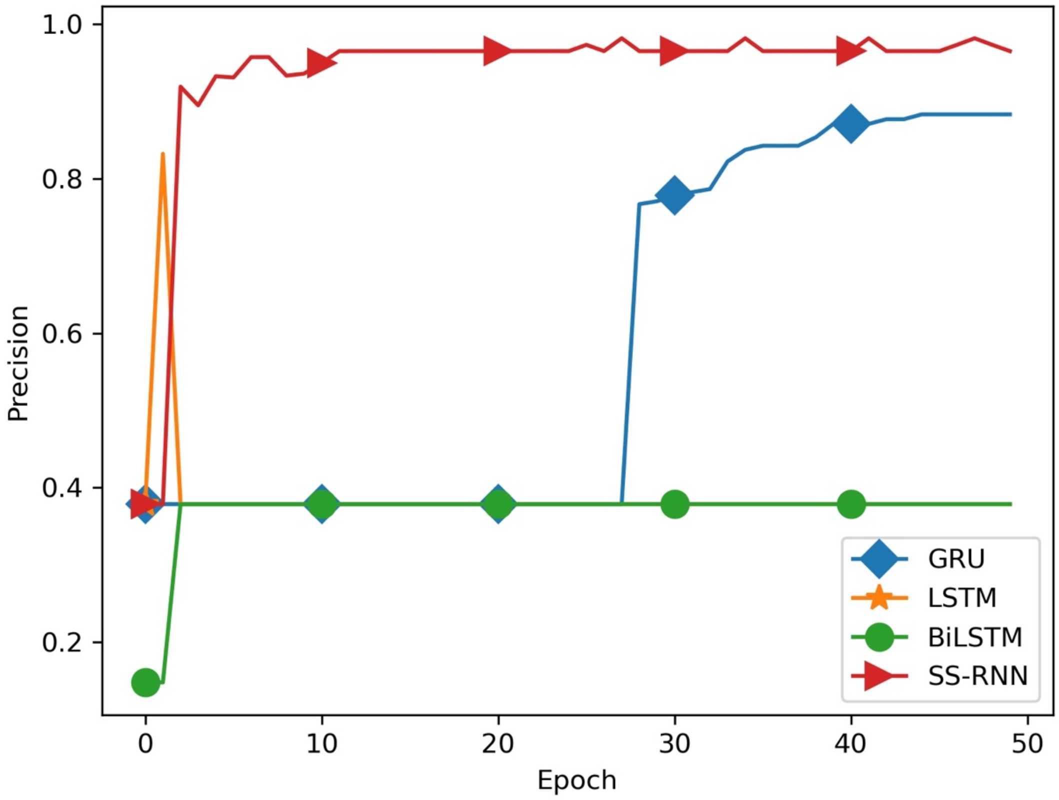

Supplementary Figure 51. Precision of the Diabetes dataset between LSTM, GRU, Bi-LSTM and ours SS-RNN (SkipC-5).

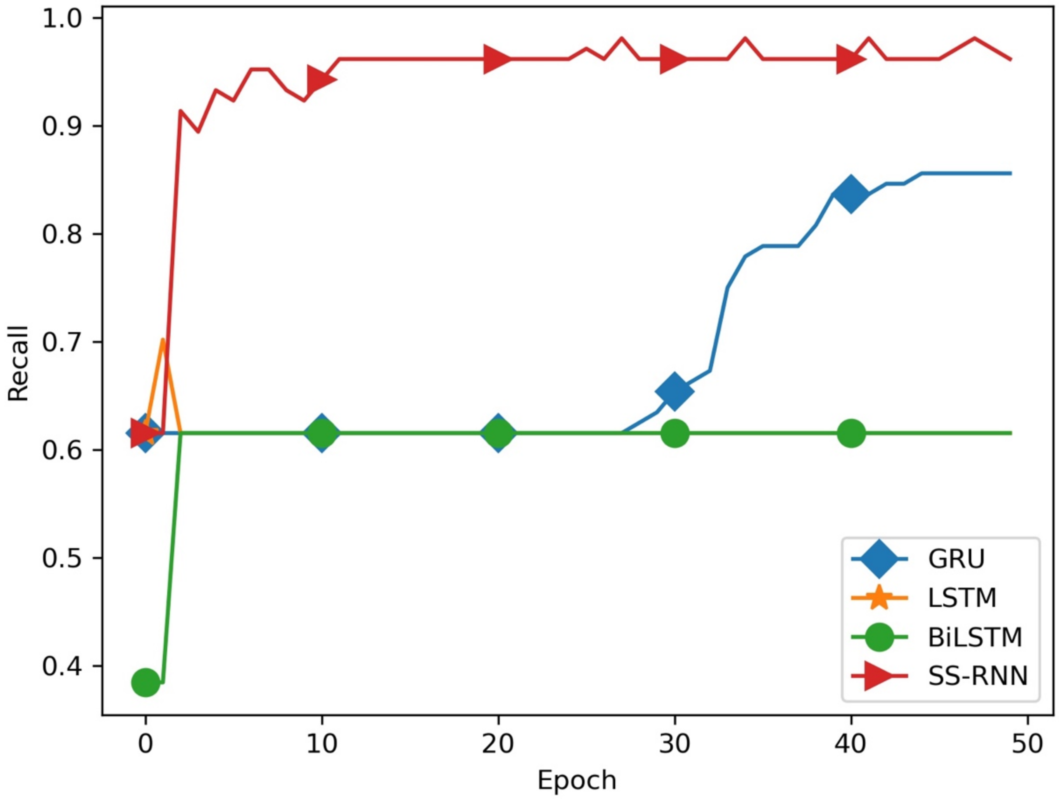

Supplementary Figure 52. Recall of the Diabetes dataset between LSTM, GRU, Bi-LSTM and ours SS-RNN (SkipC-5).

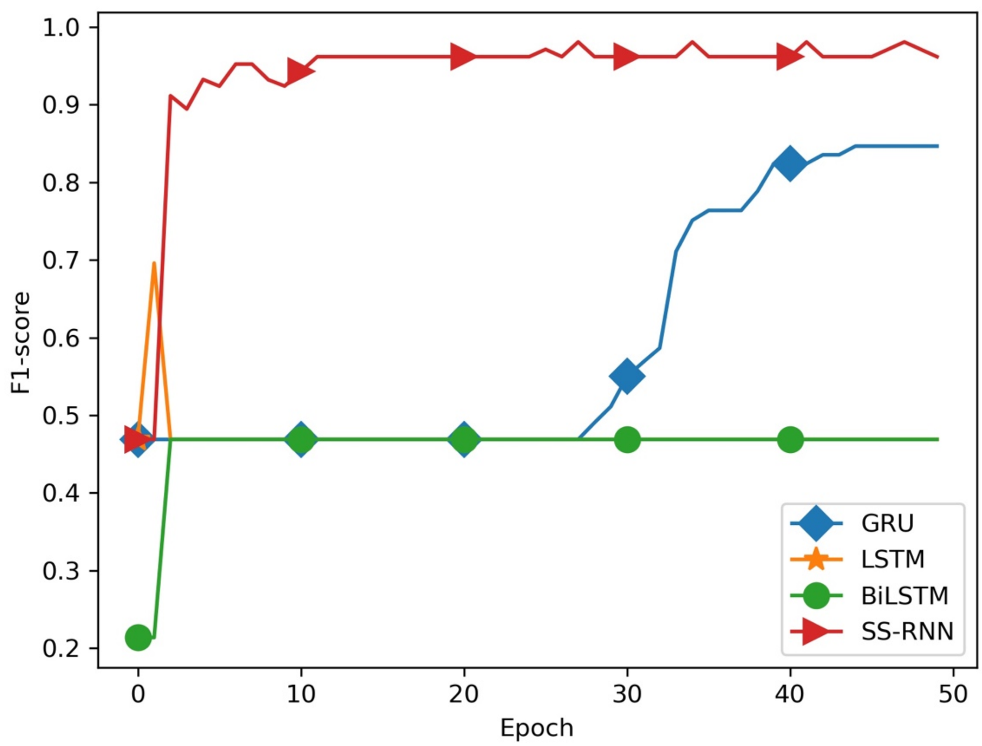

Supplementary Figure 53. F1-score of the Diabetes dataset between LSTM, GRU, Bi-LSTM and ours SS-RNN (SkipC-5).

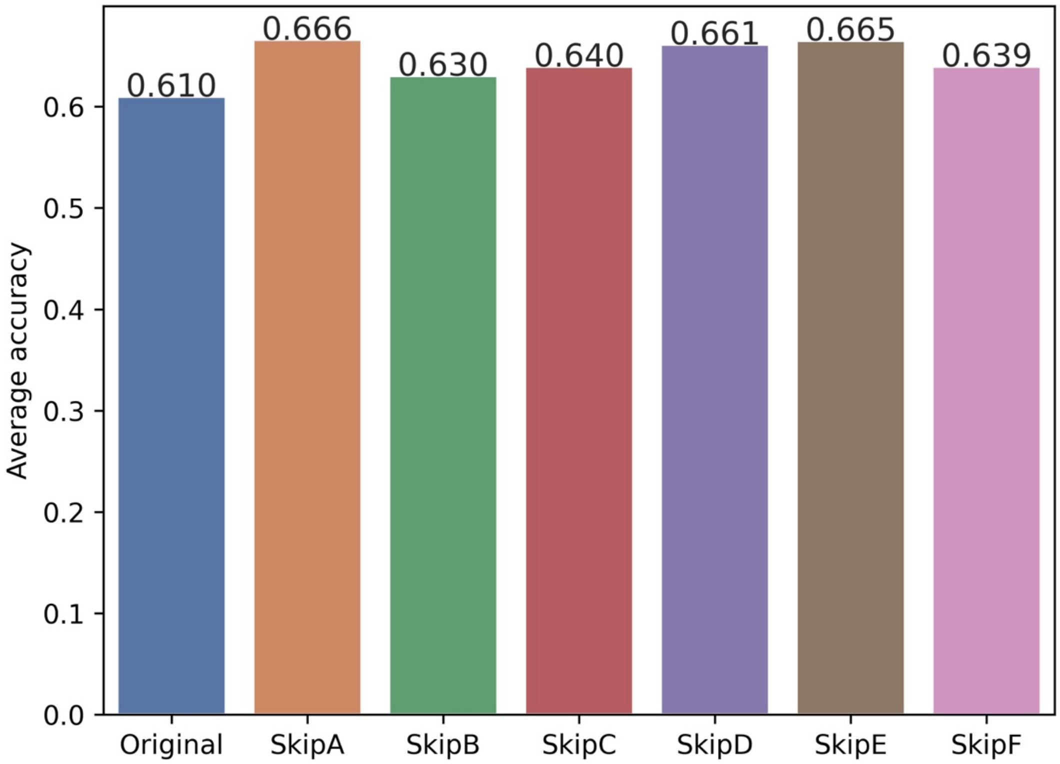

Supplementary Figure 54. Average accuracy of the Arrhythmia dataset, Epilepsy dataset 1, Epilepsy dataset 2, Diabetes dataset, and Breast cancer dataset between the original LSTM model and our six models without batchsize tuned.

4 Dimension change of data

Taking the Diabetes dataset as an example, the feature number of the input data is 16, and then the 128 features are extracted by the feature extractor, the batchsize is 20 (Fig. S55). High-dimensional features contain more information than low-dimensional features. The larger the feature dimension is, the more corresponding optimization parameters are, and the stronger the fitting ability of the neural network will be. In this model, feature extractor is added to raise dimension of data. And then we reshape the dimension into 32*4, and 32 is input into SS-LSTM as the time step. The final classification number is 2. The dimension changes of this dataset in the model are as follows:

16->128->64->32->16->32->64->128->32*4->32*18->32*8->32*5(5)->2.

Before the output layer, we take the output value at time 32, so the dimension become 5.

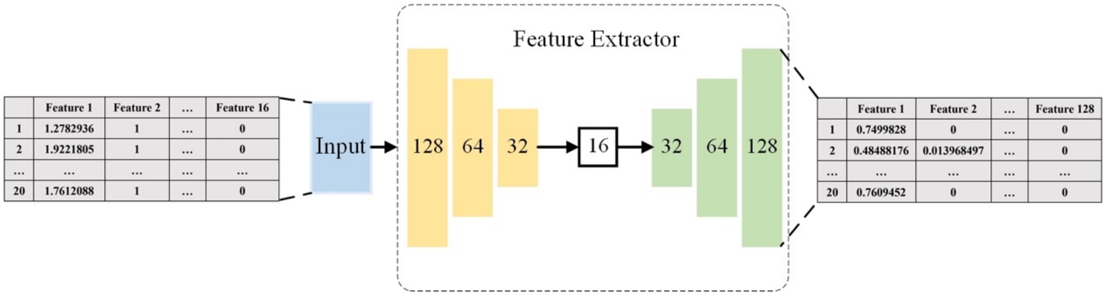

Supplementary Figure 55. The framework of the Diabetes dataset processing.
